# Risk factors for infection in older adults with home care: a mixed methods systematic review with meta-analysis

**DOI:** 10.1186/s12889-025-22538-1

**Published:** 2025-05-03

**Authors:** Ann Liljas, Madelene Barboza, Carmela Basanisi, Gabrielle Muzzi, Abaynesh Haftu Nigussie, Janne Agerholm, Bo Burström, Ester Gubi

**Affiliations:** 1 Department of Global Public Health, Karolinska Institutet , 171 77 Stockholm, Sweden; 2 Department of Health Sciences, Western University, London, ON N6 A 3 K7 Canada; 3 Aging Research Center, Department of Neurobiology, Care Sciences and Society, Karolinska Institutet, Stockholm, Sweden

**Keywords:** Home care, Older adults, Risk factors

## Abstract

Worldwide, home care in the form of home healthcare and home help, has become increasingly more available. This systematic review aims to provide a deeper understanding of factors that are of particular importance for infection control in the home care setting for older adults. Five databases were searched (MEDLINE, Embase, ProQuest, Web of Science, CINAHL) for eligible studies using any research design reporting on individual, medical, behavioral and environmental factors. Retrieved studies were screened and assessed for quality. The Joanna Briggs Institute manual guided the research process and the work of generating a synthesis. Qualitative findings were compiled using meta-aggregation. For quantitative evidence, meta-analyses were conducted when possible. Of 19,484 unique records, 27 studies (7 cohort studies, 9 cross-sectional and 11 qualitative) were included in the review. Risk factors for infection reported in the quantitative studies referred to individual, medical, social, behavioral, environmental, and organisational aspects. Meta-analyses showed associations between urinary catheter use (OR 3.97, 95%CI 2.56–6.15) and limited mobility (OR 1.49, 95%CI 1.31–1.68), respectively, and risk of infection. Pooled ORs of urinary incontinence and risk of infection were not statistically significant. Findings from the qualitative studies covered perceived and observed risk factors to infection control and prevention. The evidence resulted in five synthesised findings covering attitudes, behaviors, home environment, personal interactions, lack of equipment, unsafe disposal of material, pets, unsafe practices and procedures, and lack of training. The combined quantitative and qualitative evidence sheds light on separate yet interconnected elements of risks for infection that may reinforce each other, potentially exposing vulnerable older adults to amplified risks.

**Supplementary Information:**

The online version contains supplementary material available at 10.1186/s12889-025-22538-1.

## Introduction

Worldwide, more people are living longer with chronic conditions. This has increased the demand for care, which in turn has increasingly become more available in the older person’s home [1, 2]. Providing home care (home healthcare and/or home help) implies challenges, including factors that influence the risk of infection among older adults receiving care in their homes. These factors refer to the individual characteristics, such as older age, previous hospitalisation, poor health [3], a history of prior infections [4], and poor cognition [5]. Furthermore, behaviours, beliefs and attitudes have been reported to affect the risks of infection among older adults with home healthcare, including lack of knowledge and understanding of one’s illness, as well as hygiene practices and behaviours that prevent infections [6]. Accordingly, in a recent qualitative study with home healthcare nurses, informants reported that their patients’ knowledge of and attitudes towards infection prevention and engagement in hygiene practices influenced the patients’ behaviours regarding various infection control practices [7].

The practices of those providing home care, often nurse assistants and informal caregivers, have also been shown to influence the risk and spread of infection, especially in terms of good hand hygiene and use of sterile equipment [8]. Home healthcare staff have moreover reported on environmental factors such as clutter, poor lighting, uncleanliness, and pets as obstacles to infection control [6]. Such barriers may negatively affect patients’ infection risk through increased stress levels in healthcare professionals, which may in turn inhibit their adherence to adequate infection prevention practices [9, 10].

A systematic review from 2014 on the prevalence of infections and risk factors in home healthcare, showed that intravenous line-associated (catheter-related) infections (*n* = 19 out of 25 studies) were the most common types of infections [11]. In one of the included studies using a representative sample of Americans with home healthcare (*n* = 4,394; median age 74.6 years), the infection rate was 11.6% for all types of infections of which 3.6% were urinary tract infections (UTI) [12]. This makes infections in the home care setting a major concern, contributing to morbidity, mortality and considerable healthcare expenditures [13]. Further, in the review, the most common infection risk factors were patients’ underlying medical conditions, which was reported in 8 out of 14 studies that examined risk factors. Only one of all identified risk factors was non-medical (lower socio-economic status). Since the publication of the aforementioned systematic review in 2014, a substantial amount of research on infection risk factors has been conducted, of which some studies are referred to above. Several recent studies have examined risks for infection in the home setting beyond medical factors. These include behavioural or environmental factors, which constitute important aspects of infection risks that require due attention and consideration.

To add more knowledge to this subject-area, we conducted a systematic review investigating how facility characteristics, such as facility size, bed-capacity, the physical structure of the facility and staffing patterns, influence the spread of infectious diseases such as COVID-19 in care homes for older adults [14]. A total of sixteen studies were included. The findings suggested that larger facility size and urban location (compared to rural) may be associated with greater risks of an infectious disease outbreak. However, the risk of a larger outbreak seemed to be lower in larger facilities. Staff compartmentalizing seemed to be associated with lower risk of an outbreak whereas staff residing in highly infected areas appeared to be associated with greater risk of an outbreak. The systematic review on the spread of infectious disease in care homes triggered the development and planning of the present systematic review on risk factors for infection. In the current study, the home setting, rather than care homes, is of central focus. The home setting involves environmental challenges in terms of infection risks that are more difficult to overcome than in a care home. Several research studies published in the last few years have reported on multiple factors for the risk of infection in older adults with home care. However, the evidence from these studies has not been compiled and systematically reviewed in the past years, despite the pressing need for a review that synthesises and summarises existing knowledge using rigorous and transparent methods, generating implications for policy and practice.

With the present study, we seek to provide a deeper understanding of factors that are of particular importance for infection control in the home care setting. The importance of better protecting older adults with home care from infections and the potential consequences of hospitalisation and death became urgently apparent during the COVID-19 pandemic. The pandemic affected older adults and presented severe challenges in preventing spread of infection, specifically among older adults who depended on home care providers. This in turn highlighted the need for compiled evidence on factors that influence the risk of infections among older adults with home care.

Novelty of the present study includes the broad range of factors considered, including not only medical, but also individual, behavioural/social, and environmental factors. Additionally, contrary to the aforementioned systematic review by Shang and colleagues in 2014, the present systematic review includes qualitative research combined with quantitative studies and meta-analyses [11]. Another novelty of the current study is the inclusion of home help. The systematic review on infection risk from 2014 reported mainly on studies focusing on home healthcare [11]. Given that home help often involves close contact between caregiver and patient, infection risk is also highly relevant to home help, which in turn poses an increased risk for infection transmission [6]. Consequently, when investigating infection risk in older adults who live at home, it is essential to include home help in addition to home healthcare. In the present systematic review, home help refers to help received by professionals or informal carers to manage everyday life.

## Methods

### Study aim

This systematic review aims to identify risk factors for infection in older adults who receive home healthcare and/or home help. The structure of this review follows the guidelines for a mixed methods systematic review in the Joanna Briggs Institute (JBI) Manual for evidence synthesis [15].

### Review questions

The overarching review question is: What may be understood as risk factors for infection in older adults who receive home healthcare and/or home help?

For the quantitative evidence, the specific review question is: What factors are associated with diagnosis of infection and/or infection symptoms in older adults who receive home healthcare and/or home help?

For the qualitative evidence, the specific review question is: How do home care professionals and clients perceive infection risk factors and barriers to infection control and prevention?

### Inclusion criteria

To specify what data to be considered for the mixed methods systematic review, PICO (population, intervention, outcome, context) and additional criteria including phenomena of interest, context, and, type of study, was addressed and presented below.

#### Population

The review considered older adults who live in their own homes and receive healthcare and or home help by professionals or informal carers. Studies with populations of older adults living in long term institutions or who are hospitalized were excluded. Studies of populations receiving end-of-life care were excluded. We did not restrict the dose or duration of home help or home healthcare. Studies where the mean age of the sample was 65 years or above were considered eligible as 65 years of age often is used to define older adulthood [16, 17]. For some of the qualitative studies the age spans of the clients receiving home health care were not specified. Most of them, however, brought the theme of old age patients into the background and/or discussion sections of the article. We also knew that home health care clients, in their majority, tend to be over 65 years. Based on this information we opted for including the qualitative studies that fulfilled the rest of the PICO criteria.

#### Intervention/exposure

The review considered studies that assessed medical, individual, behavioural/social, environmental, and organisational factors for risk of infection.

#### Comparison

There were no restrictions for comparison groups of potentially eligible randomised control trials.

Studies with exclusive focus on the exposure to home parenteral nutrition or home infusion therapy were excluded. To our understanding, such studies in general included a sample of broader age range (sometimes even children). Also, even though these medical interventions take place at home, the primary focus of these studies cannot be considered as the practice of home healthcare delivery.

#### Outcomes

Studies with the following outcomes were considered: infection, infectious disease, communicable disease, and symptoms of infections (e.g. fever). Quantitative studies of adherence solely to infection prevention and control (IPC) protocols and practices were excluded as they reported on other outcomes.

#### Phenomena of interest

The qualitative component of this review considered studies that investigated the perceptions and understanding of infection risk as well as experiences of barriers to IPC practices in the home care setting. We considered studies containing client and home care staff perspectives, as well as studies of observations carried out during the delivery of home care. Studies with exclusive focus on home care agencies’ perspectives were excluded.

#### Context

The qualitative component of this review considered studies investigating the context of home help and/or home healthcare services provided by professionals in clients’ home setting.

#### Types of studies

The review considered quantitative (randomised trials and observational studies), qualitative, and mixed methods studies. Mixed methods studies were considered if data from the quantitative or qualitative components could be clearly extracted. There was no restriction in terms of publication date or language. Publications in foreign languages with no title or abstract in English or Swedish, yet potentially eligible, were translated.

Studies with no original data e.g., reviews and opinion pieces were excluded.

### Methods approach

The systematic review has applied a convergent segregated approach as defined by the JBI Manual for evidence synthesis. The Preferred Reporting Items for Systematic Reviews and Meta-Analyses (PRISMA) guidelines have been used (Appendix 1). The study was prospectively registered in PROSPERO (CRD42021261159) and the study protocol has been published [18].

### Search strategy

Search terms were developed in consultation with two university librarians at Karolinska Institutet in Stockholm, Sweden. A test search was carried out using the following databases: MEDLINE, Embase, Web of Science, CINAHL, and Sociological Abstracts. This generated 5,841 articles of which the 300 most recently published titles were screened by two researchers (AL, MB). The researchers identified about 30 relevant articles each, of which most overlapped. The articles were read in full and discussed with the librarians who refined the search strategy.

The search was carried out on 20th May 2022, using the search terms outlined in Appendix 2 and referred to home care, home healthcare and home help in various combinations with infection, infectious diseases and communicable diseases. In addition, reference lists of a previous systematic review [11] and the studies selected for critical appraisal were screened for additional studies. An updated search was carried out on 22nd February 2023, to include articles published since the initial search (Appendix 3).

### Information sources

Databases searched were MEDLINE (Ovid), Embase, Web of Science (Clarivate) Sociological Abstracts (ProQuest) and CINAHL (EBSCO).

### Study selection

Following the search, all identified records were collated and uploaded into Rayyan (rayyan.ai) and duplicates were removed. Titles and abstracts of the remaining records were then screened by two reviewers (MB, GM) for assessment against the inclusion criteria for the review. One doctoral thesis occurred in the searches and was substituted by related scientific papers by the same author. Studies that met the inclusion criteria were retrieved and their full texts were assessed in detail against the inclusion criteria by the two independent reviewers. Librarians assisted with the retrieving of articles not available online. Articles written in languages not spoken by the researchers were translated into English. Full text studies that did not meet the inclusion criteria were excluded and reasons for exclusion are provided in Appendix 4. Any disagreement that arose between the reviewers were resolved through discussion with a third researcher (AL). The same process was repeated for the second search. The titles generated in the second search were screened by researchers EG and AN, and discussed with AL.

### Assessment of methodological quality

#### Qualitative process

Eligible studies were critically appraised by two independent reviewers (MB, AL) for methodological quality using the Critical Appraisal Skills Programme tool (CASP) (casp-uk.net). The tool was slightly adapted, changing Q10 “How valuable is the research?” into “Was the research valuable?”, to allow for a three-level scoring system composed of “Yes”, “To some extent” and “No” for all questions. Any disagreements that arose between the reviewers were resolved through discussion. All qualitative articles that received one or more “No” ratings were further discussed, and it was decided that they should be excluded. The same procedure was repeated by researchers EG and AN for the qualitative studies identified in the updated search. Authors of eligible studies with unsupported findings were contacted. Upon request, authors of one of the eligible studies provided supplementary data files that were inaccessible through the web links referred to in the journal. Authors of some of the other eligible qualitative studies not providing quotations to support their findings were also contacted but they did not reply.

#### Quantitative process

Eligible quantitative studies were critically appraised, and their quality systematically assessed by reviewer CB and cross-checked by AL. Any disagreement that arose between the reviewers was resolved through discussion. The critical appraisal checklist tools for systematic reviews of aetiology and risk, available in JBI Manual for Evidence Synthesis, were used. Different critical appraisal checklists were used according to the study design of each quantitative study, with separate checklists for cohort studies and cross-sectional studies [19, 20]. No adaptations of the checklist tools were made, but where these were unclear in their questions, an agreed meaning was found between the two reviewers.

### Data extraction

Quantitative and qualitative data were extracted from included studies using relevant JBI data extraction tools [15]. Only data that were relevant to the outcomes of this study were extracted.

For qualitative studies, data extracted included specific details about the aim, geographical setting, methods, participants, context, and the phenomenon of interest relevant to the review question. Findings with the studies’ corresponding illustrations were also extracted from the analytic text in the results sections. Relevant contradictory findings with regards to IPC practices were also extracted and included. All findings were assigned a level of credibility: “Unequivocal”, “Credible” or “Not supported”, in accordance with the ConQual approach [21].

For quantitative studies, data extracted included details about the study method/characteristics, the outcome of significance to the review, the data analysis methods including statistical technique, and the study results. This process followed the data extraction for Systematic reviews of aetiology and risk available in JBI Manual for Evidence Synthesis [15].

### Meta-analysis of quantitative findings

Meta-analyses were carried out to determine an overall trend and conducted when the same risk factor was reported in three or more studies. Multiple meta-analyses were performed because of the heterogeneity in outcomes and risk factors across the studies, which did not allow for one overall meta-analysis. The meta-analyses were undertaken by combining study-specific odds ratios (ORs) and confidence intervals (CIs) using the DerSimonian and Laird’s random-effects model. Statistical analyses were performed using the STATA software (version 16.1; StataCorp LLC, College Station, TX, USA). The findings of the meta-analyses are presented as forest plots.

### Data synthesis and integration

A convergent segregated approach to synthesis and integration was applied.

The qualitative synthesis of findings was carried out using a meta-aggregative approach. Only findings assessed as “Unequivocal” or “Credible” were included in the synthesis. Findings were grouped into categories based on similarity of meaning. Categories contained at least two findings and descriptions were created by consensus process between reviewers. Synthesised findings, containing two or more categories, were created based on similarity of meaning, in a consensus process between reviewers.

The quantitative synthesis of findings was presented in narrative form as suggested by JBI. Where possible, a statistical pooling of recurrent findings was conducted, resulting in meta-analyses to estimate a summary average effect of a specific risk factor.

The synthesised findings based on the quantitative and qualitative evidence were integrated using a configuration approach, whereby the evidence from each separate part was compared and linked to produce one line of argument [15]. Where such configuration was not possible, integration of findings was instead presented in a narrative form.

### Methodological quality

#### Critical assessment of qualitative studies

Table 1 shows the rating of the sixteen eligible qualitative studies assessed for methodological quality using an adapted version of CASP providing 10 questions with answer options *Yes*, *To some extent*, and *No*. CASP was chosen due to its pragmatic approach and assessment of coherence between aim and methods including data collection and analysis choices, and transparency in the reporting of the choices made.

**Table 1:** Critical appraisal of eligible qualitative studies

Citation	Q1	Q2	Q3	Q4	Q5	Q6	Q7	Q8	Q9	Q10
Dowding et al., 2022	Y	Y	TSE	TSE	TSE	TSE	Y	TSE	TSE	Y
Wendt et al., 2022 [44]	Y	Y	Y	TSE	Y	TSE	TSE	Y	Y	Y
Russell et al., 2021 [6]	Y	Y	TSE	TSE	TSE	N	TSE	N	TSE	Y
Pogorzelska-Maziarz et al., 2021	Y	Y	TSE	TSE	TSE	TSE	TSE	TSE	TSE	Y
Bandini et al., 2021 [23]	Y	Y	Y	TSE	TSE	N	TSE	N	TSE	TSE
Bell et al., 2021	Y	Y	Y	Y	Y	TSE	TSE	TSE	TSE	Y
Felembam et al., 2015	Y	Y	Y	TSE	TSE	N	N	N	N	N
Osakwe et al., 2021 [45]	Y	Y	Y	TSE	Y	TSE	Y	TSE	Y	Y
Sterling et al., 2020 [46]	Y	Y	TSE	Y	Y	TSE	TSE	TSE	TSE	Y
Franzosa et al., 2022 [51]	Y	Y	Y	Y	Y	TSE	Y	TSE	Y	Y
Tavemark et al., 2022 [47]	Y	Y	Y	Y	TSE	N	Y	Y	Y	Y
Emmesjö et al., 2022 [48]	Y	Y	Y	Y	TSE	N	TSE	TSE	Y	Y
Baumbusch et al., 2022 [50]	Y	Y	Y	Y	Y	N	Y	TSE	Y	Y
Moi et al., 2022 [49]	Y	Y	Y	TSE	TSE	N	Y	TSE	Y	Y
Rezende et al., 2022 [25]	Y	Y	TSE	Y	Y	N	TSE	N	N	TSE
Prout et al., 2022 [26]	Y	Y	TSE	Y	Y	N	TSE	N	TSE	TSE

Adapted version of CASP

All questions were answered by one of the following ratings: “Yes”, “To some extent” (TSE), or “No”

Q1: Was there a clear statement of the aims of the research?

Q2: Is a qualitative methodology appropriate?

Q3: Was the research design appropriate to address the aims of the research?

Q4: Was the recruitment strategy appropriate to the aims of the research?

Q5: Was the data collected in a way that addressed the research issue?

Q6: Has the relationship between researcher and participants been adequately considered?

Q7: Have ethical issues been taken into consideration?

Q8: Was the data analysis sufficiently rigorous?

Q9: Is there a clear statement of findings?

Q10: Is the research valuable?

Five studies [6, 22–25] received more than one *No* rating, indicating poor trustworthiness, and were therefore excluded following discussion. Details of the excluded studies can be found in Appendix 5.

#### Critical assessment of quantitative studies

The eighteen eligible quantitative studies were assessed for methodological quality using the critical appraisal checklist tools for Systematic reviews of aetiology and risk, available in JBI Manual for Evidence Synthesis [19, 20]. The checklists refer to certain study designs. The critical appraisal checklist tool for cohort studies was used for the six cohort studies [26–31], and the critical appraisal checklist tool was used for analytical cross-sectional studies for the remaining 12 studies [3, 5, 30, 32–40].

##### Cohort studies

The checklist tool for cohort studies consisted of 11 questions (Table 2). Each question was rated *Yes*, *Unclear*, *No*, or *Not Applicable*. No study was excluded due to methodological quality concerns. The potential influence of the studies with one or more Unclear or No ratings on the outcomes of the meta-analyses and overall conclusions, were discussed.

**Table 2:** Critical appraisal of eligible cohort studies

Citation	Q1	Q2	Q3	Q4	Q5	Q6	Q7	Q8	Q9	Q10	Q11
Noguchi et al., 2020 [27]	Y	Y	U	Y	Y	Y	U	Y	U	Y	Y
Osakwe et al., 2019 [28]	Y	Y	Y	Y	Y	N	Y	Y	N	N	Y
Shih et al., 2019 [29]	Y	Y	Y	Y	Y	U	Y	Y	U	N	Y
Yokobayashi et al., 2013 [30]	Y	Y	Y	Y	Y	U	Y	Y	Y	Y	Y
Yokobayashi et al., 2014 [32]	Y	Y	Y	Y	Y	U	U	Y	Y	Y	Y
White et al., 1995 [52]	Y	Y	Y	Y	Y	N	Y	Y	U	Y	Y

*Y* yes, *N* no, *U* unclear

JBI Critical appraisal checklist for cohort studies

Q1: Were the two groups similar and recruited from the same population?

Q2: Were the exposures measured similarly to assign people to both exposed and unexposed groups?

Q3: Was the exposure measured in a valid and reliable way?

Q4: Were confounding factors identified?

Q5: Were strategies to deal with confounding factors stated?

Q6: Were the groups/participants free of the outcome at the start of the study (or at the moment of exposure)?

Q7: Were the outcomes measured in a valid and reliable way?

Q8: Was the follow up time reported and sufficient to be long enough for outcomes to occur?

Q9: Was follow up complete, and if not, were the reasons to loss to follow up described and explored?

Q10: Were strategies to address incomplete follow up utilized?

Q11: Was appropriate statistical analysis used?

##### Cross-sectional studies

The checklist tool for cohort studies consisted of eight questions rated *Yes*, *Unclear*, *No*, or *Not Applicable* (Table 3). One study received two *No* and three *Unclear* ratings, and another study received three *No* and three *Unclear*, indicating poor quality. For this reason, these two studies were excluded following discussion. Details of the excluded studies are outlined in Appendix 5.

**Table 3:** Critical appraisal of eligible cross-sectional studies ^1^

Citation	Q1	Q2	Q3	Q4	Q5	Q6	Q7	Q8
Barros et al., 2018 [32]	Y	Y	Y	Y	N	N	Y	Y
Chikanya et al., 2003	U	Y	U	N	N	N	U	Y
Harrison et al., 2022 [34]	Y	Y	Y	Y	Y	Y	Y	Y
Lin et al., 2020 [35]	Y	Y	Y	Y	U	U	Y	Y
Marrie et al., 2005 [40]	Y	Y	U	Y	N	N	Y	Y
Morioka et al., 2021 [36]	U	U	Y	U	Y	Y	U	Y
Pärn et al., 2016 [37]	Y	U	Y	Y	U	U	Y	Y
Rönneikkö et al., 2018 [38]	Y	U	Y	Y	Y	Y	Y	Y
Shang et al., 2020a [3]	Y	Y	Y	Y	N	N	Y	Y
Shang et al., 2020b [5]	Y	Y	Y	Y	U	U	Y	Y
Shang et al., 2022 [39]	Y	Y	Y	Y	Y	Y	Y	Y
White et al., 1992 [30]	U	U	U	Y	N	N	Y	Y

*Y* yes, *N* no, *U* unclear

JBI Critical appraisal checklist for analytical cross-sectional studies

Q1: Were the criteria for inclusion in the sample clearly defined?

Q2: Were the study subjects and the setting described in detail?

Q3: Was the exposure measured in a valid and reliable way?

Q4: Were objective, standard criteria used for measurement of the condition?

Q5: Were confounding factors identified?

Q6: Were strategies to deal with confounding factors stated?

Q7: Were the outcomes measured in a valid and reliable way?

Q8: Was appropriate statistical analysis used?

## Results

### Study inclusion

The Prisma flowchart is presented in Appendix 3. The initial search generated 17,984 records of which 80 studies were read in full text. The hand search of reference lists resulted in the identification of one additional eligible study. Excluded studies read in full text are listed in Appendix 4. A total of 17 quantitative studies and nine qualitative studies were critically appraised for methodological quality. This appraisal resulted in the exclusion of one quantitative and three qualitative studies (Appendix 5).

The updated search generated 1,500 records of which 69 were read in full. Sixty-one of these studies were excluded (Appendix 4). A total of seven qualitative studies and one quantitative study were critically appraised for methodological quality, which resulted in the exclusion of the quantitative study and two qualitative studies (Appendix 5).

Thus, out of 19,484 studies a total of 27 studies (16 quantitative studies and 11 qualitative studies) were included in the review.

### Characteristics of included studies

Overviews of the characteristics of the included studies are presented in Appendix 6 (qualitative studies), Appendix 7 (cohort studies) and Appendix 8 (cross-sectional studies).

#### Qualitative studies

##### Setting and participants

Most qualitative studies explored infection prevention strategies and practices [7, 41–43], and perceived barriers and facilitators to IPC [42–45]. Whilst one study specifically examined the agency perspective [42], the remaining studies focused on the delivery of care in clients’ homes. All studies included interviews with home care staff, who were predominantly female. One study also included observations and focus groups [43]. Eight of the studies focused on home care during the Covid-19 pandemic [41, 44–50]. The studies were published between 2020 to 2022 and most of them were undertaken in Western societies; half of the studies had been conducted in the United States (U.S.).

#### Quantitative studies

##### Setting and participants

Five of the 16 quantitative studies used data from databases or registers [3, 5, 27, 36, 38]. The sample sizes ranged from 81 [43] to 156,408 patients [39]. Most of the studies reported the participants’ mean age, which ranged between 63 to 85 years (with the exception of two studies [26, 32]). The studies were published between 1995 and 2022. Most studies were conducted in Western societies including five from the U.S. [3, 5, 27, 34, 39].

##### Study-specific outcomes and tools

Among the sixteen quantitative studies, the most recurrent infections were urological-related of which urinary tract infection (UTI) was the most common infection [3, 5, 26–29, 34, 37, 38, 51], followed by respiratory-related [3, 5, 26, 29, 31, 32, 35, 38–40], and skin-related infections [3, 5, 26, 29, 38]. Two studies had fever events as the main outcome [29, 31] and three studies focused on IPC protocols and practises [34, 36, 39]. Five studies did not focus on a specific infectious disease [3, 26, 36, 38, 39].

The included studies used different sources as exposures. The Outcome and Assessment Information Set (OASIS) was used as source of data collection or secondary analysis in three of the American studies [3, 5, 34]. The Finnish studies used a similar tool called Resident Assessment Instrument (RAI) [37, 38]. The OASIS and RAI are pre-defined standardised assessment tools administered to home care patients (for USA) [52], or patients receiving long term care (in Finland) [53], at admission and repeated when necessary. Data from these tools were combined with national registers [37], hospital and national discharge records [38] or other databases [34] for the addition of complementary data on patients’ characteristics, diagnoses, or agency information relevant to the study. One study used data from specific home care agency databases [28] while three studies were based on self-administered survey data [26, 32, 36]. Two studies measured exposure through direct recordings by the study participants [29, 31]. Other sources of data were clinical records [32, 35, 51], outpatient department records, home visit records, discharge charts [35] and laboratory data [28, 35].

For outcome data, medical records were the most frequent data source [29, 32, 35], where diagnoses were made by qualified physicians with [28, 34, 35, 40] or without [32] referring to the International Classification of Diseases (ICD) codes. Results from laboratory tests were used to support the infection diagnosis in two studies [32, 35] whereas two studies used self-reported or medically diagnosed data on infections regardless of laboratory tests [29, 36]. The remaining studies did not mention laboratory test results. For some studies, the outcome was measured using self-administered surveys completed by a qualified medical professional [26, 31, 36] or by the patients themselves [26].

### Findings of the review

The findings present quantitative and qualitative data separately.

#### Quantitative evidence

Risk factors reported in the quantitative studies referred to individual, medical, social, behavioural, environmental and organisational factors.

##### Individual

Female sex [5, 27, 37], older age [5] and being of white ethnicity [5, 27] were found to be factors significantly associated with greater odds of UTI and UTI-related hospitalizations in home care patients. Male sex [5, 32, 35], older age [5, 32, 40] and white ethnicity [5] were found to have a significant association with the development of respiratory infections and related hospitalization. Younger age and male sex were found to be individual risk factors related to significantly higher odds of developing a wound-site infection [5]. Studies that did not focus on any specific infections, found that infection-related outcomes were more likely to occur in individuals of male sex, white ethnicity and older age [3]. Older age was also associated with fever events considered a symptom of infection [31].

##### Medical

The majority of the medical risk factors were specific infectious-related and associated to medical conditions, practices, or devices. Among the included studies, the use and management of urinary catheter was found to be the major risk factor for UTI in home care patients. Presence of a urinary catheter was found statistically associated with UTI events and related hospitalizations in two cohort studies [27, 28] and two cross-sectional [5, 40]. A significant association was also found between time to UTI and frequency of intervals in catheter change, the number of nurses changing the catheter [51], history of urinary catheter use, and history of UTI treatment prospectively [27] and cross-sectionally [5, 37]. Besides, urinary incontinence was found to be associated with greater odds of developing UTI in three cross-sectional studies [5, 37, 38] yet shown to be a protective factor in a retrospective cohort study [37].

Hospitalization due to UTI was associated with the presence of comorbidities [5, 28, 37], hyper-polypharmacy [28], cognitive dysfunction [5, 37], severe difficulties in undertaking basic and instrumental activities of daily living (ADLs) [3, 27, 37], being chair fast [5], and having a history of hospitalization [5, 37]. Based on a retrospective cohort study, UTI was one of the most common diagnose causing fever events [29]. In one retrospective cohort study [27] a few medical factors were associated with a decrease in the odds of infection: a mild ADL dependence, prior stay in an acute care hospital, and presence of urinary incontinence.

Nasogastric tube use [35], recurrent aspiration [40] and receiving respiratory treatments at home [5, 40] were associated with greater odds of having a respiratory infection. The presence of chronic respiratory diseases [35, 40] or comorbidities [5, 40] were also found to be associated with respiratory infection.

Wound infections and decubitus ulcers were the most common skin-related infections reported in the included studies. Skin related medical conditions were found to be significantly associated with an increased risk of wound infections or related hospitalisation. In a cross-sectional study [5], having a stasis ulcer, surgical wounds, skin lesions or open wounds, or severe unhealed pressure ulcers, was associated with developing a wound-site infection. Moreover, limited mobility, progressive conditions, multiple hospitalisations, difficulties managing injectable medications and needing help with changing wound dressing were also associated with infection risk [5]. Fever events were commonly associated with skin and soft tissue infections [29].

Two cross-sectional [3, 38] and one prospective cohort study [26] studied underlying causes of infection-related hospitalizations and risk factors for infectious-related outcomes in home healthcare patients. In their cross-sectional study [3], home healthcare was associated with greater risks of receiving emergency care due to infectious diseases (respiratory, wound, urinary tract, and intravenous catheter-related infections). The other cross-sectional study found that functional and cognitive impairment were associated with higher odds of being hospitalised with an infectious disease diagnosis, along with chronic skin ulcers and daily urinary incontinence [38].

Medically related factors associated with infectious disease were Parkinson’s disease, dermatosis not caused by pressure ulcers, chronic respiratory failure and inability to perform oral self-care [26]. Two studies that assessed fever events and their risk factors found associations with limited mobility, needing a respiratory device, presence of comorbidities, having cognitive impairment [31] or having comprehensive care needs [29, 31].

##### Social

Among the included studies, the social factors associated with risk of infections in the older population with home care were mainly related to the availability and help from caregivers. Patients hospitalised due to infections were generally more likely to need help with ambulation with management of medical equipment [3], and with meal preparation [5] as well as need of supervision by a caregiver for their safety [27]. The presence of a caregiver to assist with ADLs was associated with lower odds of being hospitalised for UTI in one retrospective cohort study [27], while having no caregivers to assist was associated with more than doubled odds of being hospitalised for an infection in two cross-sectional studies [3, 5]. Being eligible for Medicaid insurance was found to be associated with hospitalisation for UTI [27] and other infections [3]. Given that Medicaid is offered to people below the federal poverty level [27], Osakwe and colleagues used this information as proxy for socio-economic status.

##### Behavioural

Smoking was the personal behaviour found to be statistically associated with increased odds of hospitalisation due to respiratory infections [5]. Memory deficit, defined as neuro/emotional/behavioural status, was associated with infection-related outcomes [3].

##### Environmental

Home care patients’ living situation was the most common environmental factor assessed in the included studies. Living in a shared accommodation or congregate setting were found to be associated with increased odds of developing a respiratory infection [5, 32], UTI or wound infection [5]. One study also found increased odds of developing acute lower respiratory infection among patients who reported a different waste collecting system from the public system [32].

##### Organisational

Three cross-sectional studies [34, 36, 39] specifically examined IPC policies and practices. Harrison et al. showed that policies on replacement of indwelling catheters at fixed intervals, and policies on emptying the drainage bag, lowered the probability of hospital transfer due to UTI in the home care population [34]. Shang and colleagues examined predicted rates of hospital transfers due to respiratory infections among home care patients in different agencies and found that influenza vaccination requirements for home care staff would reduce hospitalizations among patients in for-profit home care agencies, but not in non-profit agencies. If all home care agencies adopted influenza vaccination requirements for staff (called *Scenario 2*), there would be a 15.75% reduction in the rate of hospital transfer due to respiratory infection in for-profit agencies, compared with the status quo (*Scenario 1*) (*P* < 0.05) [39]. The third cross-sectional study found that the presence of a committee for IPC practices and providing staff training on infection prevention were associated with increased incidence of reported infections in agencies with such policies and practices in place [36]. Finally, having a caregiver in need of additional training on adherence to IPC practices was found to be associated with significantly higher risk of developing infections among home health patients, as evidenced by two cross-sectional studies [3, 5].

#### Meta-analyses

The results from the meta-analyses conducted based on studies that individuated the same risk factor are reported below. These risk factors were urinary incontinence, urinary catheter use and limited mobility. Outcomes were infection development of UTI or urinary incontinence.

##### Urinary incontinence and development of UTI (or other infections)

**Fig. 1:**
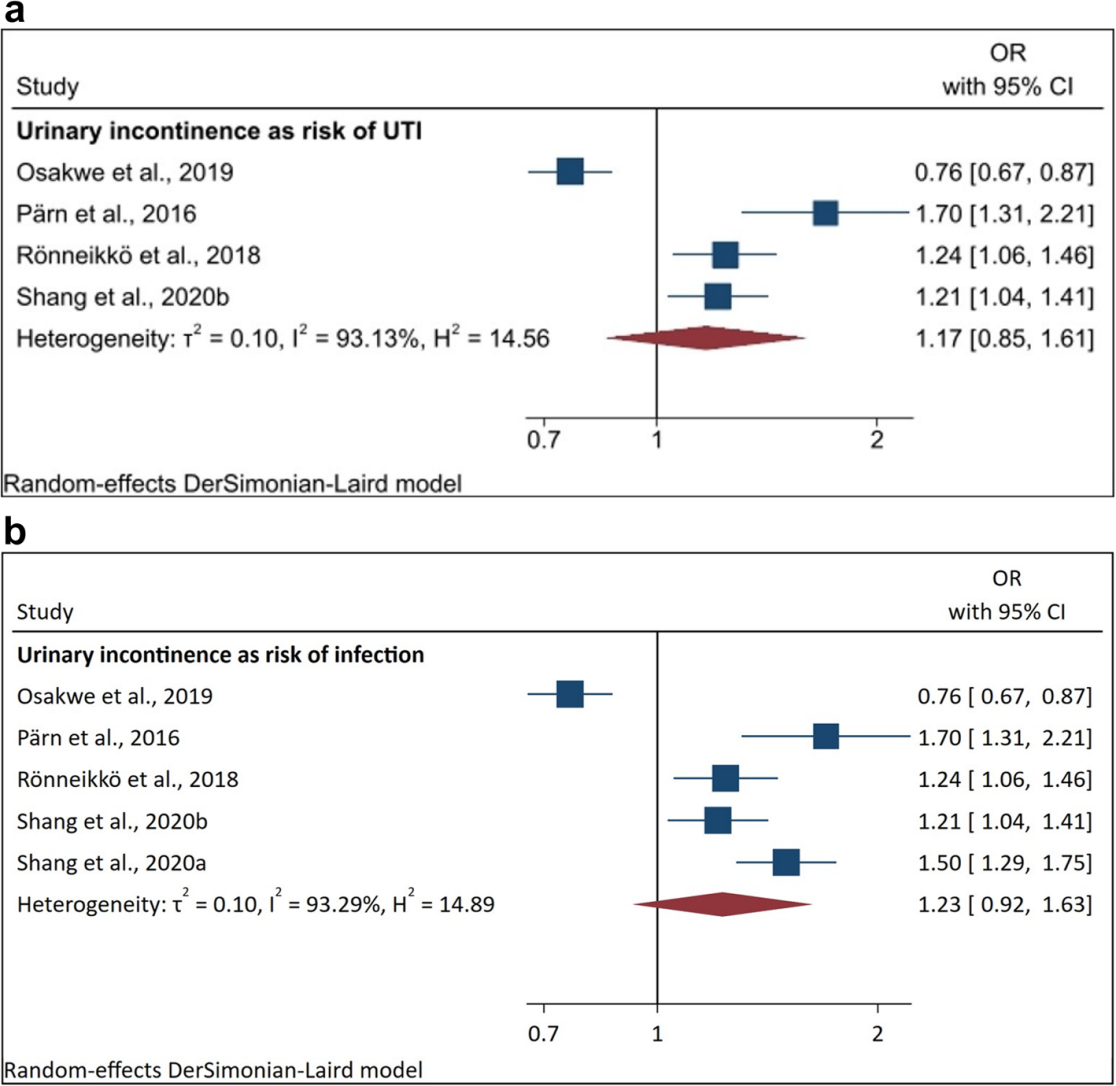
**a** Pooled ORs of urinary incontinence on the development of UTI. **b** Pooled ORs of urinary incontinence on the development of infection

Four studies individuated urinary incontinence as a factor related to UTI [5, 27, 37, 38]. The computation of the meta-analysis on urinary incontinence as risk of UTI showed odds ratio (OR) of 1.17 (95%CI [0.85–1.61]) and 93.13% heterogeneity (Fig. 1a).

When adding research not restricted to UTI-infections [3], the OR changed to 1.23 (95%CI [0.92–1.63]), and heterogeneity to 93.29% (Fig. 1b).

##### Urinary catheter use and development of infection outcomes

**Fig. 2:**
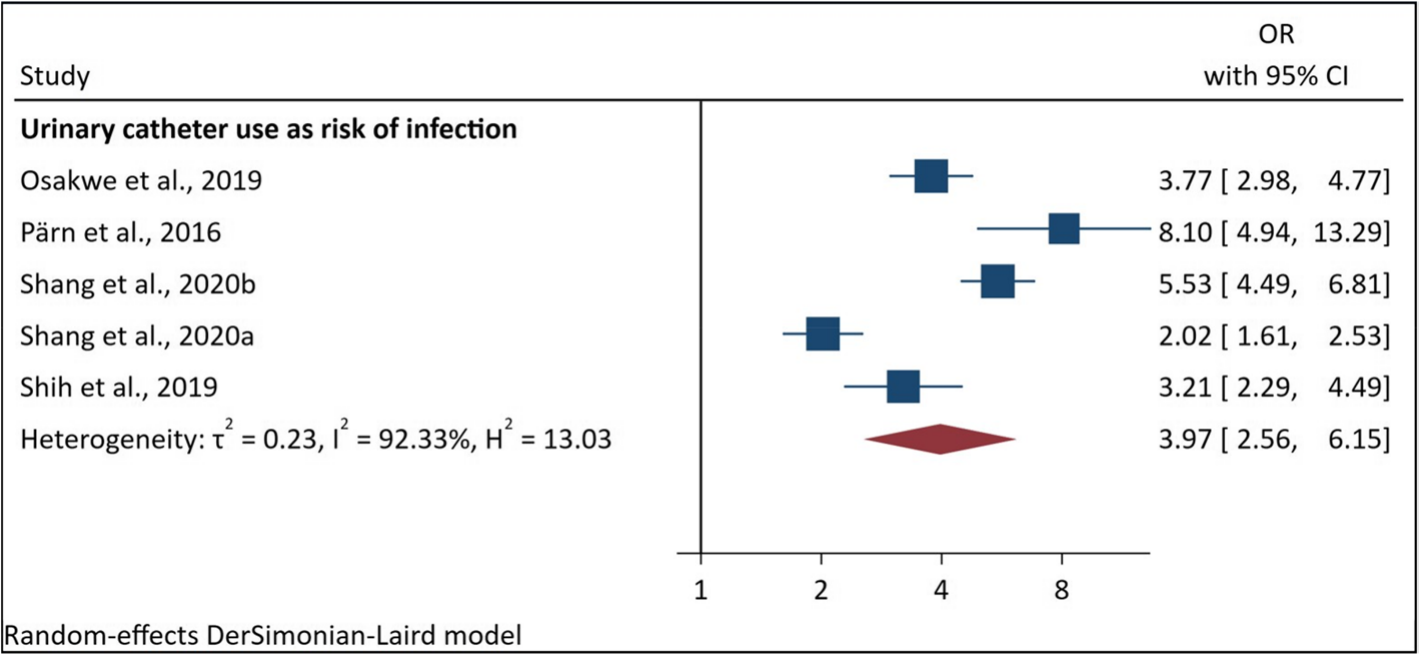
Pooled ORs of urinary catheter use and risk of infection

Urinary catheter use was a recurrent factor associated with infection outcomes. The meta-analysis pooling the ORs of these studies resulted in an overall OR of 3.97 (95%CI [2.56–6.15], $\tau$ ^2^ = 0.23) with 92.33% heterogeneity (Fig. 2).

##### Limited mobility and risk of infection

**Fig. 3:**
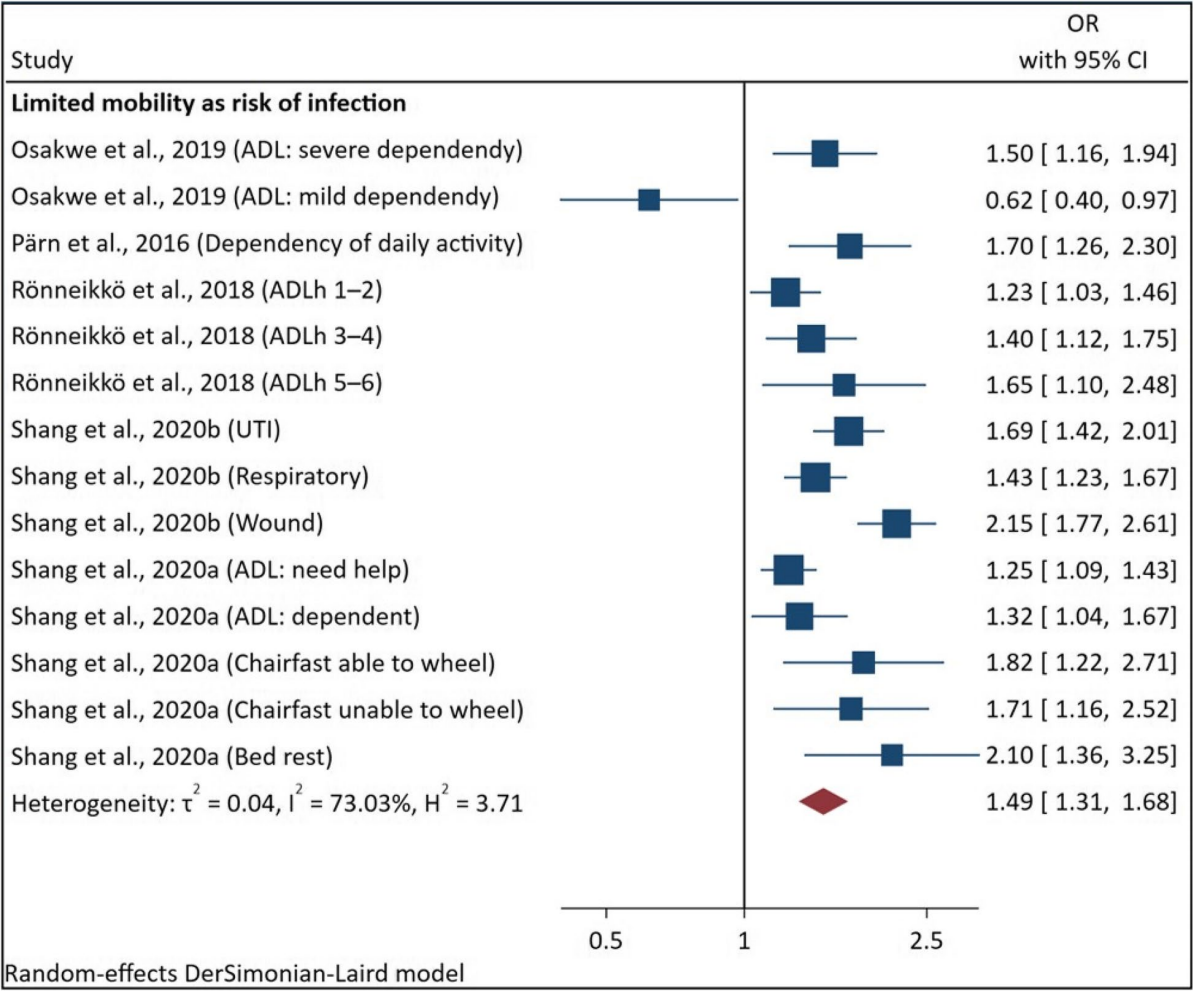
Pooled analysis of ORs of limited mobility and risk of infection

Limited mobility and risk of infections resulted in an overall estimated OR of 1.49 (95%CI [1.31–1.68]) with 73.03% heterogeneity (Fig. 3).

#### Qualitative evidence: meta-aggregation

In this section, older adults are referred to as clients as this word was commonly used in the included qualitative studies.

A total of 125 findings from eleven qualitative studies were extracted and grouped into 17 categories based on similarity of meaning. There were 85 unequivocal findings, 26 credible findings, and 14 unsupported findings. The findings and illustrations related to each study can be found in Appendix 9. The categories were aggregated into five synthesised findings presented below. Unsupported findings were not used in the meta-aggregation.

**Synthesised finding 1—Clients'characteristics, health state and practices determine their exposure and susceptibility to risk factors of infections. Client attitudes and behaviours are central to IPC (**Table 4**).**

**Table 4:** Synthesised finding 1: Clients'characteristics, health state and practices determine their exposure and susceptibility to risk factors of infections. Client attitudes and behaviors are central to infection prevention and control

**Category 1: Risk factors related to specific infections, health conditions and client characteristics**
Nurses identified several factors that they felt put patients at a higher risk of infection. Key indicators included having a history of infections or a prescription for an antibiotic. (Dowding 2020) (U)
Wounds were a key infection risk and their assessment and management are a large part of the nurses’ work. (Dowding 2020) (U)
Having particular points of entry for infection, such as intravenous (IV) lines, Foley catheters and wounds were identified as factors that automatically put a patient at a higher risk of infection. (Dowding 2020) (U)
A diagnosis of chronic obstructive pulmonary disease (COPD), and a history of smoking, were specific factors related to a risk of respiratory infections such as pneumonia (Q12). (Dowding 2020) (U)
Overall, there has to be an underlying reason why an individual is at risk of infection. (Dowding 2020) (U)
**Category 2: Infection as a result of multiple health issues and interaction with environment**
Risk of infection was the result of a combination of factors that affected a patient’s immune system such as older age, diabetes, dehydration or having inadequate nutrition. (Dowding 2020) (U)
Patients who also had a diagnosis of diabetes were at particular risk and nurses discussed in detail their process of wound assessment to ensure they detected any infections quickly. (Dowding 2020) (U)
The risks for urinary tract infection (UTI) were complex with factors such as cognition, incontinence, availability of caregivers and environmental conditions all having a role to play. (Dowding 2020) (U)
**Category 3: Clients'lack of understanding of infection risk and low adherence to infection prevention and control practices pose risk of infections**
Nurses regarded patients’ knowledge and understanding of their illness and factors associated with infection risk and infection prevention behaviors as being closely linked to their risk of infection. This included issues such as culture and beliefs, personal hygiene practices and their understanding of how infections were spread and could be prevented. Overall nurses felt their patients lacked basic knowledge about infection prevention, including basic health and hand hygiene, or held beliefs and attitudes towards infection control that nurses perceived were not at a “high standard”. (Dowding 2020) (U)
Patients’ behaviors, in relation to general health promotion such as getting vaccinations, impacted on their infection risk. (Dowding 2020) (U)
Adhering to treatment recommendations, such as following medication regimens (e.g. courses of antibiotics), managing their wounds, diabetes management and hand washing were important considerations for assessing infection risk. (Dowding 2020) (U)
Nurses faced particular challenges when talking to a patient about cleaning up their home to avoid infection, and often patients did not have a suitable family caregiver to support the nurse’s attempts at reducing infection risk. (Dowding 2020) (U)
Both patients and caregivers may show a resistance to change, may have low health literacy, and may be cognitively impaired. (Dowding 2020) (C)
Mental health –especially stress –also presented a challenge to patient and caregiver education. (Dowding 2020) (C)
Home health aides (HHAs) also feared that their vulnerability to COVID-19 was greater because patients sometimes did not adhere to wearing masks. (Osakwe 2021) (C)
When patients asked for more closeness, the nurses experienced this to be difficult. (Moi 2022) (U)
A new role for caregivers was supporting their relative to follow public health measures. Wearing masks and handwashing became part of everyday life during the pandemic but supporting a person living with dementia to follow these guidelines could be challenging. (Baumbusch 2022) (U)
Changing lifelong social practices was a difficult aspect of public health measures, as was building mask wearing into their daily routines. (Baumbusch 2022) (C)

*C* credible, *U* unequivocal

The individual level is represented in this synthesised finding, where the home care client forms the main focus of infection risk factors. It is made up of three categories, containing 15 unequivocal findings and four credible findings from four studies [7, 44, 49, 50].

**Category 1. Risk factors related to specific infections, health conditions and client characteristics.** Clients are believed to be at risk of infection depending on their history of infections, diagnosis and current health state, such as having a catheter. Each infection is observed to be related to specific risk factors, as illustrated below:

*“Because somebody with COPD (chronic obstructive pulmonary disease) is obviously at higher risk of developing a lung infection, right? But not necessarily wound infection. So it depends what type of infection you're talking about. Certain people are more at risk for developing respiratory infection because of smoking and I think smoking always puts you at higher risk for infection, in general.”* [7] (Appendix 9, Q5).

**Category 2. Infection as a result of multiple health issues and interaction with environment.** Infections are considered to be a result of several risk factors affecting a client's health state, *“such as, older age, diabetes, dehydration or having inadequate* nutrition”. [7] (Appendix 9, Q6 & Q9).

Furthermore, infections are understood to be caused by interacting risk factors on the individual level, in the home environment and the reception of care. *“So a lot of times elderly people that are incontinent, they tend to get urinary tract infections pretty easily. So probably an older person who's incontinent, who's in bed, who maybe doesn't have a lot of help.”* [7] (Appendix 9, Q8 & Q9).

**Category 3. Clients'lack of understanding of infection risk and low adherence to infection prevention and control practices pose risk of infections.** Clients'knowledge, attitudes and behaviour largely influence the risk of infection in home care where the staff has less control over the delivery of care and care environment. Clients'adherence to treatments, hygiene and health practices, as well as attitudes and capacity to maintain a clean environment are all believed to be key aspects of IPC. Illustrations included the following:

*“Then you’re looking at their attitude towards what you’re talking about when you ask them to wash their hands. […] Sometimes they think that one time is enough, and that’s it. And then how receptive they are to you just teaching them. […] Sometimes they’ll say, ‘Uhhuh,’ but you know that it’s just going in one ear, then going out the other.”* [7] (Appendix 9, Q29).

*"The biggest issue is trying to get him to remember to wear his mask and not to hug people or shake their hands? He’s just got his hand out there”* [49] (Appendix 9, p.187).

**Synthesised finding 2. The state of the home environment, the interactions between the client and other members of the household, as well as caretakers'participation in healthcare delivery may all represent risk factors for infection. In the home environment the home care provider exercises limited influence over IPC (**Table 5**).**

**Table 5:** Synthesised finding 2: The state of the home environment, the interactions between the client and other members of the household, as well as caretakers'participation in health care delivery may all represent risk factors for infection. In the home environment the home health care provider exercises limited influence over infection prevention and control

**Category 4:** **Exposure to environmental risks in the home**
If patients lived in an environment that was unclean, then this increased the risk of a patient getting an infection. (Dowding 2020) (U)
Nurses reported patients living in apartments without running water, or a working sink. (Dowding 2020) (U)
The nursing care environment varied from impeccably clean and tidy –without visible dirt, dust or damaged interiors –to dilapidated and contaminated households. The latter were littered with putrid waste, sticky floors, damaged interiors and inadequate lighting and lacked fresh air or adequate space for movement. (Wendt 2022) (U)
In some cases, there was not enough of a free flat surface to create a clean workspace. (Wendt 2022) (U)
There may be evidence of hoarding or the absence of adequate indoor plumbing, which can further complicate healthcare provision in the home. (Pogorzelska-Maziarz 2020) (C)
**Category 5:** **Exposure to frequent personal interactions and unsafe caregiving by family members **
When patients lived with many family members in close proximity and their infection control practices were not thought to be of a high standard, this was also seen as a risk factor. (Dowding 2020) (U)
Besides the client and nurse, other people, […] could be present during the delivery of care, varying from none to many; for example, healthcare staff, relatives, acquaintances, children, domestic help. (Wendt 2022) (U)
Some staff described difficulties with ensuring patient, family, and caregiver compliance with treatments and IPC procedures because home health staff are not present in the home all of the time. (Pogorzelska-Maziarz 2020) (C)
Patients who had caregivers or family that were not following advice or being involved in care were seen to be at an increased risk of developing infection. (Dowding 2020) (U)
Noncompliance was viewed as a major challenge; many participants gave detailed accounts of the lengths they went to teach patients and their families and caregivers about wound care, environmental sanitization, and medication adherence. (Pogorzelska-Maziarz 2020) (C)
Finally, many participants cared for a patient alongside other workers who entered and left the home each day. This added to their fear of transmitting COVID-19 to their patients and to one another. (Sterling 2020) (U)
**Category 6: Unsafe storage and disposal of material and equipment in the home**
Materials, tools and equipment needed for home-based nursing care were stored in various ways, sorted in plastic or cardboard boxes with or without a closable lid or loose in plastic or paper bags. These boxes or bags were kept on tables, chairs, the floor or under a bed. In some cases, a cupboard, dresser or desk was used to store materials using different drawers for different materials. In a few cases, there was no obvious place to store materials. (Wendt 2022) (U)
In some cases, sharps containers were full or filled above the ‘do not exceed’ line or handled inappropriately. (Wendt 2022) (U)
Some used materials and waste were also found scattered across various surfaces for the client or someone else to throw away. Additionally, food waste was observed in a few instances. On occasion, contaminated waste from a nursing procedure came into contact with other persons such as toddlers and spouses. (Wendt 2022) (U)
**Category 7: Contact with animals in the home**
Environments where pets were present also put patients at higher risk of infection. Nurses viewed the mere presence of a pet as introducing an additional source of risk. However, they also highlighted how, for some patients, their general hygiene practices and how they cared for their pets increased their risk. (Dowding 2020) (U)
The presence of pets and/or pests was also discussed as adding to the variability during the home care visit. (Pogorzelska-Maziarz 2020) (U)
Besides the client and nurse,[…] animals […] could be present during the delivery of care, varying from none to many; for example, […]pets, farm animals and pests. (Wendt 2022) (U)

*C* credible, *U* unequivocal

The household context where the client finds him-/herself and where care is delivered forms another level of infection risks described in this synthesised finding. It is made up of four categories containing 15 unequivocal and 3 credible findings from 4 studies [7, 42, 43, 45].

**Category 4. Exposure to environmental risks in the home.** Home environments are perceived to present a number of infection risks such as dirt, clutter and hoarding, waste, pest infestations, inadequate ventilation, poor lightning, and inadequate access to clean water. These factors affect the clients'health state as well as the delivery of safe healthcare practices, as illustrated by the example extract below:

*“It’s hard in home health. Sometimes, I’m just at a loss. How do you make this happen when these people are living in what they’re living in? Because a person can look like they got it all together on the outside, and then you get into their home and hoarding situations, infestations of animals. […]”* [42] (Appendix 9, p.1786).

**Category 5. Exposure to frequent personal interactions and unsafe caregiving by family members.** The home environment often implies close contacts with family and visitors where family members commonly also deliver care to the client. Their knowledge, attitudes and adherence to safe care practices and infection risk prevention therefore influence risk of infection in home care.

*"During the delivery of care, more and more visitors arrive. First one adult woman. Then one adult female and adult male with toddlers (approx. 14 months). Then another adult woman with a baby. Toddlers run around the room, sneeze three times successively on chair, wheelchair and side table without covering nose or mouth. […]”* [43] (Appendix 9, Q3).

Specifically, during the Covid-19 pandemic, increased safety measures among family members and family caregivers were observed. Concern also arose regarding the infection risk posed by several different staff working for the same client.

*“There are 5 of us that work with her… What happens if we all get sick?”* [45] (Appendix 9, p.1456).

**Category 6. Unsafe storage and disposal of material and equipment in the home.** The home environment is considered to represent increased infection risk when it is impossible to safely store healthcare supplies due to lack of space or when unsafe waste disposal is carried out.

*“Sticking out of the needle container are plastic blister bags that had medication in them. Before the needles can be thrown away, they must first be removed. The client says: ‘I’ll just empty it’. The nurse asks: ‘How will you do that?’ The client responds: ‘I’ll just chuck it in the bin’.”* [46] (Appendix 9, p.5).

**Category 7. Contact with animals in the home.** Infection risk is increased by the presence of pets and other animals. They may also hinder staff in the delivery of safe healthcare practices.

*“You can be absolutely aseptic the whole time you’re in there. [But] if there’s dog poop 10 feet away from you when you’re doing wound care, that’s a problem. Some of the home environments are not appropriate for certain kinds of patients. There’s no way I can get them healed in that environment.”* [42] (Appendix 9, p.1786).

**Synthesised finding 3. Home healthcare staff form a central role in IPC during service delivery and procedures. Staff noncompliance to disinfection and safe practices pose direct infection risks for the client (**Table 6**).**

**Table 6:** Synthesised finding 3: Home health care staff form a central role in infection prevention and control during service delivery and procedures. Staff noncompliance to disinfection and safe practices pose direct infection risks for the client

**Category 8:** **Staffs'nonadherence or irregular performance of hygiene, disinfection and safe procedures (Related to the Covid-19 pandemic, staffs'increased disinfection practices were observed.)**
The workspace was cleaned irregularly before use. When the surface was cleaned with an alcohol- based disinfectant, it was regularly visibly wet when materials were placed on it. Sometimes communication devices or workwear touched the clean workspace. (Wendt 2022) (U)
Gloves used to put on compression stockings were regularly not cleaned before or after use, and when they were cleaned, in most cases hand disinfectants were inappropriately used. In some instances, these gloves stay behind in the house of the client to be used by different nurses, but on other occasions nurses take the gloves with them and use the same gloves to help multiple clients. (Wendt 2022) (U)
Two forms of hand hygiene were observed, one using an alcohol-based disinfectant and another washing at a washing stand or kitchen sink (with or without soap). Nurses, professional caregivers and clients then dried their hands in various ways, such as using paper tissues, cotton towels or the sides of their uniforms. [...] The observations revealed that hand hygiene was varying, inconsistent and irregular. (Wendt 2022) (U)
Sometimes single-use disposable materials –gloves, surgical masks, aprons and overshoes –were used incorrectly or irregularly, were re-used or were ‘cleaned’ with hand disinfectants. (Wendt 2022) (U)
Both the use of safety needles and regular needles were observed. (Wendt 2022) (U)
Participants went to great lengths to take COVID-19 precautions while in patients’ homes. They described engaging in elaborate cleaning routines whenever possible during their shifts. (Sterling 2020) (U)
Licensed staff said that communication about the spread of infection was lacking and that the assistant nurses did not always use face masks in a correct way and worked in private clothes. (Tavemark 2022) (C)
The protective equipment also affected the encounter with the older adult. All professionals emphasized that the care recipients commented that it was difficult not being able to see who was behind the mask. They could become anxious and had difficulty recognizing the staff. One participant described similar difficulties for care staff with hearing loss, as both the older adult and the employee’s colleagues had face masks during home visits. This employee therefore chose a visor instead of a face mask, so that lipreading was possible. (Tavemark 2022) (C)
Another facet of this issue was managing staff’s adherence to public health measures when they came into the family’s home. (Baumbusch 2022) (U)
Because the staff were employed by the local health authority, they were provided with personal protective equipment (PPE) that they were expected to wear while inside client’s home. Managing staff’s use of PPE is another additional role for caregivers during the pandemic. (Baumbush 2022) (U)
Aides’ infection prevention tasks also intensified as they took on more rigorous cleaning and sanitizing and maintained cleaning supplies and PPE for themselves and the veteran, as well as reporting on veterans’ and their own symptoms to the agency. (Franzosa 2022) (C)
Aides recognized patients’ anxieties over potential infection and took proactive steps on their own to make them feel safer. One aide agreed to wear extra PPE and two masks, while another described undergoing private COVID-19 testing every 2 weeks even though it was not required so she could reassure her client that she was not carrying the disease. (Franzosa 2022) (U)
**Category 9: Staffs'irregular use of workwear, electronical devices and personal equipment**
Considerable differences were found in clothing worn while providing care, from casual, day-to-day clothing to uniforms. […] Additionally, it was often up to nursing staff to clean their own workwear, but casual clothing is not cleaned at the recommended temperature, as nurses find this temperature would damage their clothing. (Wendt 2022) (U)
Nurses pay little or no attention to the bags they carry to and from different households. (Wendt 2022) (U)
A variety of electronic communication devices were used before, during and after the delivery of home-based nursing care. The most frequently used devices were (smart)phones and computer tablets, and these were often used simultaneously. Additionally, devices were constantly being carried around for consulting electronic health records or to examine nurses’ schedules. (Wendt 2022) (U) The nurses viewed their computer/tablet as a potential vector for infection, particularly as patients were meant to use their finger or a stylus provided by the nurse to sign the tablet as a record of their care provided during the home visit. (Dowding 2020) (U)
The protective equipment contributed to a feeling of security for the staff in their work at the older adults’ homes. But the care recipients’ worry increased when the local newspaper wrote that staff did not use protective equipment correctly. (Tavemark 2022) (C)
**Category 10: Staff experiences of stress and distractions during care **
Participants described staffing challenges faced by home health care agencies because of the aging workforce and poor retention and recruitment. Participants explained how this impacted patient care including adherence to IPC and how it affected current staff who had to take on additional responsibilities. (Pogorzelska-Maziarz 2020) (U)
In addition to being physically present during the delivery of care, communication devices also tend to be distracting because they can interrupt nursing procedures. The latter is especially true because nurses feel pressure to answer the phone in case the call involves peer consultations or possible difficulties. (Wendt 2022) (U)
The constant teaching and reminders regarding self-protection protocols further complicated daily routines and exacerbated caregiver stress. (Baumbusch 2022) (U)

*C* credible, *U* unequivocal

The influence of home healthcare staff care delivery practices on infection risk is described in this finding. It describes staff attitudes and behaviours as well as the challenges involved in safe care delivery in the home context. The synthesised finding is made up of three categories containing 16 unequivocal findings and four credible findings from six studies.

**Category 8. Staffs'nonadherence or irregular performance of hygiene, disinfection and safe procedures.** Infection risks occur when staff do not perform adequate disinfection procedures, when hand hygiene and use of safety equipment is poor or inexistent. Illustrations included the following:

*"Initially, the nurse does not wear an apron while providing care. The client herself points out to the nurse that when the research is about the prevention of infections, the nurse should be wearing an apron.[…]”* [43] (Appendix 9, Q6).

Related to the Covid-19 pandemic, staffs'increased disinfection and infection prevention practices were observed.

*"I take precautions. I wear my mask. I keep sanitizer on me. I wash my hands constantly but I still give them the attention they need, you know, it’s very hands-on."* [50] (Appendix 9, p. 1834).

**Category 9. Staffs'irregular use of workwear, electronical devices and personal equipment.** Contrary to a hospital environment, home healthcare staff are observed working in personal clothing which may be inadequately sterilised and as such represent infection risks. The frequent irregular use of bags and electronic equipment during the workday of home visits to several clients is further observed as a risk factor.

*"That computer, I take it out of that bag, it sits on the table at the client, and it goes back in my bag. And it ends up on the next client's table."* [43] (Appendix 9, Q1).

**Category 10. Staff experiences of stress and distractions during care.** Stress caused by high workload and time pressure, as well as distractions in the form of frequent telephone calls during care delivery were perceived to increase the risk of performing unsafe procedures.

*“The nurses I can say, they are just so stressed up. They have too many patients to see, and they want to make sure that they covered everything. Now they are shortcutting, the tendency of shortcutting is you are giving a high percentage of committing mistakes.”* [42] (Appendix 9, p.1784).

**Synthesised finding 4. Home care agencies hold key responsibilities in providing staff with sufficient and relevant equipment, information, guidelines, training and support. Low agency priority of these aspects including lack of organised provision of necessary equipment to family care givers represents considerable barriers to safe delivery of home care, while agencies’ investments into coordinated IPC efforts are perceived as important positive protective measures (**Table 7**).**

**Table 7:** Synthesised finding 4: Home health care agencies hold key responsibilities in providing staff with sufficient and relevant equipment, information, guidelines, training, and support. Low agency priority of these aspects represents considerable barriers to safe delivery of home health care, while agencies investments into coordinated infection prevention and control efforts are perceived as important positive measures

**Category 11: Lack of access to equipment**
On occasion, nurses lacked the right materials for in-home care, such as disinfectants, gloves and aprons. (Wendt 2022) (U)
Further exacerbating this challenge was the limited availability of adequate hand hygiene products in patients’ homes. (Osakwe 2021) (C)
Many home health aides experienced challenges with having an adequate supply of personal protective equipment (PPE) during the pandemic. Some noted structural barriers related to the home health care office being closed when their shifts end, making it hard to get access to supplies. (Osakwe 2021) (U)
Many home health care workers also reported that they lacked adequate PPE from their agencies, including masks and gloves, which they felt was essential for care. (Sterling 2020) (U)
It was also noted that accessing pandemic resources, such as PPE, was a challenge. Home-based care providers described not being well-connected or prioritized in terms of public health resources. Community-based organisations were found to be more available as a support to acquire PPE than government resources. (Bell 2022) (U)
Some agencies even reported having a stockpile in place due to previous experience with communicable disease outbreaks. (Bell 2022) (U)
The participants explained that at the beginning of the pandemic they were not allowed to wear protective gear in the form of masks or face shields when in close contact with patients because of the scarcity of protective gear, which worried them. (Emmesjö 2022) (C)
The authorities had stated that this protective gear was unnecessary when working with older patients, and there was a nationwide lack of protective gear. The participants explained how they had been told that protective gear needed to go to hospitals instead, because of the scarcity of it. (Emmesjö 2022) (C)
Some participants bought their own protective gear when it was not supplied by the municipality or primary health care center. When the protective gear was allowed to be worn, the participants were relieved since it was a relief easing the worry of infection. (Emmesjö 2022) (C)
After protective gear was permitted, the way RNs and MICM-physicians worked changed. Protective gear was used constantly when the RNs and MICM-physicians were within two meters of a person during work, and the participants became less worried about infecting someone or being infected themselves. (Emmesjö 2022) (C)
One challenge that was particularly prevalent at the start of the pandemic was the lack of equipment. One nurse told how they ordered huge amounts of infection control equipment and yet received only small numbers. (Moi 2022) (U)
Caregivers described the additional economic burden of the pandemic measures. This situation highlights one way in which pandemic response measures disproportionately impacted people with low incomes, who are often older people with fixed incomes. (Baumbusch 2022) (C)
There was also scarcity of supplies that they were already using, and now stores were limiting purchases or sold out entirely. She also shared how stores increased prices in response to pandemic demand. (Baumbusch 2022) (C)
F11.3: Although most administrators reported having sufficient supplies of PPE, some aides described shortages or difficulties obtaining it. (Franzosa 2022) (U)
**Category 12: Lack of access to clear information, guidelines and communication**
Nurses, professional caregivers and clients found that different protocols were given by different institutions, hospitals and colleagues, resulting in fragmentation, variation, discrepancies or conflicting information. […] Furthermore, these nurses, caregivers and clients recognized differences between home-based nursing care teams in dealing with certain situations, guidelines and protocols,[...] At times, these nurses and professional caregivers doubted the accuracy of the information they received. (Wendt 2022) (U)
Nurses indicated that they had reservations regarding whether the World Health Organisation’s recommended ‘Five Moments for Hand Hygiene’ ‘fit’ the home-based nursing care environment (Wendt 2022) (U)
Generally, in offices or in the nurses’ cars, cleaning or disinfecting wipes were available, but the nurses doubted the right ways (method and materials) and times to clean [electronic devices]. Thus, these wipes were used in only a few instances. (Wendt 2022) (C)
Nurses sometimes experienced untimely or incomplete transfers of clients’ health records when clients were transferred from other care environments to their homes. For instance, this can occur when a client carries a multidrug-resistant organism. (Wendt 2022) (C)
Although some agencies adapted quickly to the pandemic by providing workers with COVID-19–related information on a weekly or daily basis, others reportedly barely communicated about the pandemic. (Sterling 2020) (U)
Home-based care providers and their patients’ experienced frustration around the lack of clear and consistent information from public health officials on health, safety, and wellbeing during the novel pandemic. The delayed timing and execution of pandemic messaging hindered both providers’ and patients’ preparedness. [...] Providers felt they were not able to pass along adequate information to patients, as the information was uneven and, in some cases, conflicting. Likewise, the lack of response from public health authorities left home-based care provider agencies confused on what care measures were appropriate given the uncertainty around the virus. (Bell 2022) (U)
To cope with this lack of information, home-based care provider agencies turned to the Centers for Disease Control and Prevention (CDC) or their own infection control plans; however, the novelty of the virus left many to question what to do to protect themselves and their patients in the onset of the pandemic. (Bell 2022) (U)
Among Spanish-speaking participants, gaining access to COVID-19 related information necessary to facilitate patient care was a challenge. Spanish-speaking home health aides preferred working with Spanish-speaking patients. Another home health aide expressed how language posed a barrier to effective communication with non-Spanish-speaking home health care nurses. [...] Because of limited English proficiency, Spanish-speaking home health aides had to locate resources to facilitate communication with patients, families and the health care team, and to understand COVID- 19 related information. Many Spanish-speaking home health aides thus relied on their family members to translate care plans or clinical information. (Osakwe 2021) (U)
F7.6 To prevent transmission, the participants explained that they followed the authorities’ restrictions, even if the recommendations changed often. The changing directives were seen as challenging, but the RNs and MICM-physicians worried less about transmission if they knew that they were following up-to-date recommendations. The MICM-physicians or RNs held information or visited personnel meetings to answer questions from the ANs [assistant nurses] about COVID-19, as the participants noticed that the ANs needed additional l knowledge to ease their worries about infecting others. (Emmesjö 2022) (C)
The routines for the protective equipment were interpreted differently and changed rapidly. There were differences in which protective equipment was used and how it was handled, whether it was put on in or outside the home, and where the material was then disposed of or cleaned. Using the protective equipment caused physical problems in the work environment. Participants said that they needed to manage it outside the home and it could be cold to clean the equipment in the cars in winter. (Tavemark 2022) (C)
Nurses experienced a significant lack of information and explicit guidelines at the start of the pandemic. When the public guidelines emphasized the extensive benefits of using a face mask, the nurses were instructed not to use one. (Moi 2022) (U)
Significant uncertainty was prevalent both nationally and locally and the uncertainty was reinforced in their specific work situation. (Moi 2022) (U)
**Category 13: Restricted access to Covid-19 testing **
Home health aides indicated that they wanted to know if their patient or patient’s family had tested positive for COVID-19 but emphasized that this information was not available. They also expressed concern regarding the lack of information about the COVID-19 status of other home health aide colleagues who also care for their patient and how this might put them at risk for exposure to COVID-19. (Osakwe 2021) (U)
In addition to uncertainty regarding the COVID-19 status of others, HHAs worked with a fear of not knowing their own COVID-19 status because they had not been tested. […] Home health aides expressed frustration with the limited information they received from home health care nurses or their agency about getting tested. (Osakwe 2021) (U)
While home health aides expressed concerns about lack of access to testing, many HHAs appreciated the daily screening COVID-19 which their agencies conducted via telephone. (Osakwe 2021) (C)
Some agencies asked participants to perform daily “self-assessments.” Self-assessments, which were usually automated by phone, were intended to screen home health care workers for COVID-19 symptoms. Depending on how they answered, workers would be encouraged to go to work or to call their doctor. (Sterling 2020) (U)
**Category 14: Limited opportunities provided for staff training and knowledge transfer**
Several participants with a role in infection prevention and control (IPC) talked about their lack of formal training, as well as the limited number of resources focused on IPC specifically in home health care. (Pogorzelska-Maziarz 2020) (U)
Furthermore, a high workload played a negative role in knowledge transfer. Beyond this, the fact that employees are not paid for time spent on knowledge transfer was seen as an impediment. (Wendt 2022) (U)
Working alone made it very difficult for nurses to observe their colleagues or to discuss infection prevention practices. In such cases, the implementation and evaluation of new information or policy changes were problematic. (Wendt 2022) (U)
Having an agency dedicate time and money toward staff education (whether preemptive or reactive) was viewed as critical for clinicians to be able to provide quality care to their patients. (Pogorzelska-Maziarz 2020) (U)
A number of participants viewed their prior training as a contributing factor to their preparedness during the pandemic. They described how frequent contact with patients with infectious conditions prior to the COVID-19 pandemic contributed to a sense of mastery in infection control practices. The education they received prior to the onset of the pandemic provided them with foundational knowledge in infection control practices, particularly around use of personal protective equipment (PPE) and protocols intended to minimize the spread of infection such as hand hygiene. (Bell 2022) (U)
**Category 15: Importance of home health care agency infection prevention and control preparedness and coordination**
Yet, not all agencies, or providers felt as prepared. While some agencies did have a pandemic plan, providers were either not familiar with its details or felt their infection control plan fell short given the unknown needs of this novel pandemic. (Bell 2022) (C)
Several participants described specific ways in which agencies have improved teamwork and care coordination. (Pogorzelska-Maziarz 2020) (U)
Furthermore, having leadership support and encouragement allowed staff members to take on IPC as a key initiative. (Pogorzelska-Maziarz 2020) (U)
Finally, some agencies described utilizing real-time data, such as data from their electronic medical records, as a key to success because they were able to direct their often-limited resources to target specific areas for improvement, often related to IPC. (Pogorzelska-Maziarz 2020) (U)
To get information about the spread of infection in the municipality, the staff had a review of the infection situation at the unit every morning and afternoon. At the daily meetings, the content of the day’s home visits was prioritized with the support of the manager, something that was perceived as valuable, especially at the beginning of the pandemic. At these meetings, it was also verified that everyone had been informed in the event of an infection. Nurses and unit heads had a great responsibility, but it was not clear who they should inform. (Tavemark 2022) (U)
As well as challenges associated with obtaining enough equipment, participants experienced the importance of how their immediate superior and their district manager acted after the outbreak of the pandemic. Many had positive experiences with their manager regarding how they dealt with the challenges. (Moi 2022) (U)
The participants had gained a much better understanding of infection control than previously, in addition to the increased attention from the wider society. Infection control routines were drawn up in all the districts and participants felt the routines to be clear. They had gained knowledge and experience that gave them increased confidence to confront new pandemics. (Moi 2022) (U)

*C* credible, *U* unequivocal

This synthesised finding is focused on the influence on IPC from the agency level. The findings describe barriers as well as facilitators related to home care agencies policies, support procedures and investments in infection prevention and control. This finding also included the lack of organised measures to provide family caregivers with necessary equipment for infection prevention. It is made up of five categories containing 30 unequivocal findings and 12 credible findings from eight studies.

**Category 11. Lack of access to equipment.** Agencies’ failure to ensure adequate hand hygiene products and personal protective equipment (PPE) in clients' homes impede IPC practices. Lack of protective equipment was especially highlighted in relation to the Covid-19 pandemic, however, not in all home care agencies. Family caregivers also struggled to get PPE during the pandemic.

*“I’m worried about getting infected because I don’t have the right equipment. The agency has not really been providing for their workers, at* all.” [45] (Appendix 9, p.1457).

*[The client’s agency]"is giving me like, five masks, and that’s it. So, I don’t even think about it, just bring my own bag, I bring my own gloves, my mask, everything.”* [50] (Appendix 9, p. 1834).

**Category 12. Lack of access to clear information, guidelines and communication.** Staff receiving incomplete information on clients' health state, protocols or guidelines is perceived to be an impediment for safe care delivery in the home care environment. Conflicting messages further contribute to staff confusion and doubt around IPC. Lack of agency support to non-English speaking staff is another barrier to communication and information access.

*“Is it really necessary for us to do this hand hygiene –even within the client’s house –or is it unnecessary in the home environment? Because that is all based on research done in the hospital.”* [7] (Appendix 9, p.5).

**Category 13. Restricted access to Covid-19 testing.** During the Covid-19 pandemic, home health care agencies' incapacity to provide testing and access to information on Covid status of clients and staff hindered effective risk control. However, daily risk screening messages from agencies were recognized by staff.

*“I am with a patient, and she has all this different aides coming in and out. No one tells you if a home health aide had COVID, I do not know if my patient had it…”* [44] (Appendix 9, p.1365).

**Category 14. Limited opportunities provided for staff training and knowledge transfer.** The nature of home care where staff often work on their own in clients' dwellings, represents a challenge to knowledge transfers. Lack of agency investment in training is perceived to hinder the implementation of infection prevention.

*“I actually had no previous experience in home care or formally in infection prevention when I came to this role, which is a challenge. When I look at what resources are out there. [it is] very much geared toward the inpatient world.”* [42] (Appendix 9, p.1786).

On the contrary, when agencies prioritise education, it is recognised as an important driver of IPC.

*“In home health, [we have] always used infection control. We were all prepared, and we were always prepared in teaching patients about infection control in their own homes […]. In the community and in the patients’ home we were good at that, and we were prepared for a pandemic.”* [41] (Appendix 9, p.4).

**Category 15. Importance of home care agency IPC preparedness and coordination.** Agencies' lack of IPC plans and guidelines represent barriers to safe home health care, while cases where agencies have prioritised coordination, teamwork and evaluation are recognized as positive efforts towards IPC.

*"We actually are really working just individually right with that team and the team manager to really look at their own outcomes and trying to help them to improve.”* [42] (Appendix 9, p.1788).

**Synthesised finding 5. Staffs' beliefs that risk of infection to clients and themselves is inevitable due to the nature and structure of home care services (**Table 8**).**

**Table 8:** Synthesised finding 5: Staffs' beliefs that risk of infection to clients and themselves is inevitable due to the nature and structure of home health care services

**Category 16: The structure and nature of home health care, in itself, imply infection risk and challenges to infection prevention and control -**
Specifically, participants reported a certain level of unpredictability during home visits. Staff described attempting to establish clean fields and implement universal precautions in a home, which may lack what they perceived as adequate cleanliness and sanitization. (Pogorzelska-Maziarz 2020) (U)
Cluttered and contaminated households presented nurses with a dilemma. Nurses indicated that they could not refuse to provide care because clients have a ‘right to receive healthcare’, but at the same time there is a need for guidance on how to address contaminated households to minimize the spread of infectious microorganisms. (Wendt 2022) (U)
In the context of homecare practice in the USA, nurses must adjust their level of care to what is covered by the patient’s insurance, which may require some patients to monitor their own clinical status more than they would in other clinical settings. (Dowding 2020) (C)
Home health aides provided personal care to patients who had functional limitations. Home health aides consistently stated that they had no other health care professional to rely on to help with difficult tasks in patients’ homes, which may require assistance in other settings, such as turning or positioning physically dependent patients. While providing such care, home health aides had fears related to COVID-19 exposure risk. (Osakwe 2021) (U)
Home health aides placed high priority on keeping patients safe at home and free from falls. This effort frequently compromised their ability to maintain hand hygiene practices, thus increasing potential COVID-19 risk. (Osakwe 2021) (U)
Aides noted that the intimate nature of their work made it impossible to maintain physical distance, and this was a source of constant “worrying”. (Franzosa 2022) (C)
**Category 17: Lack of access to clear information, guidelines and communication**
Many home health aides described the unique challenge to keep their masks on in patients’ homes and the conflict with patient satisfaction. Mask wearing made it difficult for patients with hearing impairments to fully understand home health aides when speaking. As a result, HHAs reported being caught between maintaining infection prevention practices at the risk of achieving high patient satisfaction—a factor critical to being retained as a home health aide with patients. (Osakwe 2021) (C)
To protect patients, participants went to the grocery store and pharmacy on their behalf, which increased their own risk for contracting COVID-19. Although sometimes they volunteered, other times they were asked. F6.3. Participants also worried about their own risk of contracting COVID-19, and nearly all felt that their dependence on public transportation increased this risk. (Sterling 2020) (U)
Participants also worried about their own risk of contracting COVID-19, and nearly all felt that their dependence on public transportation increased this risk. Many participants reported using public transportation to get to their patients’ homes, to run errands for them, and to travel to their agency for supplies. (Sterling 2020) (U)
Taken together, they tried balancing the risks of work with their own health and financial well-being. (Sterling 2020) (U)
Many spoke about balancing the risks of caring for patients during the COVID-19 pandemic with the duty or “calling” they felt to help patients. (Sterling 2020) (U)
It was clear that the participants saw the risk of becoming infected as part of their work. (Tavemark 2022) (U)

C, credible; U, unequivocal

This synthesised finding presents staffs’ reflections on their limited capacity to ensure IPC as a result of the structure of home care services, and the restricted control they exercise over the work conditions. It is made up of two categories containing nine unequivocal findings and three credible findings from seven studies.

**Category 16. The structure and nature of home healthcare, in itself, imply infection risk and challenges to IPC.** Performing healthcare services in the home environment is perceived to involve lower levels of control over the patient, procedures and environment, than in a hospital setting. Staff working alone or with limited supplies reduces the capacity to avoid situations of risk exposure.

*“Well, it’s not the hospital where it’s a controlled environment. You’re going into patient’s homes that sometimes aren’t the cleanest. You just gotta do the best you can and try to be as clean and prevent infections as you can in the home. You’re working with what you have.”* [42] (Appendix 9, p.1784).

**Category 17. Staffs' own exposure to risk is believed to be part of the job.** Delivering home care is perceived to involve unavoidable staff exposure to infection risk, especially during the COVID-19 pandemic. This risk is weighed against a call of duty or a need for employment.

*“He needs to stay inside the house, so he tells me, ‘I need you to go there, go here.’ I really don’t want to, but I can’t say no. I’m the aide; I’m supposed to do this.”* [45] (Appendix 9, p.1456).

*“You have to contribute certain hours to get benefits... I have to go out there because I have bills to pay.”* [45] (Appendix 9, p.1457).

*"In November I got infected, but I’m not really worried, it’s more natural because of my work, I can’t do much more.”* [46] (Appendix 9, p.9).

### Integration of quantitative and qualitative evidence

#### Congruence between synthesised qualitative findings and quantitative findings

Numerous synthesised qualitative findings were in congruence with several of the quantitative findings, allowing for a configuration of evidence as outlined below.

The first synthesised qualitative finding, known as category 1, and several findings from the quantitative studies showed that patients’ individual characteristics, behaviours and health status determine their infection risk. More specifically, category 1 referred to individuals’ history of infections, specific medical conditions and the need of medical devices increased their risk of infections. Similarly, findings from the cross-sectional and cohort studies showed increased infection risks for patients with nasogastric tubes, history of hospitalizations and/or previous infections, along with a number of specific medical conditions. The meta-analysis further emphasized this congruity by showing increased risk of infection among patients with urinary catheters, a risk factor also identified in the qualitative studies.

Category 2 showed that the presence of co-morbidities increased infection risks. This provides congruent evidence that multiple medical conditions constitute an increased risk for infections among older adults receiving home care. Moreover, category 2 revealed that interaction between patients’ multiple health conditions, such as decreased cognitive capacity and urinary incontinence, and availability of help from caregivers, increased their infection risk. This is in line with the quantitative findings showing that environmental and social risk factors, such as the presence and availability of caregivers, impact infection risks. Finally, category 3 showed that patients’ understanding and ability to adhere to infection control practices were key to infection control. This was supported in the meta-analyses indicating that cognitive impairment and restricted mobility increased the risk of infectious disease.

The second synthesised finding, along with quantitative evidence from cohort and cross-sectional studies, showed that interactions between patients and other members of the household posed infection risks. Particularly category 5 revealed that the practices of family members, who often also served as caregivers, were crucial for infection control, in congruence with quantitative evidence that organisational factors such as caregivers’ need for training constituted increased risk of infection for home care patients. Category 6 brought forth the risks of infection associated with unsafe disposal of materials in the home, a finding in line with a cross-sectional study that showed increased respiratory infection risks in housing settings where the waste disposal system was not connected to the public network.

The third synthesised finding identified that home care staff’s behaviours affect infections risks. Category 8 and 9 showed that staff compliance with infection prevention practices, such as disinfection routines and safe workwear usage were linked to infection risks. This is in accordance with findings from the quantitative studies showing that staff training on infection prevention practices, and staff’s need of training, were associated with increased infection risks.

Synthesised finding 4 showed that agencies’ priorities to provide home care staff with necessary guidelines and support were essential for infection control. For instance, category 12 showed that lack of access to clear information and guidelines impeded infection prevention, which is in congruence with quantitative findings showing that the presence and implementation of agency policies that promote best practices for care of patients with urinary catheters decreased hospitalizations for UTI. Moreover, category 14 identified the lack of opportunities for staff training as an infection risk, supported by quantitative findings suggesting that organisational factors such as staff in need of further training increased the infection risks among home care patients.

In accordance with the JBI Manual, we have summarised aspects of the qualitative findings that were not examined in the quantitative studies, and vice versa. These are available in Appendix 10.

## Discussion

Using a convergent, segregated approach, this mixed methods systematic review aimed to identify risk factors for infection in older adults who receive home care. Main results show that older adults in home care are at risk for infection due to a range of individual, medical, environmental, and organisational factors. Some of these factors include being in need of medical devices, having limited mobility, and having a history of previous infections and comorbidities. Several infection risk factors are of a structural and organisational nature, such as staff in need of training, lack of access to equipment, working under unclear guidelines or high stress and heavy workload. Moreover, the home environment itself carries risks, for example by being cluttered or by posing difficulties in terms of waste management and lack of sterile surfaces. Finally, the review showed that personal behaviours and attitudes towards infection prevention practices are crucial for infection control, and that these factors interplay with organisational level obstacles to optimal infection prevention practices.

The results of the meta-analysis, which showed a fourfold greater risk of infection due to urinary catheter use (OR 3.97, 95%CI 2.56–6.15) in older adults with home care, adds to previous research on infection risk of urinary catheter use in patients in general. Whilst this finding refers to studies on hospital-acquired infections among older adults with home care, it stresses the importance of infection prevention in their homes to avoid additional co-occurring infections that may cause a rapid decline in the health of this already vulnerable group of older adults [54]. The review further adds to existing research on limited mobility as a potential predictor for risk infection [55] by demonstrating a 1.5 time (OR 1.49, 95%CI 1.31–1.68) greater risk of infection among older adults with limited mobility. Body movement reduces the risk of long periods of urinary stasis, known for increasing the risk of UTI [56]. Urinary flow also reduces bacteria in the bladder [57], highlighting the vulnerability of inactive bedridden or chair-fast home health care patients. Additionally, mobility training has also been shown to reduce incontinence in older women [58]. In the meta-analysis, urinary incontinence was not statistically significantly associated with UTI. The lack of association remained also when adding a study not restricted to UTI but also including respiratory, wound, and intravenous catheter-related infections. The finding is inconsistent with previous research. For instance, a study of healthy postmenopausal women reported that urinary incontinence was more common in women who experience UTIs than those who do not [59]. It is possible that the lack of association in the meta-analysis was driven by a single study, in which the study population sample consisted of groups more likely to be hospitalised for reasons other than UTI [27].

Whilst informal caregivers play an important role in the care of patients who live in their own homes, the findings of this review indicate that inadequately trained caregivers may put the patients at risk of infection. In several qualitative studies [7, 42, 43, 45], unsafe caregiving by family members posed infection risks to home care patients, and similarly, the quantitative findings showed that caregivers in need of training increased infection risks [3, 5]. However, little detail about the type of training required was provided in the included studies. Training on use of devices, administration of medications, recognizing symptoms, or general infection prevention measures were examples suggested. Future well-planned and evaluated interventions that draw upon evidence from both quantitative and qualitative research are needed as evidence of effective interventions is lacking. A systematic review of interventions to improve hand hygiene demonstrated few improvements and concluded that performance feedback, education, and informative signs encouraging hand washing may work, but reported certainty of evidence to be low [60].

Employing an IPC nurse trained in all infection prevention activities and thus able to ensure supervision, support and implementation of routines has been successful [43] but may not be feasible at larger scale. Furthermore, delivery of certain kinds of care should be restricted to registered medical professionals while training of informal caregivers ought to focus on aspects of caregiving that medical and other professionals do not usually provide. Though collaboration between health and social care is essential, having professionals focusing on their area of expertise benefits both the older person who safely receives the best care, as well as the professional who then carries out work that they are confident in undertaking. This should not be confused with some professionals’ desire to uphold their role and status to ensure that their professional perspective has a dominant position [61] but allow for different professions to focus on what they are specialised in and to build collaborations based on differences in expertise.

The findings further indicated that structural level factors, such as agency policies and guidelines, play an important role for infection risk and control [34, 36, 39, 41, 43–48]. For example, the existence of policies on catheter management was shown to reduce infection risks [34], whereas lack of clear guidelines was considered an obstacle to safe care delivery by home care staff [43, 45, 46, 48]. Agencies’ reporting on infections should however be carefully interpreted as the presence of an IPC committee in agencies may increase the number of infections reported [36]. This might be due to agencies with IPC committees having more comprehensive structures for reporting infections, and consequently, better at infection surveillance, compared to agencies without such committee.

Findings of the current review furthermore demonstrate that behavioural and environmental factors are important for infection control in the home care setting on their own yet intertwined with organisational features. Thus, care providers’ practices, such as adherence to hygiene routines, are crucial, but so are the structures that inform, impede, or facilitate such practices. Similarly, the broad approach of the current systematic review allow for reporting on both knowledge and attitudes [6] as well as structural features, which may be equally important for infection control in the home setting [41, 42, 46]. Indeed, knowledge on how to perform infection control procedures correctly is not enough if there is a lack of access to necessary protective equipment and testing, or if working conditions are marked by high stress, heavy workload, and lack of materials. Besides, there are national cultural dimensions influencing infection control behaviour shaped by, for example, local outbreaks and vaccination rates, that interplay with governmental initiatives and risk management strategies [62]. Consequently, the interaction of different level risk factors must be considered for a rigorous understanding of IPC in the home environment.

### Study limitations

Study strengths include the broad range of risk factors for infection explored and the inclusion of both qualitative and quantitative studies. The search strategy was not restricted to a certain language, geographical area, or time period. Search terms were further developed together with two well-experienced librarians and applied to five different databases. PRISMA and JBI guidelines were used to ensure a systematic and transparent approach, and two reviewers independently assessed the studies.

Study limitations include the lack of inclusion of grey literature, possibly resulting in relevant studies not being considered. Most of the included studies were from high income countries and only one study from the global south (Brazil), limiting the generalizability of the findings. The inclusion of cross-sectional studies restricted the possibility to determine the direction of causality of the observed relationships in the meta-analyses. Though the prospective studies shed light on the direction of causality, combining results from cross-sectional and prospective study designs restricts conclusions on causality and is associated with risks of bias. Additionally, exclusion of studies following quality assessment was not determined a priori but following discussion. Further, the review process took longer time than expected and an updated search was undertaken. Furthermore, many of the included quantitative studies used self-reported rather than objectively assessed data for exposure and outcome measures. Lastly, we initially intended to apply an age limit of 65 years, however, several studies did not report on their participants’ exact age. Therefore, it was decided to include studies of which the population receiving home care was reported to be of older age.

### Policy and research implications

#### Recommendations for practice

The integration of qualitative and quantitative findings has several implications for practice and policy. The present review shows that, while staff behaviour is crucial for infection control, agencies must prioritize infection control co-ordination and preparedness, including supply of materials and staff training opportunities to enable optimal IPC practices among care-providers [3, 5, 41, 42, 46, 48]. As reported in the included qualitative studies conducted during or post the Covid-19 pandemic, organisational aspects of infection control are key for home care staff to be able to adhere to safe infection prevention practices and need to be addressed in future policies.

Regarding medical and individual risk factors, urinary catheter use emerged as a common risk factor for UTI and other infections. Given the infection risks associated with catheter use in combination with high demand in the older age home care population, best practice for catheter use is crucial for optimal infection control and prevention. Guidelines for catheter use in surgery recommend using urinary catheters only when needed and removing them as soon as possible after the operation [63]. Similarly, The Royal College of Nurses recommends early removal of catheter where possible [64]. Educating healthcare staff on prevention of urinary tract infection has been shown to reduce catheterization time. Educating healthcare staff about the latest guidelines on how to prevent catheter associated UTIs was further deemed vital for infection control in a recent systematic review [65]. The present systematic review also identified a need for and benefits of educating staff on prevention control. These findings support existing research and guidelines and adds to policy recommendations by showing that education and training on prevention control need to also include informal caregivers. Further, findings also indicate that the home environment itself poses several risk factors for infections, for instance in terms of clutter and the presence of pets. Consequently, home healthcare and home help agencies need to take these barriers to infection control into consideration when training and educating staff and informal caregivers. Cost-effectiveness evaluations may further facilitate resource allocation and healthcare planning. While educating and training staff often are associated with costs, under-provision of training, equipment, and environmental modifications might come at a cost, too. Health economic analyses could provide important input on the optimization of home care delivery. Moreover, to improve detection of UTIs, diagnostic criteria may need to be modified, since UTIs in later life are often asymptomatic and unspecific, and thus difficult to discover [28, 66, 67]. Detection and infection surveillance methods that are adapted and adjusted to the often asymptomatic infections that occur in older adults have the potential to reduce the risk of delayed diagnosis and suboptimal treatment.

#### Recommendations for further research

Further research on this subject area, applying both specific and broader perspectives, is needed. Specific perspectives to be studied include individual aspects that influence risk of infection such as the role of socio-economic background, ethnicity and geographical area, and the influence of medications on infection risk. This should furthermore be investigated using objective and longitudinal data to better understand the causal relationships between various risk factors and infections among older adults receiving home care. It also has the potential to shed light on possible health disparities within this population. Qualitative studies of older adults’ perceptions and experiences with infection risks and control in the home care setting would further enrich the understanding of these individual aspects.

A broader perspective is needed to explore potential interactions between the identified structural and organisational factors with medical and individual risk factors in relation to the risks of working in the home environment. This may include innovative methodologies, interdisciplinary collaborations, and novel interventions. Studying how these separate yet interconnected dimensions of risks may reinforce each other, exposing vulnerable older adults to amplified risks, could inform policies and practices aimed at mitigating such effects.

## Supplementary Information

Supplementary Material 1

Supplementary Material 2

Supplementary Material 3

Supplementary Material 4

Supplementary Material 5

Supplementary Material 6

Supplementary Material 7

Supplementary Material 8

Supplementary Material 9

Supplementary Material 10

## Data Availability

All data generated or analysed during this study are included in this published article and its supplementary information files.
