# Supplementary material for: Risk factors for infection in older adults with home care: a mixed methods systematic review with meta-analysis
Source: BMC Public Health. 2025 May 3;25:1643. doi: 10.1186/s12889-025-22538-1 (PMC12048934; doi:10.1186/s12889-025-22538-1)
Supplement: Supplementary file 2 — Supplementary Material 2 [file 12889_2025_22538_MOESM2_ESM.pdf]

Documentation of search strategies
University Library search consultation group

Date: 20 May 2022

Topic/research question: A systematic review of risk factors for infection in older adults who receive home healthcare and/or home help

Name of researcher(s): Ann Liljas, Department of Global Public Health

Librarian(s): Anja Vikingson & Sabina Gillsund

Databases:

1. Medline (Ovid)
2. Embase (embase.com)
3. Web of Science (Clarivate)
4. Sociological Abstracts (ProQuest)
5. Cinahl (EBSCO)

Total number of hits:

- Before deduplication: 28083
- After deduplication: 17984

Comments:

Deduplication based on the method described in:
Bramer, W. M., Giustini, D., de Jonge, G. B., Holland, L., & Bekhuis, T. (2016). De-duplication of database search results for systematic reviews in EndNote. *Journal of the Medical Library Association: JMLA*, 104(3), 240–243. doi:10.3163/1536-5050.104.3.014

One final, extra step was added to compare DOIs.

1. Medline

Interface: Ovid MEDLINE(R) and Epub Ahead of Print, In-Process & Other Non-Indexed Citations and Daily<br><br>Date of Search: 2022-05-20<br><br>Number of hits: 8030<br><br>Comment: In Ovid, two or more words are automatically searched as phrases; i.e. no quotation marks are needed	Field labels<br><br>• exp/ = exploded MeSH term<br>• / = non exploded MeSH term<br>• .ti,ab,kf. = title, abstract and author keywords<br>• adjx = within x words, regardless of order<br>• * = truncation of word for alternate endings

#	Searches	Results
1	exp Home Care Services/	49975
2	exp Community Health Nursing/	20259
3	((domiciliary or home or homemaker or parish or respite) adj3 (care or caregiver or health care or healthcare or hhc or nurse* or nursing or therap* or service* or hemodialys* or nutrition)).ti,ab,kf.	71166
4	(community care or community health care* or community healthcare* or community health service* or community nurs* or community setting*).ti,ab,kf.	23889
5	("family carer?" or "family caregiver*" or "family care giver*" or "spouse care*" or "informal care*").ti,ab,kf.	17448
6	exp Home Nursing/	9515
7	district nurse patient*.ti,ab,kf.	6
8	or/1-7	144798
9	Infections/	40552
10	Asymptomatic Infections/	2563
11	"Bacterial Infections and Mycoses"/	0
12	Bacterial Infections/	73785
13	Coinfection/	13926
14	Communicable Diseases/	31925
15	Communicable Diseases, Emerging/	6452
16	Communicable Diseases, Imported/	530
17	Blood-Borne Infections/	53
18	Community-Acquired Infections/	15481

19	Cross Infection/	60159
20	Healthcare-Associated Pneumonia/	290
21	Latent Infection/	132
22	Latent Tuberculosis/	3559
23	Syphilis, Latent/	640
24	Opportunistic Infections/	12438
25	AIDS-Related Opportunistic Infections/	21336
26	Superinfection/	1961
27	Persistent Infection/	202
28	exp Respiratory Tract Infections/	536072
29	Soft Tissue Infections/	4048
30	Suppuration/	8061
31	Abscess/	28696
32	Empyema/	4330
33	Pyomyositis/	437
34	Vaccine-Preventable Diseases/	174
35	Virus Diseases/	40855
36	Bronchiolitis, Viral/	1599
37	DNA Virus Infections/	2066
38	Hepatitis, Viral, Animal/	1641
39	Hepatitis, Viral, Human/	11481
40	Pneumonia, Viral/	45633
41	Waterborne Diseases/	226
42	Wound Infection/	11731
43	((air-borne or airborne or asymptomatic or bacterial or blood-borne or bloodborne or coinfection or co-infection or communicable or community-acquired or chronic or cross or "HIV" or infectious or latent or long term or mixed or opportunistic or persistent or polymicrobial or reactivated or respiratory tract or soft tissue or superinvasion or transmission* or transmitted or secondary or vaccine-preventable or vaccine preventable or viral or virus or waterborne or water related or water-related or wound) adj2 (diseas* or infect*)).ti,ab,kf.	1111974
44	(abscess* or bovine respiratory disease complex or bronchitis or coinfection or common cold or empyema or fungal lung disease* or healthcare-associated pneumonia or human influenza or laryngeal tuberculosis or laryngitis or latent syphilis or latent tubercolosis or legionellosis or mycoses	315335

	or parasitic lung disease* or pharyngitis or pleural tuberculosis or pleurisy or pulmonary tuberculosis or pyomyositis or rhinitis or rhinoscleroma or severe acute respiratory syndrome or sinusitis or superinfection* or suppurat* or supraglottitis or viral bronchiolitis or viral hepatitis or viral pneumonia or whooping cough).ti,ab,kf.	
45	((control or prevent* or risk) adj2 (infection* or infectious)).ti,ab,kf.	100216
46	or/9-45	1944727
47	(congress or clinical conference or comment or editorial or letter).pt.	2134459
48	exp Review/ or Meta-Analysis/ or "Systematic review"/	3133703
49	((systematic or state-of-the-art or scoping or literature or umbrella) adj2 (review* or overview*)).ti.	264354
50	(meta-analy* or metaanaly* or metasynthe* or meta-synthe* or "review* of reviews").ti.	156386
51	(exp Child/ or Adolescent/ or Young adult/) not exp Aged/	2923116
52	47 or 48 or 49 or 50	5290479
53	8 and 46	11255
54	53 not 52	9209
55	54 not 51	8030

2. Embase

Interface: embase.com<br><br>Date of Search: same as above<br><br>Number of hits: 7979<br><br>Comment: Emtree is the controlled vocabulary in Embase	Field labels<br><br>• /exp = exploded Emtree term<br>• /de = non exploded Emtree term<br>• ti,ab,kw = title, abstract and author keywords<br>• NEAR/x = within x words, regardless of order<br>• * = truncation of word for alternate endings

No. Query Results

#54 #50 NOT #53 7979

#53 #51 OR #52 4064475

#52 'adolescent'/de 1822165

#51 'child'/exp 3175382

#50 #45 NOT #49 9789

#49 #46 OR #47 OR #48 10214396

#48 'meta analy*':ti OR metaanaly*:ti OR metasynthe*:ti OR 'meta synthe*':ti OR 'review* of reviews':ti 192055

#47 ((systematic OR 'state of the art' OR scoping OR literature OR umbrella) NEAR/2 (review* OR overview*)):ti 310054

#46 'conference abstract'/it OR 'conference paper'/it OR 'conference review'/it OR 'editorial'/it OR 'letter'/it OR 'review'/it 10029240

#45 #8 AND #44 16116

#44 #9 OR #10 OR #11 OR #12 OR #13 OR #14 OR #15 OR #16 OR #17 OR #18 OR #19 OR #20 OR #21 OR #22 OR #23 OR #24 OR #25 OR #26 OR #27 OR #28 OR #29 OR #30 OR #31 OR #32 OR #33 OR #34 OR #35 OR #36 OR #37 OR #38 OR #39 OR #40 OR #41 OR #42 OR #43 2652531

#43 ((control OR prevent* OR risk) NEAR/2 (infection* OR infectious)):ti,ab,kw 131439

#42 abscess*:ti,ab,kw OR 'bovine respiratory disease complex':ti,ab,kw OR bronchitis:ti,ab,kw OR coinfection:ti,ab,kw OR 'common cold':ti,ab,kw OR empyema:ti,ab,kw OR 'fungal lung disease*':ti,ab,kw OR 'healthcare-associated pneumonia':ti,ab,kw OR 'human influenza':ti,ab,kw OR 'laryngeal tuberculosis':ti,ab,kw OR laryngitis:ti,ab,kw OR 'latent syphilis':ti,ab,kw OR 'latent tubercolosis':ti,ab,kw OR legionellosis:ti,ab,kw OR mycoses:ti,ab,kw OR 'parasitic lung disease*':ti,ab,kw OR pharyngitis:ti,ab,kw OR 'pleural tuberculosis':ti,ab,kw OR pleurisy:ti,ab,kw OR 'pulmonary tuberculosis':ti,ab,kw OR pyomyositis:ti,ab,kw OR rhinitis:ti,ab,kw OR rhinoscleroma:ti,ab,kw OR 'severe acute respiratory

Karolinska Institutet

syndrome':ti,ab,kw OR sinusitis:ti,ab,kw OR superinfection*:ti,ab,kw OR suppurat*:ti,ab,kw OR supraglottitis:ti,ab,kw OR 'viral bronchiolitis':ti,ab,kw OR 'viral hepatitis':ti,ab,kw OR 'viral pneumonia':ti,ab,kw OR 'whooping cough':ti,ab,kw 421034

#41 (('air borne' OR airborne OR asymptomatic OR bacterial OR 'blood borne' OR bloodborne OR coinfection OR 'co-infection communicable' OR 'community acquired' OR chronic OR cross OR hiv OR infectious OR latent OR 'long term' OR mixed OR opportunistic OR persistent OR polymicrobial OR reactivated OR 'respiratory tract' OR 'soft tissue' OR superinvasion OR transmission* OR transmitted OR secondary OR 'vaccine preventable' OR 'vaccine preventable' OR viral OR virus OR waterborne OR 'water related' OR 'water related' OR wound) NEAR/2 (diseas* OR infect*)):ti,ab,kw 1460434

#40 'wound infection'/de 52643

#39 'water borne disease'/de 815

#38 'virus pneumonia'/de 21145

#37 'virus hepatitis'/de 22122

#36 'animal hepatitis'/de 1266

#35 'dna virus infection'/de 1015

#34 'viral bronchiolitis'/de 1814

#33 'virus infection'/de 179948

#32 'vaccine preventable disease'/de 501

#31 'pyomyositis'/de 1572

#30 'empyema'/de 11632

#29 'abscess'/de 51005

#28 'suppuration'/de 3477

#27 'soft tissue infection'/de 13745

#26 'respiratory tract infection'/exp 522155

#25 'chronic infection'/de 7667

#24 'superinfection'/de 9140

#23 'chronic infection'/de 7667

#22 'aids related complex'/exp 15735

#21 'opportunistic infection'/de 28426

#20 'latent syphilis'/de 304

#19 'latent tuberculosis'/de 7099

#18	'latent infection'/de	203
#17	'health care associated pneumonia'/de	932
#16	'cross infection'/de	23142
#15	'community acquired infection'/de	4110
#14	'bloodstream infection'/de	16094
#13	'communicable disease'/de	37454
#12	'coinfection'/de	46557
#11	'bacterial infection'/de	132872
#10	'asymptomatic infection'/de	6601
#9	'infection'/de	328930
#8	#1 OR #2 OR #3 OR #4 OR #5 OR #6 OR #7	207622
#7	'district nurse patient*':ti,ab,kw	6
#6	'visiting nursing service'/exp	221
#5	'family carer$':ti,ab,kw OR 'family caregiver$':ti,ab,kw OR 'family care giver$':ti,ab,kw OR 'spouse care*':ti,ab,kw OR 'informal care*':ti,ab,kw	22133
#4	'community care':ti,ab,kw OR 'community health care*':ti,ab,kw OR 'community healthcare*':ti,ab,kw OR 'community health service$':ti,ab,kw OR 'community nurs*':ti,ab,kw OR 'community setting$':ti,ab,kw	30804
#3	((domiciliary OR home OR homemaker OR parish OR respite) NEAR/3 (care OR caregiver OR 'health care' OR healthcare OR hhc OR nurse* OR nursing OR therap* OR service* OR hemodialys* OR nutrition)):ti,ab,kw	94327
#2	'community health nursing'/exp	27797
#1	'home care'/exp	84142

4. Web of Science Core Collection

Interface: Clarivate Analytics<br><br>Editions = A&HCI , ESCI , SCI-EXPANDED , SSCI<br><br>Date of Search: same as above<br><br>Number of hits: 5,186	Field labels<br><br>• TS/Topic = title, abstract, author keywords and Keywords Plus<br>• NEAR/x = within x words, regardless of order<br>• * = truncation of word for alternate endings<br><br>Note: the *Exact search*-function was used for all the searches

#1
((TI=(((domiciliary OR home OR homemaker OR parish OR respite) NEAR/2 (care OR caregiver OR "health care" OR healthcare OR hhc OR nurse* OR nursing OR therap* OR service* OR hemodialys* OR nutrition)))) OR AB=(((domiciliary OR home OR homemaker OR parish OR respite) NEAR/2 (care OR caregiver OR "health care" OR healthcare OR hhc OR nurse* OR nursing OR therap* OR service* OR hemodialys* OR nutrition))))
71,346

#2
(TI=(("community care" OR "community health care*" OR "community health care*" OR "community health service*" OR "community nurs*" OR "community setting*"))) OR AB=(("community care" OR "community health care*" OR "community health care*" OR "community health service*" OR "community nurs*" OR "community setting*"))
18,579

#3
(TI=(("family carer$" OR "family caregiver*" OR "family care giver*" OR "spouse care*" OR "informal care*"))) OR AB=(("family carer$" OR "family caregiver*" OR "family care giver*" OR "spouse care*" OR "informal care*"))
18,308

#4
(TI=("district nurse patient*")) OR AB=("district nurse patient*")
3

#5
#1 OR #2 OR #3 OR #4
103,607

#6
(TI=(((air-borne OR airborne OR asymptomatic OR bacterial OR blood-borne OR bloodborne OR coinfection OR "co-infection communicable" OR community-acquired OR chronic OR cross OR HIV OR infectious OR latent OR "long term" OR mixed OR opportunistic OR persistent OR polymicrobial OR reactivated OR "respiratory tract" OR "soft tissue" OR superinvasion OR transmission* OR transmitted OR secondary OR vaccine-preventable OR "vaccine preventable" OR viral OR virus OR waterborne OR "water related" OR water-related OR wound) NEAR/1 (diseas* OR infect*)))) OR AB=(((air-borne OR airborne OR asymptomatic OR

bacterial OR blood-borne OR bloodborne OR coinfection OR "co-infection communicable" OR community-acquired OR chronic OR cross OR HIV OR infectious OR latent OR "long term" OR mixed OR opportunistic OR persistent OR polymicrobial OR reactivated OR "respiratory tract" OR "soft tissue" OR superinvasion OR transmission* OR transmitted OR secondary OR vaccine-preventable OR "vaccine preventable" OR viral OR virus OR waterborne OR "water related" OR water-related OR wound) NEAR/1 (diseas* OR infect*)))
979,934

#7
(TI=((abscess* OR "bovine respiratory disease complex" OR bronchitis OR coinfection OR "common cold" OR empyema OR "fungal lung disease*" OR "healthcare-associated pneumonia" OR "human influenza" OR "laryngeal tuberculosis" OR laryngitis OR "latent syphilis" OR "latent tubercolosis" OR legionellosis OR mycoses OR "parasitic lung disease*" OR pharyngitis OR "pleural tuberculosis" OR pleurisy OR "pulmonary tuberculosis" OR pyomyositis OR rhinitis OR rhinoscleroma OR "severe acute respiratory syndrome" OR sinusitis OR superinfection* OR suppurat* OR supraglottis OR "viral bronchiolitis" OR "viral hepatitis" OR "viral pneumonia" OR "whooping cough"))) OR AB=((abscess* OR "bovine respiratory disease complex" OR bronchitis OR coinfection OR "common cold" OR empyema OR "fungal lung disease*" OR "healthcare-associated pneumonia" OR "human influenza" OR "laryngeal tuberculosis" OR laryngitis OR "latent syphilis" OR "latent tubercolosis" OR legionellosis OR mycoses OR "parasitic lung disease*" OR pharyngitis OR "pleural tuberculosis" OR pleurisy OR "pulmonary tuberculosis" OR pyomyositis OR rhinitis OR rhinoscleroma OR "severe acute respiratory syndrome" OR sinusitis OR superinfection* OR suppurat* OR supraglottis OR "viral bronchiolitis" OR "viral hepatitis" OR "viral pneumonia" OR "whooping cough"))
232,730

#8
(TI=(((control OR prevent* OR risk) NEAR/1 (infection* OR infectious)))) OR AB=(((control OR prevent* OR risk) NEAR/1 (infection* OR infectious)))
84,233

#9
#6 OR #7 OR #8
1,223,065

#10
#5 AND #9
5,505

#11
(TI=((congress OR "clinical conference" OR comment OR editorial OR letter)))
477,552

#12
(TS=(((systematic OR state-of-the-art OR scoping OR literature OR umbrella) NEAR/1 (review* OR overview*))))
501,779

#13
(TI=((meta-analy* OR metaanaly* OR metasynthe* OR meta-synthe* OR "review* of reviews")))
268,359

#14
#11 OR #12 OR #13
1,011,004

#15
#10 NOT #14
5,186

Karolinska Institutet

5. Sociological Abstracts

Interface: ProQuest<br><br>Date of Search: same as above<br><br>Number of hits: 727	Field labels<br><br>• MAINSUBJECT.EXACT= subject heading<br>• TI = title<br>• AB = abstract<br>• IF = author keywords<br>• NEAR/x = within x words, regardless of order<br>• * = truncation of word for alternate endings

(((MAINSUBJECT.EXACT.EXPLODE("Home Care") OR MAINSUBJECT.EXACT.EXPLODE("Health Care Services") OR (ti((("community care" OR "community health care*" OR "community health service*" OR ("community nurse" OR "community nursery" OR "community nurses" OR "community nursing") OR ("community setting")))) OR ab((("community care" OR "community health care*" OR "community health service*" OR ("community nurse" OR "community nursery" OR "community nurses" OR "community nursing") OR ("community setting")))) OR if((("community care" OR "community health care*" OR "community health service*" OR ("community nurse" OR "community nursery" OR "community nurses" OR "community nursing") OR ("community setting"))))) OR (ti(((domiciliary OR home OR homemaker OR parish OR respite) NEAR/2 (care OR caregiver OR "health care" OR healthcare OR hhc OR nurse* OR nursing OR therap* OR service* OR hemodialys* OR nutrition))) OR ab(((domiciliary OR home OR homemaker OR parish OR respite) NEAR/2 (care OR caregiver OR "health care" OR healthcare OR hhc OR nurse* OR nursing OR therap* OR service* OR hemodialys* OR nutrition))) OR if(((domiciliary OR home OR homemaker OR parish OR respite) NEAR/2 (care OR caregiver OR "health care" OR healthcare OR hhc OR nurse* OR nursing OR therap* OR service* OR hemodialys* OR nutrition)))) OR (ti(("family carer?" OR ("family caregiver" OR "family caregivers") OR "family care giver*" OR "spouse care*" OR ("informal care" OR "informal caregivers"))) OR ab(("family carer?" OR ("family caregiver" OR "family caregivers") OR "family care giver*" OR "spouse care*" OR ("informal care" OR "informal caregivers"))) OR if(("family carer?" OR ("family caregiver" OR "family caregivers") OR "family care giver*" OR "spouse care*" OR ("informal care" OR "informal caregivers"))))) AND ((ti((((air-borne OR airborne OR asymptomatic OR bacterial OR blood-borne OR bloodborne OR coinfection OR co-infection OR communicable OR community-acquired OR chronic OR cross OR "HIV" OR infectious OR latent OR "long term" OR mixed OR opportunistic OR persistent OR polymicrobial OR reactivated OR "respiratory tract" OR "soft tissue" OR superinvasion OR transmission* OR transmitted OR secondary OR vaccine-preventable OR "vaccine preventable" OR viral OR virus OR waterborne OR "water related" OR water-related OR wound) NEAR/2 (diseas* OR infect*)))) OR ab((((air-borne OR airborne OR asymptomatic OR bacterial OR blood-borne OR bloodborne OR coinfection OR co-infection OR communicable OR community-acquired OR chronic OR cross OR "HIV" OR infectious OR latent OR "long term" OR mixed OR opportunistic OR persistent OR polymicrobial OR reactivated OR "respiratory tract" OR "soft tissue" OR superinvasion OR transmission* OR transmitted OR secondary OR vaccine-preventable OR "vaccine preventable" OR viral OR virus OR waterborne OR "water related" OR water-related OR wound) NEAR/2 (diseas* OR infect*)))) OR if((((air-borne OR airborne OR asymptomatic OR bacterial OR blood-borne OR bloodborne OR coinfection OR co-infection OR communicable OR community-acquired OR chronic OR cross OR "HIV" OR infectious OR latent OR "long term" OR mixed OR opportunistic OR persistent OR polymicrobial OR reactivated OR "respiratory tract" OR "soft tissue" OR superinvasion OR transmission* OR transmitted OR secondary OR vaccine-preventable OR "vaccine preventable" OR viral OR virus OR waterborne OR "water related" OR water-related OR wound) NEAR/2 (diseas* OR infect*))))) OR (ti(((control or prevent* or risk) near/2 (infection* or infectious))) OR ab(((control or prevent* or risk) near/2 (infection* or infectious))) OR if(((control or prevent* or risk) near/2 (infection* or infectious)))) OR (ti(((control or prevent* or risk) near/2

Karolinska Institutet

(infection* or infectious))) OR ab(((control or prevent* or risk) near/2 (infection* or infectious))) OR if(((control or prevent* or risk) near/2 (infection* or infectious)))))) NOT (MAINSUBJECT.EXACT.EXPLODE("Children") OR MAINSUBJECT.EXACT.EXPLODE("Adolescents") NOT MAINSUBJECT.EXACT.EXPLODE("Middle Aged Adults"))) NOT (ti(((systematic OR state-of-the-art OR scoping OR literature OR umbrella) NEAR/2 (review* OR overview*))) OR ti((meta-analy* OR metaanaly* OR metasynthe* OR meta-synthe* OR "review* of reviews") .ti.))

=727

6. Cinahl

Interface: Ebsco<br><br>Date of Search: same as above<br><br>Number of hits: 6161	Field labels<br><br>• MH+ = exploded Cinahl Heading<br>• MH = non exploded Cinahl Heading<br>• TI = title<br>• AB = abstract<br>• Nx = within x words, regardless of order<br>• * = truncation of word for alternate endings
S53	S52 NOT S51<br><br>6,161
S52	S40 NOT S45<br><br>6,962
S51	S49 NOT S50<br><br>1,016,399
S50	(MH "Aged+")<br><br>913,647
S49	S46 OR S47 OR S48<br><br>1,181,940
S48	(MH "Young Adult")<br><br>278,513
S47	(MH "Adolescence+")<br><br>576,732
S46	(MH "Child+")

	725,510	
S45	S41 OR S42 OR S43 OR S44	868,135
S44	TI (meta-analy* or metaanaly* or metasynthe* or meta-synthe* or "review* of reviews")	68,292
S43	(MH "Meta Analysis")	62,496
S42	(MH "Systematic Review")	109,6
S41	PT (congress or clinical conference or comment or editorial or letter)	708,433
S40	S8 AND S39	7,442
S39	S37 OR S38	397,111
S38	S34 OR S35 OR S36	287,283

S37 S9 OR S10 OR S11 OR S12 OR S13 OR S14 OR S15 OR S16 OR S17 OR S18 OR S19 OR S20 OR S21 OR S22 OR S23 OR S24 OR S25 OR S26 OR S27 OR S28 OR S29 OR S30 OR S31 OR S32 OR S33 166,943

S36 TI (((control or prevent* or risk) N2 (infection* or infectious))) OR AB (((control or prevent* or risk) N2 (infection* or infectious))) 43,834

S35 TI ((abscess* or "bovine respiratory disease complex" or bronchitis or coinfection or "common cold" or empyema or "fungal lung disease*" or "healthcare-associated pneumonia" or "human influenza" or "laryngeal tuberculosis" or laryngitis or "latent syphilis" or "latent tubercolosis" or legionellosis or mycoses or "parasitic lung disease*" or pharyngitis or "pleural tuberculosis" or pleurisy or "pulmonary tuberculosis" or pyomyositis or rhinitis or rhinoscleroma or "severe acute respiratory syndrome" or sinusitis or superinfection* or suppurat* or supraglottitis or "viral bronchiolitis" or "viral hepatitis" or "viral pneumonia" or "whooping cough")) OR AB ((abscess* or "bovine respiratory disease complex" or bronchitis or coinfection or "common cold" or empyema or "fungal lung disease*" or "healthcare-associated pneumonia" or "human influenza" or "laryngeal tuberculosis" or laryngitis or "latent syphilis" or "latent tubercolosis" or legionellosis or mycoses or "parasitic lung disease*" or pharyngitis or "pleural tuberculosis" or pleurisy or "pulmonary tuberculosis" or pyomyositis or rhinitis or rhinoscleroma or "severe acute respiratory syndrome" or sinusitis or superinfection* or suppurat* or supraglottitis or "viral bronchiolitis" or "viral hepatitis" or "viral pneumonia" or "whooping cough")) 42,548

S34 TI (((air-borne or airborne or asymptomatic or bacterial or blood-borne or bloodborne or coinfection or co-infection or communicable or community-acquired or chronic or cross or "HIV" or infectious or latent or "long term" or mixed or opportunistic or persistent or polymicrobial or reactivated or "respiratory tract" or "soft tissue" or superinvasion or transmission* or transmitted or secondary or vaccine-preventable or "vaccine preventable" or viral or virus or waterborne or "water related" or water-related or wound) N2 (diseas* or infect*))) OR AB (((air-borne or airborne or asymptomatic or bacterial or blood-borne or bloodborne or coinfection or co-infection or communicable or community-acquired or chronic or cross or "HIV" or infectious or latent or "long term" or mixed or opportunistic or persistent or polymicrobial or reactivated or "respiratory tract" or "soft tissue" or superinvasion or transmission* or transmitted or secondary or vaccine-preventable or "vaccine preventable" or viral or virus or waterborne or "water related" or water-related or wound) N2 (diseas* or infect*))) 225,719

S33 (MH "Wound Infection") Display

S32	(MH "Pneumonia, Viral")	Display
S31	(MH "Opportunistic Infections")	Display
S30	(MH "Hepatitis, Viral, Human")	Display
S29	(MH "DNA Virus Infections")	Display
S28	(MH "Virus Diseases")	Display
S27	(MH "Vaccine-Preventable Diseases")	Display
S26	(MH "Empyema")	Display
S25	(MH "Abscess")	Display
S24	(MH "Suppuration")	Display

Karolinska Institutet

S23	(MH "Soft Tissue Infections")	Display
S22	(MH "Respiratory Tract Infections+")	Display
S21	(MH "Superinfection")	Display
S20	(MH "AIDS-Related Opportunistic Infections")	Display
S19	(MH "Opportunistic Infections")	Display
S18	(MH "Latent Infection")	Display
S17	(MH "Healthcare-Associated Pneumonia")	Display
S16	(MH "Cross Infection")	Display
S15	(MH "Community-Acquired Infections")	Display
S14	(MH "Blood-Borne Infections")	Display

Karolinska Institutet

S13	(MH "Communicable Diseases, Imported")	Display
S12	(MH "Communicable Diseases")	Display
S11	(MH "Coinfection")	Display
S10	(MH "Bacterial Infections")	Display
S9	(MH "Infection")	Display
S8	S1 OR S2 OR S3 OR S4 OR S5 OR S6 OR S7	Display
S7	TI district nurse patient* OR AB district nurse patient*	Display
S6	(MH "Home Nursing")	Display
S5	TI (("family carer?" or "family caregiver*" or "family care giver*" or "spouse care*" or "informal care*")) OR AB (("family carer?" or "family caregiver*" or "family care giver*" or "spouse care*" or "informal care*"))	Display

S4 TI (("community care" or "community health care*" or "community healthcare*" or "community health service*" or "community nurs*" or "community setting*")) OR AB (("community care" or "community health care*" or "community healthcare*" or "community health service*" or "community nurs*" or "community setting*")) Display

S3 TI (((domiciliary or home or homemaker or parish or respite) N2 (care or caregiver or "health care" or healthcare or hhc or nurse* or nursing or therap* or service* or hemodialys* or nutrition))) OR AB (((domiciliary or home or homemaker or parish or respite) N2 (care or caregiver or "health care" or healthcare or hhc or nurse* or nursing or therap* or service* or hemodialys* or nutrition))) Display

S2 (MH "Community Health Nursing+") Display

S1 (MH "Home Health Care+") Display
