# Supplementary material for: Risk factors for infection in older adults with home care: a mixed methods systematic review with meta-analysis
Source: BMC Public Health. 2025 May 3;25:1643. doi: 10.1186/s12889-025-22538-1 (PMC12048934; doi:10.1186/s12889-025-22538-1)
Supplement: Supplementary file 3 — Supplementary Material 3 [file 12889_2025_22538_MOESM3_ESM.docx]

Records identified through database searching:

(n = 17,984)

**Identification**

Number of duplicates deleted

(n = 1,514)

Records after duplicates removed

(n = 16,470)

Records excluded

(n = 16,390)

Records screened through title and abstract

(n = 16,470)

Full text assessed for eligibility

(n = 80)

**Screening**

Hand searching

(n = 1)

**Studies excluded (n = 55)**

Reason: Lack of main focus on infection risk factors and/or home health care and/or old age (n=34)

Main focus on IPC (n=15)

Only agency perspective (n=1)

Not primary research (descriptive studies) (n=1)

Others (on development of a model (n=2), student thesis (n=1), implementation science focus (n=1) )

Full text assessed for eligibility

(n = 81)

Assessment of methodological quality (n = 26)

**Included**

Studies excluded following quality assessment (n=4)

Qualitative studies (n =3)

Quantitative studies (n = 1)

Studies included in review

(n = 22)

Reports of included studies

(n = 22)

Updated search

Records identified through database searching:

(n = 1500)

**Identification**

Number of duplicates deleted

(n = 0)

Records after duplicates removed

(n = 1500)

Records excluded

(n = 1431)

Records screened through title and abstract

(n = 1500)

Full text assessed for eligibility

(n = 69)

**Screening**

**Studies excluded (n = 61)**

Reason: Lack of main focus on infection risk factors and/or home health care and/or old age (n=53)

Not primary research (descriptive studies) (n=8)

Full text assessed for eligibility

(n = 69)

Studies excluded following quality assessment (n=3)

Qualitative studies (n =2)

Quantitative studies (n = 1)

Assessment of methodological quality (n = 8)

**Included**

Studies included in review

(n = 5)

Reports of included studies

(n = 5)
