# Supplementary material for: Risk factors for infection in older adults with home care: a mixed methods systematic review with meta-analysis
Source: BMC Public Health. 2025 May 3;25:1643. doi: 10.1186/s12889-025-22538-1 (PMC12048934; doi:10.1186/s12889-025-22538-1)
Supplement: Supplementary file 4 — Supplementary Material 4 [file 12889_2025_22538_MOESM4_ESM.docx]

**APPENDIX 4. STUDIES INELIGIBLE FOLLOWING FULL TEXT REVIEW**

1. Adams V, Song J, Shang J, McDonald M, Dowding D, Ojo M, et al. Infection prevention and control practices in the home environment: Examining enablers and barriers to adherence among home health care nurses. American journal of infection control. 2021;49(6):721–6.

*Reason for exclusion: Focus on infection prevention and control (IPC) practice adherence.*

1. Arnold CM. Responding to Variation in Performance Related to Wound Infection: Home Health Case Study. *Home Health Care Management & Practice*. 1999;12(1):30-35.

*Reason for exclusion: Case study on implementation of processes on wound infection*

*control practices.*

1. Bakunas-Kenneley I. Infection control practices in home healthcare. ProQuest Dissertations Publishing; 2007.

*Reason for exclusion: Doctoral thesis substituted by two related articles by same author.*

1. Baumgarten K, Hale Y, Messonnier M, McCabe M, Albright M, Bergeron E. Bridging the gap: a collaborative to reduce peripherally inserted central catheter infections in the home care environment. The Ochsner journal. 2013;13(3):352–8.

*Reason for exclusion: No main focus on risk factors for infection related to home health care (HHC).*

1. Bennett G, Mansell I. Universal precautions: a survey of community nurses’ experience and practice. Journal of clinical nursing. Submitted for publication: 20 February 2003 Accepted for publication: 30 October 2003. 2004;13(4):413–21.

*Reason for exclusion: No main focus on older age.*

1. Bi SH, Tang W, Rigodanzo-Massey N, Young BA, Blagg CR, Wang T, et al. Infection-Related Hospitalizations in Home Hemodialysis Patients. Blood purification. 2015;40(3):187–93.

*Reason for exclusion: No main focus on older age. No main focus on HHC.*

1. Bond A, Teubner A, Taylor M, Cawley C, Abraham A, Dibb M, et al. Assessing the impact of quality improvement measures on catheter related blood stream infections and catheter salvage: Experience from a national intestinal failure unit. Clinical nutrition (Edinburgh, Scotland). 2018;37(6):2097–101.

*Reason for exclusion: No main focus on older age. No main focus on HHC.*

1. Bozzetti F, Mariani L, Bertinet DB, Chiavenna G, Crose N, De Cicco M, et al. Central venous catheter complications in 447 patients on home parenteral nutrition: an analysis of over 100.000 catheter days. Clinical nutrition (Edinburgh, Scotland). 2002;21(6):475–85.

*Reason for exclusion: No main focus on older age. No main focus on HHC.*

1. Buchman AL, Opilla M, Kwasny M, Diamantidis TG, Okamoto R. Risk Factors for the Development of Catheter-Related Bloodstream Infections in Patients Receiving Home Parenteral Nutrition. JPEN Journal of parenteral and enteral nutrition. 2014;38(6):744–9.

*Reason for exclusion: No main focus on older age. No main focus on HHC.*

1. Chenoweth CE, Washer LL, Obeyesekera K, Friedman C, Brewer K, Fugitt GE, Lark R. Ventilator-associated pneumonia in the home care setting. Infect Control Hosp Epidemiol. 2007 Aug;28(8):910-5. doi: 10.1086/519179. Epub 2007 Jun 14. PMID: 17620236.

*Reason for exclusion: No main focus on older age.*

1. Corrêa Cordeiro JF, Pavinski Alves A, Gir E, Oliveira Miranda D, Marin da Silva Canini SR. Use of Personal Protective Equipment in a Home Care Service. Cogitare Enfermagem [Internet]. 2016 Jul [cited 2022 Aug 31];21(3):01–8.

*Reason for exclusion: Focus on IPC practice adherence.*

1. Dacey GT, Dufficy L. Unknown phenomena: a study of infection control practices in the community setting. Australian infection control. 1998;3(4):16–21.

*Reason for exclusion: No main focus on HHC.*

1. de Figueiredo RM de, Maroldi MAC. Home care: health professionals at risk for biological exposure. Revista da Escola de Enfermagem da U S P. 2012;46(1):145–.

*Reason for exclusion: Focus on IPC practice adherence.*

1. Do AN, Ray BJ, Banerjee SN, Illian AF, Barnett BJ, Pham MH, et al. Bloodstream Infection Associated with Needleless Device Use and the Importance of Infection-Control Practices in the Home Health Care Setting. The Journal of infectious diseases. 1999;179(2):442–8.

*Reason for exclusion: No main focus on older age. No main focus on HHC related risk factors.*

1. Hamaguchi S, Suzuki M, Sasaki K, Abe M, Wakabayashi T, Sando E, et al. Six underlying health conditions strongly influence mortality based on pneumonia severity in an ageing population of Japan: a prospective cohort study. BMC pulmonary medicine. 2018;18(1):88–88.

*Reason for exclusion: No main focus on HHC.*

1. Hancock RD. Qualitative Analysis of Older Adults’ Experiences with Sepsis. ProQuest Dissertations Publishing; 2018.

*Reason for exclusion: No focus on HHC.*

1. Keller SC, Wang NY, Salinas A, Williams D, Townsend J, Cosgrove SE. Which Patients Discharged to Home-Based Outpatient Parenteral Antimicrobial Therapy Are at High Risk of Adverse Outcomes? Open forum infectious diseases. 2020;7(6):ofaa178–ofaa178.

*Reason for exclusion: No main focus on older age. No main focus on infection. No main focus on HHC.*

1. Keller SC, Cosgrove SE, Kohut M, Krosche A, Chang HE, Williams D, et al. Hazards from physical attributes of the home environment among patients on outpatient parenteral antimicrobial therapy. American journal of infection control. 2019;47(4):425–30.

*Reason for exclusion: No main focus on older age. No main focus on HHC and related infection risk factors.*

1. Kenneley IL. Infection Control and the Home Care Environment. Home health care management & practice. 2010;22(3):195–201.

*Reason for exclusion: Focus on IPC practice adherence.*

1. Kenneley I. Infection control in home healthcare: an exploratory study of issues for patients and providers. Home healthcare nurse. 2012;30(4):235–45.

*Reason for exclusion: Focus on IPC practice adherence.*

1. Kiss IZ, Blyuss KB, Kyrychko YN, Middleton J, Roland D, Bertini L, et al. How can risk of COVID-19 transmission be minimised in domiciliary care for older people: development, parameterisation and initial results of a simple mathematical model. Epidemiology and infection. 2022;150.

*Reason for exclusion: Study design not in accordance with PICO.*

1. Landes SD, Turk MA, Damiani MR, Proctor P, Baier S. Risk Factors Associated With COVID-19 Outcomes Among People With Intellectual and Developmental Disabilities Receiving Residential Services. JAMA network open. 2021;4(6):e2112862–e2112862.

*Reason for exclusion: No main focus on HHC.*

1. LeClair SM, Schicker JM, Duthie EH, Hoffmann RG, Franson TR. Survey of nursing personnel attitudes toward infections and their control in the elderly. American journal of infection control. 1988;16(4):159–66.

*Reason for exclusion: Focus on IPC practice adherence.*

1. Leiss JK, Sitzman KL, Kendra MA. Provision and use of personal protective equipment among home care and hospice nurses in North Carolina. American journal of infection control. 2011;39(2):123–8.

*Reason for exclusion: Focus on IPC practice adherence.*

1. Lescure FX, Locher G, Eveillard M, Biendo M, Van Agt S, Le Loup G, Douadi Y, Ganry O, Vandenesch F, Eb F, Schmit JL, Etienne J. community-acquired infection with healthcare-associated methicillin-resistant Staphylococcus aureus: the role of home nursing care. Infect Control Hosp Epidemiol. 2006 Nov;27(11):1213-8. doi: 10.1086/507920. Epub 2006 Oct 20. PMID: 17080379.

*Reason for exclusion: No main focus on HHC. No main focus on older age.*

1. Lim CJ, Cheng AC, Kong DCM, Peleg AY. Community-onset bloodstream infection with multidrug-resistant organisms: a matched case-control study. BMC infectious diseases. 2014;14(1):126–126.

*Reason for exclusion: No main focus on HHC.*

1. Lindh M, Kihlgren A, Perseius KI. Factors influencing compliance to hygiene routines in community care - the viewpoint of medically responsible nurses in Sweden. Scandinavian journal of caring sciences. Submitted 5 April 2012, Accepted 17 April 2012. 2013;27(2):224–30.

*Reason for exclusion: Regards only agency-level perspective.*

1. Marin-Gomez FX, Mendioroz-Peña J, Mayer MA, Méndez-Boo L, Mora N, Hermosilla E, et al. Comparing the Clinical Characteristics and Mortality of Residential and Non-Residential Older People with COVID-19: Retrospective Observational Study. International journal of environmental research and public health. 2022;19(1):483–.

*Reason for exclusion: No main focus on HHC.*

1. Morioka N, Kashiwagi M, Hamano J. Adherence to Personal Protective Equipment Use in Home-Care Service Agencies During COVID-19 in Japan: A Cross-Sectional Survey. Journal of the American Medical Directors Association. 2022;23(6):930–935.e2.

*Reason for exclusion: Focus on IPC practice adherence.*

1. Nakano K, Ono K, Yasumura S. [A survey of infection control among community home care service providers]. Nihon Koshu Eisei Zasshi. 2002 Dec;49(12):1239-49. Japanese. PMID: 12607988.

*Reason for exclusion: Focus on IPC practice adherence.*

1. Naseer M, J McKee K, Ehrenberg A, Schön P, Dahlberg L. Individual and contextual predictors of emergency department visits among community-living older adults: a register-based prospective cohort study. BMJ open. 2022;12(2):e055484–e055484.

*Reason for exclusion: No focus on infection. No main focus on HHC.*

1. Nilsson L, Andersson C, Sjodahl R. COVID-19 as the sole cause of death is uncommon in frail home healthcare individuals: a population-based study. BMC geriatrics. 2021;21(1):262–262.

*Reason for exclusion: No main focus on HHC.*

1. Norrie C, Woolham J, Samsi K, Manthorpe J. Personal Assistants’ role in infection prevention and control: Their experiences during the Covid‐19 pandemic. Health & social care in the community. 2022;30(5):e1926–e1934.

*Reason for exclusion: No main focus on older age.*

1. Olufon O, Iyanger N, Cleary V, Lamagni T. An outbreak of invasive group A streptococcal infection among elderly patients receiving care from a district nursing team, October 2013 – May 2014. Journal of infection prevention. 2015;16(4):174–7.

*Reason for exclusion: Not primary research.*

1. Pini SF, Sgaramella GA, Parra Jordán JJ, Aguilera Zubizarreta A, Pariente Rodrigo E, Sanroma Mendizabal P. Factors associated to Clostridium difficile infection in a hospital-based home care service. Medicina clínica (English ed). 2019;153(8):319–22.

*Reason for exclusion: No main focus on HHC.*

1. Popp W, Hilgenhöner M, Dogru-Wiegand S, Hansen D, Daniels-Haardt I. Hygiene in home care. A study with home care providers. Bundesgesundheitsblatt, Gesundheitsforschung, Gesundheitsschutz. 2006;49(12):1195–.

*Reason for exclusion: Focus on IPC practice adherence.*

1. Reimund JM, Arondel Y, Finck G, Zimmermann F, Duclos B, Baumann R. Catheter-related infection in patients on home parenteral nutrition: results of a prospective survey. Clinical nutrition (Edinburgh, Scotland). 2002;21(1):33–8.

*Reason for exclusion: No main focus on older age. No main focus on HHC.*

1. Ross VM, Guenter P, Corrigan ML, Kovacevich D, Winkler MF, Resnick HE, et al. Central venous catheter infections in home parenteral nutrition patients: Outcomes from Sustain: American Society for Parenteral and Enteral Nutrition’s National Patient Registry for Nutrition Care. American journal of infection control. 2016;44(12):1462–8.

*Reason for exclusion: No main focus on older age. No main focus on HHC.*

1. Rowe TA, Patel M, O’Conor R, McMackin S, Hoak V, Lindquist LA. COVID-19 exposures and infection control among home care agencies. Archives of gerontology and geriatrics. 2020;91:104214–104214.

*Reason for exclusion: Focus on IPC practice. Contains only agency perspective.*

1. Russell D, Dowding DW, McDonald MV, Adams V, Rosati RJ, Larson EL, et al. Factors for compliance with infection control practices in home healthcare: findings from a survey of nurses’ knowledge and attitudes toward infection control. American journal of infection control. 2018;46(11):1211–7.

*Reason for exclusion: Focus on IPC practice adherence.*

1. Santarpia L, Buonomo A, Pagano MC, Alfonsi L, Foggia M, Mottola M, et al. Central venous catheter related bloodstream infections in adult patients on home parenteral nutrition: Prevalence, predictive factors, therapeutic outcome. Clinical nutrition (Edinburgh, Scotland). 2016;35(6):1394–8.

*Reason for exclusion: No main focus on older age. No main focus on HHC.*

1. Saqui O, Fernandes G, Allard J. Central venous catheter infection in Canadian home parenteral nutrition patients: a 5-year multicenter retrospective study. British journal of nursing (Mark Allen Publishing). 2020;29(8):S34–S42.

*Reason for exclusion: No main focus on older age. No main focus on HHC.*

1. Schildmeijer KGI, Unbeck M, Ekstedt M, Lindblad M, Nilsson L. Adverse events in patients in home healthcare: a retrospective record review using trigger tool methodology. BMJ open. 2018;8(1):e019267–e019267.

*Reason for exclusion: Does not include risk factors of infection.*

1. Shang J, Chastain AM, Perera UGE, Dick AW, Fu CJ, Madigan EA, et al. The state of infection prevention and control at home health agencies in the United States prior to COVID-19: A cross-sectional study. International journal of nursing studies. 2021;115:103841–103841.

*Reason for exclusion: Focus on IPC practice adherence.*

1. Shirotani N, Iino T, Numata K, Kameoka S. Complications of central venous catheters in patients on home parenteral nutrition: an analysis of 68 patients over 16 years. Surgery today (Tokyo, Japan). 2006;36(5):420–4.

*Reason for exclusion: No main focus on older age. No main focus on HHC.*

1. Song, Jiyoun PhD, RN, AGACNP-BC; Woo, Kyungmi PhD, RN; Shang, Jingjing PhD, RN; Ojo, Marietta MPH; Topaz, Maxim PhD, RN. Predictive Risk Models for Wound Infection-Related Hospitalization or ED Visits in Home Health Care Using Machine-Learning Algorithms. Advances in Skin & Wound Care: August 2021 - Volume 34 - Issue 8 - p 1-12.

*Reason for exclusion: Focus on a machine learning algorithm, where the wound infections in HHC happen to be the topic.*

1. Steffens E, Spriet I, Van Eldere J, Schuermans A. Compliance with evidence-based guidelines for the prevention of central line–associated bloodstream infections in a Belgian home care setting: An observational study. American journal of infection control. 2019;47(6):723–5.

*Reason for exclusion: Focus on IPC practice adherence.*

1. Tokars JI, Cookson ST, McArthur MA, Boyer CL, McGeer AJ, Jarvis WR. Prospective evaluation of risk factors for Bloodstream infection in Patients receiving home infusion therapy. Annals of internal medicine. 1999;131(5):340–7.

*Reason for exclusion: No main focus on older age. No main focus on HHC.*

1. Toledo D, Soldevila N, Torner N, Pérez-Lozano MJ, Espejo E, Navarro G, et al. Factors associated with 30-day readmission after hospitalisation for community-acquired pneumonia in older patients: a cross-sectional study in seven Spanish regions. BMJ open. 2018;8(3):e020243–e020243.

*Reason for exclusion: No main focus on HHC.*

1. Tsilimingras D, Zhang L, Chukmaitov A. Postdischarge Adverse Events Among Patients Who Received Home Health Care Services. Home health care management & practice. 2019;31(4):257–62.

*Reason for exclusion: No main focus on infection risk factors.*

1. White M. C. (1992). Infections and infection risks in home care settings. *Infection control and hospital epidemiology*, *13*(9), 535–539.

*Reason for exclusion*: Af*ter methodological assessment, too many elements were*

*unclear or not reported, as well as unclear data were presented. To be more specific,*

*data are little on recruitment criteria, on data collection (who completed the charts)*

*and no information is given on confounders and their adjustment (if any). Lastly, data*

*in Table 4 are non-completely coherent: the total number of wound and decubitus*

*infections do not coincide with the once that occurred before HC period and the ones*

*during HC period, which summed, should be equal to the total.*

1. White MC, Smith W. Infection control in home care agencies. American journal of infection control. 1993;21(3):146–50.

*Reason for exclusion: Focus on IPC practice adherence.*

1. Woo K, Song J, Adams V, Block LJ, Currie LM, Shang J, et al. Exploring prevalence of wound infections and related patient characteristics in homecare using natural language processing. International wound journal. 2022;19(1):211–21.

*Reason for exclusion: No focus on infection risk in HHC per se.*

1. Xue Z, Coughlin R, Amorosa V, Quinn R, Schiavone P, Stoner N, et al. Factors Associated With Central Line–Associated Bloodstream Infections in a Cohort of Adult Home Parenteral Nutrition Patients. JPEN Journal of parenteral and enteral nutrition. 2020;44(8):1388–96.

*Reason for exclusion: No main focus on older adults. No main focus on HHC.*

1. Zhan T, Goyal D, Guttag J, Mehta R, Elahi Z, Syed Z, et al. Machine Intelligence for Early Targeted Precision Management and Response to Outbreaks of Respiratory Infections. The American journal of managed care. 2020;26(10):445–8.

*Reason for exclusion: No main focus on HHC.*

1. Zhao VM, Griffith DP, Blumberg HM, Dave NJ, Battey CH, McNally TA, et al. Characterization of post-hospital infections in adults requiring home parenteral nutrition. Nutrition (Burbank, Los Angeles County, Calif). 2013;29(1):52–9.

*Reason for exclusion: No main focus on older age. No main focus on HHC.*

*FROM UPDATED SEARCH*

1. Allard, and Conroy, C. A. (2022). Our Nursing Profession at a Crossroads: Time to Chart a Course for the Future. Nursing Administration Quarterly, 46(3), 208–217. https://doi.org/10.1097/NAQ.0000000000000536

*Reason for exclusion: not original research (discussion/opinion paper)*

1. Allel, K., Goscé, L., Araos, R., Toro, D., Ferreccio, C., Munita, J. M., Undurraga, E. A., & Panovska-Griffiths, J. (2022). Transmission of gram-negative antibiotic-resistant bacteria following differing exposure to antibiotic-resistance reservoirs in a rural community: a modelling study for bloodstream infections. Scientific Reports, 12(1), 13488–13488.

*Reason for exclusion: No main focus on home care.*

1. Almramhi, K., Aljehani, M., Bamuflih, M., Alghamdi, S. ., Banser, S. ., Almousa, A. ., ALABDULWAHAB, S. ., & Al-Ebrahim, K. . (2022). Frequency and Risk Factors of Unplanned 30-Day Readmission After Open Heart Surgeries: A Retrospective Study in a Tertiary Care Center. *The Heart Surgery Forum*, *25*(4), E608-E615.

*Reason for exclusion: No main focus on*

1. Amanya, Elyanu, P., Migisha, R., Kadobera, D., Ario, A. R., and Harris, J. R. (2022). Individual and household risk factors for COVID-19 infection among household members of COVID-19 patients in home-based care in western Uganda, 2020. IJID Regions, 5, 183–190. <https://doi.org/10.1016/j.ijregi.2022.11.002>

*Reason for exclusion: No main focus on old age, no main focus on home care*

1. Bern-Klug, and Bergen-Jackson, K. (2022). Trusting Relationships in a Retirement Community: How Swift Actions Likely Saved Lives. Journal of Gerontological Nursing, 48(10), 54–56. <https://doi.org/10.3928/00989134-20220909-02>

*Reason for exclusion: not original research (description of safety measures at a retirement residence)*

1. Biguenet, A., Bouxom, H., Bertrand, X., & Slekovec, C. (2023). Antibiotic resistance in elderly patients: Comparison of Enterobacterales causing urinary tract infections between community, nursing homes and hospital settings. *Infectious Diseases Now (Online)*, *53*(1), 104640–104640

*Reason for exclusion: No main focus on infection risk factors.*

1. Caldwell, J., Heyman, M., Atkins, M., & Ho, S. (2022). Experiences of individuals self-directing Medicaid Home and Community-Based Services during COVID-19. *Disability and Health Journal*, *15*(3), 101313–101313.

*Reason for exclusion: Not main focus on old age.*

1. Caughey, G. E., Lang, C. E., Bray, S. C. E., Sluggett, J. K., Whitehead, C., Visvanathan, R., Evans, K., Corlis, M., Cornell, V., Barker, A. L., Wesselingh, S., & Inacio, M. C. (2022). Quality and safety indicators for home care recipients in Australia: development and cross-sectional analyses. *BMJ Open*, *12*(8), e063152–.

*Reason for exclusion: No main focus on infection risk factors*

1. Cioffi, A. A., Cecannecchia, C. C., Baldari, B. B., & Karaboue, M. A. A. M. A. A. (2023). Informal caregivers in Italy: the “phantom zone” of welfare. *Acta Bio-Medica de l’Ateneo Parmense*, *94*(1)

*Reason for exclusion:* *not original research (policy proposal)*

1. Díaz-Gómez, Castillo-Gallego, C., Cruz-Santaella, A., Gómez-Gómez, M. D. C., Ceiro, M. D.-D., and Gómez-Rey, M. C. (2023). Efficacy of the Home Continuity Care Unit in Toledo, Spain for Older Adults with Advanced Chronic Diseases: Avoidance of Hospital Visits and Reduction of Health Cost. Home Healthcare Now, 41(1), 14–19. https://doi.org/10.1097/NHH.0000000000001125

*Reason for exclusion: No main focus on infection risk factors*

1. Edwards, S. T., Greene, L., Chaudhary, C., Boothroyd, D., Kinosian, B., & Zulman, D. M. (2022). Outpatient Care Fragmentation and Acute Care Utilization in Veterans Affairs Home-Based Primary Care. *JAMA Network Open*, *5*(9), e2230036–e2230036

*Reason for exclusion: No main focus on infection on infection risk per se.*

1. Elliott, Burt, S., and Lahr, J. (2023). Walking into a COVID Petri Dish: Home Care Providers’ Experiences during a Pandemic. Home Healthcare Now, 41(1), 20–27. <https://doi.org/10.1097/NHH.0000000000001141>

*Reason for exclusion: No main focus on old age*

1. Fang, J.-T., Chen, S.-Y., Yang, L.-Y., Liao, K.-C., Lin, C.-H., Fujimori, M., & Tang, W.-R. (2022). Improving transitional care through online communication skills training. *Aging Clinical and Experimental Research*, *34*(12), 3063–3071

*Reason for exclusion: No main focus on infection risk factors*

1. Fischer, T. (2023). Home care in Germany during the COVID‐19 pandemic: A neglected population? Journal of Nursing Scholarship, 55(1), 215–225. <https://doi.org/10.1111/jnu.12851>

*Reason for exclusion: not original research (review paper)*

1. Flemons, McGhan, G., and McCaughey, D. (2022). Family Caregiving for People Living With Dementia During COVID-19: A Thematic Analysis. Journal of Family Nursing, 28(3), 219–230. <https://doi.org/10.1177/10748407221100553>

*Reason for exclusion: No main focus on infection risks per se*

1. Franzosa, E., Wyte-Lake, T., Tsui, E. K., Reckrey, J. M., & Sterling, M. R. (2022). Essential but Excluded: Building Disaster Preparedness Capacity for Home Health Care Workers and Home Care Agencies. *Journal of the American Medical Directors Association*, *23*(12), 1990–1996.

Reason for exclusion: *not original research (review and policy proposal)*

1. Guenther, E. D., Sherman, K. A., Dysart, C. E., Haque, J., Mahatme, S., & Gundacker, N. D. (2022). Understanding the relationship between patient characteristics and complication in veterans discharged on parenteral antibiotic therapy. *Research in Social and Administrative Pharmacy*, *18*(10), 3864–3866.

*Reason for exclusion: No main focus on home care, no main focus on infection risks.*

1. Griffith, K. N., Schwartzman, D. A., Pizer, S. D., Bor, J., Kolachalama, V. B., Jack, B., & Garrido, M. M. (2022). Local Supply Of Postdischarge Care Options Tied To Hospital Readmission Rates: Study examines supply of postdischarge care options’ association with hospital readmission rates. *Health Affairs*, *41*(7), 1036–1044.

*Reason for exclusion: No main focus on infection risk factors*

1. Hapsari, Ho, J. W., Meaney, C., Avery, L., Hassen, N., Jetha, A., Lay, A. M., Rotondi, M., Zuberi, D., and Pinto, A. (2022). The working conditions for personal support workers in the Greater Toronto Area during the COVID-19 pandemic: a mixed-methods study. Canadian Journal of Public Health, 113(6), 817–833. <https://doi.org/10.17269/s41997-022-00643-7>

*Reason for exclusion: No main focus on infection risk factors*

1. Hopwood, P., Maceachen, E., Mcainey. C., Tong, C. 2022a Personal support work and home care in Ontario during the covid-19 pandemic. *Healthcare Policy* vol.18 no.2, 61-75

*Reason for exclusion: No main focus on infection risk factors*

1. Hopwood, and MacEachen, E. (2022b). Policy and Practice Note: Policy, Safety, and Regulation with Regard to Ontario Home Care Clients and Personal Support Workers. Canadian Journal on Aging, 41(3), 490–498. https://doi.org/10.1017/S0714980821000209

*Reason for exclusion: not original research (discussion/opinion paper)*

1. Huang, J., Gu, A., Li, N., He, Y., Xie, W., Fang, W., Yuan, J., & Jiang, N. (2022). Self-care or assisted PD: development of a new approach to evaluate manual peritoneal dialysis practice ability. *Renal Failure*, *44*(1), 1320–1326.

*Reason for exclusion: No main focus on infection risks per se*

1. Inloes, Brown, A., Rettell, Z., Fick, D. M., and Bell, S. A. (2023). Home-Based Care Provider Perspectives on Care Refusal During the COVID-19 Pandemic. Journal of Gerontological Nursing, 49(1), 35–41. https://doi.org/10.3928/00989134-20221206-02

*Reason for exclusion: No main focus on infection risk factors*

1. Iwai-Saito, Sato, K., and Kondo, K. (2023). Associations of influenza and pneumococcal vaccinations with burdens of older family caregivers: The Japan Gerontological Evaluation study (JAGES) cross-sectional study. Vaccine, 41(2), 444–451. <https://doi.org/10.1016/j.vaccine.2022.11.047>

*Reason for exclusion: No main focus on infection risk per se*

1. Kaye, and Caldwell, J. (2023). Excess Deaths Of Medicaid Home And Community-Based Services Recipients During COVID-19. Health Affairs, 42(1), 115–120. https://doi.org/10.1377/hlthaff.2022.00457

*Reason for exclusion: No main focus on infection risk factors*

1. Kazawa, Kubo, T., Ohge, H., and Ishii, S. (2022). Efficacy of care manager-led support for family caregivers of people with dementia during the COVID-19 pandemic: a randomized controlled study. BMC Geriatrics, 22(1), 1–671. <https://doi.org/10.1186/s12877-022-03371-2>

*Reason for exclusion: No main focus on infection per se*

1. King, E. C., Zagrodney, K. A. P., McKay, S. M., Holness, D. L., & Nichol, K. A. (2023). Determinants of nurse’s and personal support worker’s adherence to facial protective equipment in a community setting during the COVID-19 pandemic in Ontario, Canada: A pilot study. *American Journal of Infection Control*, *51*(5), 490–497.

*Reason for exclusion: No main focus on infection risk factors*

1. Ko SQ, Goh J, Tay YK, Nashi N, Hooi BMY, Luo N, Kuan WS, Soong JTY, Chan D, Lai YF, Lim YW. Treating acutely ill patients at home: Data from Singapore. Ann Acad Med Singap. 2022 Jul;51(7):392-399. doi: 10.47102/annals-acadmedsg.2021465. PMID: 35906938.

*Reason for exclusion: No main focus on infection risk factors*

1. Lanz, P., Wieczorek, M., Sadlon, A., de Godoi Rezende Costa Molino, C., Abderhalden, L. A., Schaer, D. J., Spahn, D. R., Freystätter, G., Orav, E. J., Egli, A., Bischoff-Ferrari, H. A., Rival, S., Guyonnet, S., Biver, E., Merminod, F., Bridenbaugh, S., Suhm, N., Duarte, C. C. ., Pinto, A. F., … Felson, D. T. (2022). Iron Deficiency and Incident Infections among Community-Dwelling Adults Age 70 Years and Older: Results from the DO-HEALTH Study. *The Journal of Nutrition, Health & Aging*, *26*(9), 864–871.

*Reason for exclusion: No main focus on home care*

1. Lee, Kim, H. J., Ju, E., Guo, Y., Rousseau, J., Gibbs, L., Tran, T. M., Tom, C. E., Sabino-Laughlin, E., and Kehoe, P. (2022). A Culturally and Linguistically Appropriate Telephone Support Intervention for Diverse Family Caregivers of Persons With Dementia During the COVID-19 Pandemic. Journal of Family Nursing, 28(3), 231–242. <https://doi.org/10.1177/10748407221106531>

*Reason for exclusion: No main focus on infection risks*

1. Lee, Y. J., Johnston, D. M., Reuland, M., Lyketsos, C. G., Samus, Q., & Amjad, H. (2022). Reasons for Hospitalization while Receiving Dementia Care Coordination through Maximizing Independence at Home. *Journal of the American Medical Directors Association*, *23*(9), 1573–1578.e2.

*Reason for exclusion: No main focus on risk factors in the home care setting*

1. Lopez-Doriga Ruiz, Gunnes, N., Michael Gran, J., Karlstad, Ø., Selmer, R., Dahl, J., Bøås, H., Aubrey White, R., Christine Hofman, A., Hessevik Paulsen, T., Viksmoen Watle, S., Hylen Ranhoff, A., Bukholm, G., Løvdal Gulseth, H., and Tapia, G. (2023). Short-term safety of COVID-19 mRNA vaccines with respect to all-cause mortality in the older population in Norway. Vaccine, 41(2), 323–332. <https://doi.org/10.1016/j.vaccine.2022.10.085>

*Reason for exclusion: No main focus on infection risk factors*

1. Mesa‐Melgarejo, L., Carreño Moreno, S., Chaparro‐Diaz, L., Quintero González, L. A., Garcia‐Quintero, D., Carrillo‐Algarra, A. J., Castiblanco‐Montañez, R. A., & Hernandez‐Zambrano, S. M. (2022). Effectiveness of a case management model for people with multimorbidity: Mixed methods study. Journal of Advanced Nursing, 78(11), 3830–3846

*Reason for exclusion: No main focus on infection risk factors*

1. Meric, CS., Yabanci Ayhan, N., Yilmaz, HÖ. 2022. Assessment of Nutritional Status of Elderly Receiving Home Health Care. *Aging Medicine and Healthcare* 2022;13(2):65-71. doi:10.33879/AMH.132.2021.04025

*Reason for exclusion: No main focus on infection risk factors*

1. Nakamura, Sasaki, J., Asari, Y., Sato, T., Torii, S., and Watanabe, M. (2017). Complications after implantation of subcutaneous central venous ports (PowerPortⓇ ). Annals of Medicine and Surgery, 17, 1–6. <https://doi.org/10.1016/j.amsu.2017.03.014>

*Reason for exclusion: No main focus on home care*

1. Newton, Signal, T., and Judd, J. A. (2022). Fur, Fin, and Feather: Management of Animal Interactions in Australian Residential Aged Care Facilities. Animals (Basel), 12(24), 3591–. <https://doi.org/10.3390/ani12243591>

*Reason for exclusion: No main focus on home care*

1. Nilsson, L., Lindblad, M., Johansson, N., Säfström, L., Schildmeijer, K., Ekstedt, M., & Unbeck, M. (2023). Exploring nursing-sensitive events in home healthcare: A national multicenter cohort study using a trigger tool. *International Journal of Nursing Studies*, *138*, 104434–104434.

*Reason for exclusion: No main focus on infection risk factors.*

1. Oladapo-Shittu, Hannum, S. M., Salinas, A. B., Weems, K., Marsteller, J., Gurses, A. P., Cosgrove, S. E., and Keller, S. C. (2023). The need to expand the infection prevention workforce in home infusion therapy. American Journal of Infection Control, 51(5), 594–596. <https://doi.org/10.1016/j.ajic.2022.11.008>

*Reason for exclusion: No main focus on infection risk factors*

1. Osei-Poku, GK., Szczerepa, O., Potter, AA., Malone, ME., Fain, BA., Prentice JC. (2021). Safety Trade-Offs in Home Care During CO. *Patient Safety*, Vol. 3 No. 3, 7-17.

*Reason for exclusion: No main focus on elderly*

1. Paulson, Shulman, E. P., Dunn, A. N., Fazio, J. R., Habermann, E. B., Matcha, G. V., McCoy, R. G., Pagan, R. J., and Maniaci, M. J. (2023). Implementation of a virtual and in-person hybrid hospital-at-home model in two geographically separate regions utilizing a single command center: a descriptive cohort study. BMC Health Services Research, 23(1), 139–139. <https://doi.org/10.1186/s12913-023-09144-w>

*Reason for exclusion: No main focus on infection risk in home care*

1. Petry, Pelzelmayer, K., Ernst, J., Thuerlimann, E., and Naef, R. (2023). Nurse-patient interaction during the Covid-19 pandemic: Creating and maintaining an interactive space for care. Journal of Advanced Nursing, 79(1), 281–296. https://doi.org/10.1111/jan.15486

*Reason for exclusion: No main focus on infection risk factors*

1. Razzaghi, H., Srivastav, A., de Perio, M. A., Laney, A. S., & Black, C. L. (2022). Influenza and COVID-19 Vaccination Coverage Among Health Care Personnel — United States, 2021–22. *MMWR. Morbidity and Mortality Weekly Report*, *71*(42), 1319–1326.

*Reason for exclusion: No main focus on risk factors in home care*

1. Ree E, Wiig S, Seljemo C, Wibe T, Lyng HB. Managers' strategies in handling the COVID-19 pandemic in Norwegian nursing homes and homecare services. Leadersh Health Serv (Bradf Engl). 2022 Nov 29;ahead-of-print(ahead-of-print). doi: 10.1108/LHS-05-2022-0052. PMID: 36448830.

*Reason for exclusion: No main focus on infection risk factors*

1. Reckrey, J. M., Kim, P. S., Zhao, D., Zhang, M., Xu, E., Franzosa, E., & Ornstein, K. A. (2022). Care disruptions among the homebound during the COVID‐19 pandemic: An analysis of the role of dementia. *Journal of the American Geriatrics Society (JAGS)*, *70*(12), 3585–3592.

*Reason for exclusion: No main focus on infection risk factors*

1. Rich-Edwards, J. W., Rocheleau, C. M., Ding, M., Hankins, J. A., Katuska, L. M., Kumph, X., Steege, A. L., Boiano, J. M., & Lawson, C. C. (2022). COVID-19 Vaccine Uptake and Factors Affecting Hesitancy Among US Nurses, March-June 2021. *American Journal of Public Health (1971)*, *112*(11), 1620–1629.

*Reason for exclusion: No main focus on home care*

1. Ris, I., Volken, T., Schnepp, W., & Mahrer-Imhof, R. (2022). Exploring Factors Associated With Family Caregivers’ Preparedness to Care for an Older Family Member Together With Home Care Nurses: An Analysis in a Swiss Urban Area. *Journal of Primary Care & Community Health*, *13*, 21501319221103961–21501319221103961.

*Reason for exclusion: No main focus on home care, no main focus on infection risks.*

1. Rodrigues, Teixeira-Lemos, E., Mascarenhas-Melo, F., Lemos, L. P., and Bell, V. (2022). Pharmacist Intervention in Portuguese Older Adult Care. Healthcare (Basel), 10(10), 1833–. https://doi.org/10.3390/healthcare10101833Rodrigues

*Reason for exclusion: not original research (review paper)*

1. Robinson, E. S., Cyarto, E., Ogrin, R., Green, M., & Lowthian, J. A. (2022). Quality of life of older Australians receiving home nursing services for complex care needs. *Health & Social Care in the Community*, *30*(6), e6091–e6101.

*Reason for exclusion: No main focus on infection risk factors.*

1. Rosengren, Lundberg, C. E., Söderberg, M., Santosa, A., Edqvist, J., Lindgren, M., Åberg, M., Gisslén, M., Robertson, J., Cronie, O., Sattar, N., Lagergren, J., Brandén, M., Björk, J., and Adiels, M. (2022). Severe COVID‐19 in people 55 and older during the first year of the pandemic in Sweden. Journal of Internal Medicine, 292(4), 641–653. <https://doi.org/10.1111/joim.13522>

*Reason for exclusion: No main focus on infection risks within home care*

1. Russell, D., Burgdorf, J. G., Washington, K. T., Schmitz, J., & Bowles, K. H. (2023). “Second set of eyes:” Family caregivers and post-acute home health care during the COVID-19 pandemic. *Patient Education and Counseling*, *109*, 107627–107627.

*Reason for exclusion: No main focus on infection risk factors.*

1. Ryu, Chun, J. Y., Lee, S., Yoo, D., Kim, Y., Ali, S. T., and Chun, B. C. (2022). Epidemiology and Transmission Dynamics of Infectious Diseases and Control Measures. *Viruses*, *14*(11), 2510–. <https://doi.org/10.3390/v14112510>

*Reason for exclusion: No main focus on infection risk factors in home care*

1. Sakamoto, R., Yoshida, M., Bhandari, D., Miyatake, H., Kosaka, M., Tanimoto, T., Kami, M., & Ozaki, A. (2022). Difficulty in continuing home care after informal caregiver was exposed to the COVID‐19: A case report. *Clinical Case Reports*, *10*(7).
   *Reason for exclusion: No main focus on infection risk.*
2. Saragosa, M., Jeffs, L., Okrainec, K., & Kuluski, K. (2022). Towards defining quality in home care for persons living with dementia. *PloS One*, *17*(9), e0274269–e0274269.

*Reason for exclusion: No main focus on infection risk factors.*

1. Singh, G. (2022). Filling the gaps: the home dialysis discussion. *Journal of Nephrology*, *35*(5), 1547–1547. https://doi.org/10.1007/s40620-021-01211-x

*Reason for exclusion: not original research (this is a poem)*

1. Simmons, C., Rodrigues, R., & Szebehely, M. (2022). Working conditions in the long‐term care sector: A comparative study of migrant and native workers in Austria and Sweden. *Health & Social Care in the Community*, *30*(5), e2191–e2202.

*Reason for exclusion: No main focus on infection risk*

1. Sorayyanezhad, Nikpeyma, N., Nazari, S., Sharifi, F., and Sarkhani, N. (2022). The relationship of caregiver strain with resilience and hardiness in family caregivers of older adults with chronic disease: a cross-sectional study. BMC Nursing, 21(1), 1–184. https://doi.org/10.1186/s12912-022-00966-3

*Reason for exclusion: No main focus on infection risk factors*

1. Timler, Timler, W., Bednarz, A., Zakonnik, Ł., Kozłowski, R., Timler, D., and Marczak, M. (2023). Identification and Preliminary Hierarchisation of Selected Risk Factors for Carbapenemase-Producing Enterobacteriaceae (CPE) Colonisation: A Prospective Study. International Journal of Environmental Research and Public Health, 20(3), 1960–. https://doi.org/10.3390/ijerph20031960

*Reason for exclusion: No main focus on home care*

*.*

1. Wyte-Lake, T., Manheim, C., Gillespie, S. M., Dobalian, A., & Haverhals, L. M. (2022). COVID-19 Vaccination in VA Home Based Primary Care: Experience of Interdisciplinary Team Members. *Journal of the American Medical Directors Association*, *23*(6), 917–922.

*Reason for exclusion: No main focus on risk factors in general; focus on vaccination only*

1. Wong, Grullon, J. R., and Lovier, M. A. (2022). COVID-19 risk factors and predictors for handwashing, masking, and social distancing among a national prospective cohort of US older adults. Public Health (London), 211, 164–170. https://doi.org/10.1016/j.puhe.2022.08.002

*Reason for exclusion: No main focus on home care*

1. Xu Y., Koh, X H., Chua, Y. T. S., Tan, C. G. I., Aloweni, F. A. B., Yap, B. E. J., Tan, P. C., Chua, X., Lim, Y. K. S., Oh, H. C., Teo, S. H. S., and Lim, S. F. (2022). The impact of community nursing program on healthcare utilization: A program evaluation. Geriatric Nursing (New York), 46, 69–79. <https://doi.org/10.1016/j.gerinurse.2022.04.024>

*Reason for exclusion: No main focus on infection risk factors*

1. Zhang, Y., Liu, X., Meng, Q., Li, B., & Caneparo, L. (2022). Physical environment research of the family ward for a healthy residential environment. *Frontiers in Public Health*, *10*, 1015718–

*Reason for exclusion: No main focus on infection risk factors.*
