# Supplementary material for: Risk factors for infection in older adults with home care: a mixed methods systematic review with meta-analysis
Source: BMC Public Health. 2025 May 3;25:1643. doi: 10.1186/s12889-025-22538-1 (PMC12048934; doi:10.1186/s12889-025-22538-1)
Supplement: Supplementary file 5 — Supplementary Material 5 [file 12889_2025_22538_MOESM5_ESM.docx]

**APPENDIX 5: List of studies excluded following critical assessment of methodological quality**

1. Russell D, Dowding D, Trifilio M, McDonald MV, Song J, Adams V, Ojo MI, Perry EK, Shang J. Individual, social, and environmental factors for infection risk among home healthcare patients: A multi-method study. Health Soc Care Community. 2021 May;29(3):780-788. doi: 10.1111/hsc.13321. Epub 2021 Feb 19. PMID: 33606903; PMCID: PMC8084932.

Reason for exclusion: Two ratings of *No* in the critical appraisal (qualitative study)

- Q6 - There is no declaration of researchers' cultural and theoretical orientation, nor information on researchers' critical examination of their own role and potential bias or how they responded to events during the study. There is only a basic statement on researchers' training and that they had no previous relation with the interview subjects.
- Q8 - There is no reference to what method of analysis was followed and why. Coding is well described (which includes application of inter-rater reliability), but not the development of themes. The presentation of themes in headings and subheadings appears out of place and is confusing. The authors present the findings by frequently mixing own words and participant words in the same sentence, making it hard to discern what is the voice of participants and what is researchers' analytic contribution. Little contradictory data is taken into account. No information is given on researchers' roles, bias or influence during analysis.

1. Bandini J, Rollison J, Feistel K, Whitaker L, Bialas A, Etchegaray J. Home Care Aide Safety Concerns and Job Challenges During the COVID-19 Pandemic. New solutions. 2021;31(1):20–9.

Reason for exclusion: Two ratings of *No* in the critical appraisal (qualitative study)

- Q6 - There is no declaration of researchers' cultural and theoretical orientation, nor information on researchers' critical examination of their own role and potential bias or how they responded to events during the study. There is no information regarding researchers' training, professional background or relation to agencies and interview subjects.
- Q8 - Coding process is described but the development of themes is not mentioned. Data is presented under sections, not themes, but no information is available on how they were developed. Satisfactory amount of data is presented to support findings. No information is given on researchers' critical examination of own role and potential bias during analysis.

1. Felemban O, John WS, Shaban RZ. Infection prevention and control in home nursing: case study of four organisations in Australia. British journal of community nursing. 2015;20(9):451–7.

Reason for exclusion: Five ratings of *No* in the critical appraisal (qualitative study)

- Q6 - There is no information on the researchers’ background, qualification or theoretical orientation. The article contains no information on critical reflection on researchers' role and potential bias.
- Q7 - Ethical approval was obtained but there is no information on informed consent or any other ethical considerations.
- Q8 - A reference is given to analysis method but no details are given regarding coding, development of themes and triangulation. Sufficient data is presented to support the findings, however it is unclear what data comes from interviews, focus groups and (if any) document review. No contradictory data is taken into account.
- Q9 - Findings are explicit but the discussion is inadequate. Only one reference is cited in the discussion and the findings are only used to develop recommendations. Some discussion of credibility and transferability is presented.
- Q10 - The researchers do not consider the findings in relation to current literature, nor do they propose new areas of research. They give a number of recommendations but it is difficult to assess the value of this research due to the methodological limitations.

1. White MC. Infections and infection risks in home care settings. *Infect Control Hosp Epidemiol*. 1992;13(9):535-539. doi:10.1086/646593

Reason for exclusion: Low trustworthiness with three *Unclear* and two *No* ratings (cross-sectional study)

- Q1 – There is little and concise information on setting and participants. No specific inclusion criteria are given, as for example age, diagnose, time of HC use, etc.
- Q2 – Study subjects are not described. We can find the characteristics in the results.
- Q3 – Exposure (risks factors) were retrieved from clinical charts but there is no information on who was filling the charts.
- Q5 – No confounders were identified.
- Q6 – No adjustment is mentioned. Throughout the text the author stratified by gender and by whether the clients had and infection or not, as well as comparing among "subgroups".

**FROM UPDATED SEARCH**

1. Rezende CP, Nascimento MMGD, França AP, Santos ASA, Oliveira IV, Oliveira DR. Caring for elderly people during the COVID-19 pandemic: the experience of family caregivers. Rev Gaucha Enferm. 2022 Jul 31;43:e20210038. English, Portuguese. doi: 10.1590/1983-1447.2022.20210038.en. PMID: 35920476.

Reason for exclusion: Four ratings of *No* in the critical appraisal (qualitative study)

- Q4 – The recruitment process is described but contains gaps. The authors claim to have used a theoretical sampling strategy but at the same time state that anyone who volunteered was interviewed. It is not clear how any selection was done to guide the sampling in the direction that they theoretically wanted.
- Q6 – There is no information on the researchers' background, qualification or theoretical orientation. The article only rudimentarily mentions that research is an interactive process but provides no critical reflection on the researchers' role and potential bias.
- Q8 – Although satisfactory amount of data is presented to support findings, no contradictory data is taken into account and the description of the analytical process lack sufficient information about the development of codes and themes. The theoretical framework that is referenced in the beginning is not connected to the analysis in any way. No information is provided on researchers' critical examination of own role and potential bias during analysis.
- Q9 – The findings are summarized, but the discussion primarily supports the findings with no critical reflection or arguments against. The discussion is mainly centered on recommendations, even though the aim of the study was to provide a model of understanding of experiences. No such model is presented. The limitations section is underdeveloped; no in-depth discussion on credibility and trustworthiness is provided.

1. Prout H, Lugg-Widger FV, Brookes-Howell L, Cannings-John R, Akbari A, John A, Thomas DR, Robling M. "I don't mean to be rude, but could you put a mask on while I'm here?" A qualitative study of risks experienced by domiciliary care workers in Wales during the COVID-19 pandemic. Health Soc Care Community. 2022 Nov;30(6):e6601-e6612. doi: 10.1111/hsc.14109. Epub 2022 Nov 24. PMID: 36426419; PMCID: PMC10100139.

Reason for exclusion: Two ratings of *No* in the critical appraisal (qualitative study)

- Q6 – There is no declaration of researchers' cultural and theoretical orientation. The researchers provide no critical reflection on the researchers' role and potential bias. It seems that all data collection and analysis was done by one person, with no discussion or reflection together with any of the co-authors.
- Q8 – Inadequate description of the analysis process is provided. Only three sentences describe the analysis, it is therefore hard to know how coding was conducted, how themes were developed, and who, if anyone besides the main author was involved in this process. Sufficient data is presented to support findings. No contradictory data is considered.

1. Chikanya VK, James S, Jardien-Baboo S. Home-based care of stroke patients in rural Zimbabwe: Knowledge of caregivers. J Stroke Cerebrovasc Dis. 2023 Jan;32(1):106830. doi: 10.1016/j.jstrokecerebrovasdis.2022.106830. Epub 2022 Nov 9. PMID: 36370506.

Reason for exclusion: Low trustworthiness with three *Unclear* and three *No* ratings (cross-sectional study)

- Q1 – Inclusion criteria are not clearly described (contradictory statements make the inclusion criteria hard to understand)
- Q3 – Self-report and interview-guided questionnaires were used, but no information about the questions are provided.
- Q4 – No information is provided on this; the paper only states that participants assisted in identifying patients.
- Q5 – No confounders are mentioned
- Q6 – No confounders are mentioned
- Q7 – Some detail about validation of translating the questionnaires are given, but no other information about measurement reliability is presented
