# Supplementary material for: Risk factors for infection in older adults with home care: a mixed methods systematic review with meta-analysis
Source: BMC Public Health. 2025 May 3;25:1643. doi: 10.1186/s12889-025-22538-1 (PMC12048934; doi:10.1186/s12889-025-22538-1)
Supplement: Supplementary file 6 — Supplementary Material 6 [file 12889_2025_22538_MOESM6_ESM.docx]

**Appendix 6. Characteristics of included qualitative studies**

| **Study** | **Aim** | **Geographical setting** | **Methods** | **Participants** | **Context** | **Phenomena of interest** |
| --- | --- | --- | --- | --- | --- | --- |
| Baumbusch *et al.* 2022 | Explore the impacts of pandemic public health measures and ways that families adopted and adapted to those measures, loss of supports and the subsequent consequences for their mental and physical wellbeing. | British Columbia, Canada | Semi-structured interviews (28) and diary entries (34); thematic analysis | 12 family caregivers of people living with dementia | Home based care by family members to people living with dementia | Perceptions and understanding of infection risk and experiences of barriers to infection prevention and control practices in home care |
| Bell *et al.* 2022 | To describe the experiences of home-based care providers, specifically among Medicare-certified home health agencies, in providing care to older adults during a novel pandemic in order to inform future disaster planning. | United States - New York, Illinois and Michigan | Qualitative descriptive; Telephone interviews; Abductive analytic approach. | 27 staff | Medicare-certified home health agencies from eight counties. | Infection prevention and control practices during a pandemic. |
| Dowding *et al*. 2020 | To understand (1) if and how home care nurses identify patients at high risk of infection and (2) the strategies they use to mitigate that risk. | United States - New York | Qualitative descriptive; Semi-structured interviews (n=50); Thematic analysis. | 50 Home health care nurses | Not for profit, Medicare certified home health care agency. | Perception of infection risk; Risk mitigation strategies. |
| Emmesjö *et al.* 2022 | To describe the experiences of nurses and physicians working in home health care in an integrated care model during the COVID-19 pandemic. | Sweden | Semi-structured interviews (n=14); thematic analysis | 6 registered nurses and 8 physicians | Mobile Integrated Care  Model in a region (administrative county that has responsibility for health care) | Infection prevention and control practices during a pandemic. |
| Franzosa *et al.* 2022 | To describe aides’ roles in supporting veterans and working with primary care teams during COVID-19 and identify COVID-related changes in tasks | USA | Semi-structured interviews (n=23); Thematic analysis using a combined  inductive and deductive approach. | 23 (8 home aides, 6  agency administrators, and 9 primary care team  members) | Veterans Affairs Medical Center  is a tertiary care center that provides clinic and home-based primary care  services to veterans in the Bronx, New York | Perceptions and understanding of infection risk and experiences of barriers to infection prevention and control practices in home care |
| Moi *et al.* 2022 | To explore the experiences of Norwegian home-care nurses in the frst wave of the Covid-19 pandemic | Norway | Semi-structured in-depth interviews (n=12); thematic analysis | 12 registered nurses | Community-based health care in a municipality in South Norway | Infection prevention and control practices during a pandemic. |
| Osakwe *et al.* 2021 | To understand the infection prevention and control needs and challenges associated with caring for patients during the pandemic. | United States - New York | In-depth semi-structured interviews; Conventional content analysis. | 25 staff | Four licensed home care service agencies. | Infection prevention and control during a pandemic; Experiences of Spanish-speaking staff with limited English proficiency during the pandemic. |
| Pogorzelska-Maziarz *et al.* 2020 | To (1) explore a range of HHC staff perspectives on agency-level infection prevention and control priorities and implementation of policies, and (2) describe challenges and successes associated with infection prevention and control in HHC agencies. | United States | Qualitative descriptive; Semi-structured telephone interviews; Directed content analysis. | 41 staff | 13 home health care agencies from various regions. | Infection prevention and control practices. |
| Sterling *et al.* 2020 | To understand the experiences of home health care workers during the COVID-19 pandemic and to elucidate the challenges regarding disease transmission, preparedness, and well-being, to inform future studies, interventions, and policies. | United States - New York | Video-conference individual interviews; Grounded theory analysis. | 33 staff | 24 home care agencies. | Infection prevention and control practices during a pandemic. |
| Tavemark *et al.* 2022 | To describe health care professionals’ experiences of their work environment in home care and home health care during the COVID-19 pandemic. | Sweden | Semi-structured telephone interviews (n=27); content analysis | 27 health care professionals (assistant nurses, nurses, occupational therapists, physical therapists) | Municipal care in two municipalites in Sweden | Infection prevention and control practices during a pandemic. |
| Wendt *et al.* 2022 | To explore infection prevention practices and related behavioural factors in nurses and clients to identify barriers and facilitators of infection prevention practices in home-based nursing care. | Netherlands | Qualitative exploratory; Observations (n=87), Focus groups (n=3), Semi-structured interviews (n=11); Inductive data-driven analysis and deductive theory-driven analysis. | 20 nurses and professional caregivers, 80 clients | Four health care organisations providing home-based care. | Infection prevention and control practices. |
