# Supplementary material for: Risk factors for infection in older adults with home care: a mixed methods systematic review with meta-analysis
Source: BMC Public Health. 2025 May 3;25:1643. doi: 10.1186/s12889-025-22538-1 (PMC12048934; doi:10.1186/s12889-025-22538-1)
Supplement: Supplementary file 7 — Supplementary Material 7 [file 12889_2025_22538_MOESM7_ESM.docx]

**Appendix 7. Characteristics of included quantitative cohort studies**

| **Study** | **Aim** | **Geographical setting** | **Study design, methods, setting** | **Participants** | **Outcome of interest, outcome measure** |
| --- | --- | --- | --- | --- | --- |
| Noguchi et al., 2020 | Estimate the incidence and prevalence of infectious diseases and their risk factors among patients who use visiting nursing services in Japan. | City A. in Kanagawa prefecture, Japan | Prospective cohort study design. Surveyed patients who use visiting nursing services and conducted a follow-up for one year. Self-administered questionnaire surveys were performed at the baseline and one year later. | 506 agreed, 419 patients in the final analysis | All infectious disease  Self-report data to calculate rates. |
| Osakwe et al., 2019 | To assess the risk factors  for UTI-related hospitalization among elderly HHC patients and to examine the relationship between UTI-related hospitalization and ADLs. | All USA | Retrospective cohort design. Secondary data analysis of a 5% random sample of a national HHC dataset, the Outcome and Assessment Information Set for the year 2013. | 24,887 hospitalized HHC patients of which 1,133 had UTI-related hospitalizations | UTI hospitalizations and associated factors, especially the relationship between UTI-related hospitalization and ADLs using odds ratios. |
| Shih et al., 2019 | To investigate the incidence and factors related to UTIs both in outpatient and inpatient settings, and to propose strategies to prevent UTI and improve the quality of home care. | Taiwan, China | Retrospective Cohort Study. Data were retrieved from the home care service system database. | 598 patients | UTI episodes and associated factors.  Odds ratios of UTI among patients receiving HHC, compared to those without UTI. |
| Yokobayashi et al., 2013 | Elucidate the incidence of fever events reported in the medical records in elderly people under home medical management, diagnosis at the time of fever onset and outcome | Tokyo, Japan | Retrospective cohort study. Data were retrieved by reviewing medical records, in the Seikyo Ukima Clinic in Kita Ward, Tokyo, Japan. | 105 patients | Fever events and the disease causing the fever events.  Risk ratios, hazard ratios and incidence rates were calculated |
| Yokobayashi et al., 2014 | Determine the incidence of fever among elderly persons under home medical management, diagnosis at the time of fever onset and termination, and to assess whether level of care-need, activities of daily living (ADL) and cognitive function can predict the onset of fever | Tokyo, Japan | Prospective cohort study. Data were obtained from nurses and/or trained home helpers that recorded temperature, or from family members who measured temperatures when they felt that the patient seemed ill.  Five clinics that serve the 23 wards of Tokyo, Japan were involved in the study. | 419 participants | Incidence and hazard ratios of fever events and causes of fever events among those getting fever compared to those not developing fever. |
| White et al., 1995 | Describe urinary catheter-related infections that occurred among home care patients. | California, USA | Historical prospective cohort study. Data were collected via chart review. | 106 patients (81 patients included in the final analysis) | Hazard ratios of urinary catheter-related infections, characteristics of patients who do, as compared to those who do not, get UTIs;  influence of the interval between catheter changes on the length of time patients remain free of UTIs in the home setting. |
