# Supplementary material for: Risk factors for infection in older adults with home care: a mixed methods systematic review with meta-analysis
Source: BMC Public Health. 2025 May 3;25:1643. doi: 10.1186/s12889-025-22538-1 (PMC12048934; doi:10.1186/s12889-025-22538-1)
Supplement: Supplementary file 8 — Supplementary Material 8 [file 12889_2025_22538_MOESM8_ESM.docx]

**Appendix 8. Characteristics of included cross-sectional studies**

| **Study** | **Aim** | **Geographical setting** | **Study design, methods, setting** | **Participants** | **Outcome of interest, outcome measure** |
| --- | --- | --- | --- | --- | --- |
| Barros et al., 2018 | Estimate the frequency of the first occurrence of acute lower respiratory infection and death and its associated factors in patients who attended the Home Care Public Program of the Regional Health Authority in Sobradinho in the DF, Brazil | Sobradinho, Brazil | Analytical cross-sectional study. Data were obtained from the patients’ medical records. The initial sample included 150,867 HHC patients from 8,255 HHC agencies nationwide. | 973 patients | Odds ratios of Infection events defined as a hospitalization and/or ED visit caused by any of the following three types of infections: respiratory, wound site, or urinary tract infection (UTI) |
| Harrison et al., 2022 | Evaluate the association between HHC agencies’ policies related to urinary catheters and hospital transfers due to UTI in patients with urinary catheters. | USA | Cross-sectional. Data were linked using a nationally representative HHC agency-level survey and patient data from the Outcome and Assessment Information Set and Medicare inpatient data. | 28,205 Medicare beneficiaries | Probability of hospital transfers due to UTI among patients with urinary catheters during a 60-day HHC episode. |
| Lin et al., 2020 | Evaluate the risk factors associated with hospitalization for CAP in HHC patients. | Taiwan | Retrospective cross-sectional study. Data obtained from reviewed medical records (including home visit records, outpatient department records, admission notes, discharge summaries, and laboratory data).  The study was conducted in the home health care unit of a medical center in northern Taiwan. | 598 home health care patients | Community-acquired pneumonia (CAP) and related risk factors; odds ratios were calculated. |
| Marrie et al., 2005 | Describe the epidemiology, clinical features, and outcomes of community acquired pneumonia (CAP) requiring hospitalization in patients receiving home care | Edmonton, Canada | Prospective study in six hospitals in Canada. | 540 of 2464 patients with home care and CAP | Community-acquired pneumonia (CAP) requiring hospitalization in subjects receiving,  Clinical symptoms and radiological evidence |
| Morioka et al., 2021 | Describe the nationwide situation of IPC practices among home-care nursing agencies and compare those by agency size, and to explore whether such practices are associated with the occurrence of infection among patients who use home-care nursing services. | Japan | Secondary analysis using data from a cross-sectional survey investigating patient safety and IPC practices among nationwide home-care nursing agencies. | Only data on agencies: 370 agencies were included | Infections detected in the last 3 months, (respiratory, skin, soft tissues, urology and catheter-related infections), odds ratios were calculated. |
| Pärn et al., 2016 | Estimate the UTI prevalence and antimicrobial use among the general HC population across Finland and to identify the factors associated with antimicrobial use and UTI. | Finland | Cross-sectional study. Use of Resident Assessment Instrument (RAI) data.  Community setting including 15 out of 320 municipalities in Finland; corresponding to 12% of all the HC clients in Finland. | 6887 home-care clients | Odds ratios of variables significantly associated with UTI episodes and antimicrobial agents. |
| Rönneikkö et al., 2018 | Identify conditions that could be targeted in the care planning of home care clients to prevent hospital admissions. This study extends an earlier analysis (Ronneikko et al., 2017), and describes the most common discharge diagnoses. Also analyzes how patient characteristics are associated with reasons for hospitalization. | Finland | Nationwide register data and Resident Assessment Instrument for Home Care (RAI−HC) assessments | 6764 patients | Infection-related reasons for hospitalization. Odds ratios were calculated. |
| Shang et al., 2020 (1) | Identify risk factors for three types of infection in HHC patients using the Outcome and Assessment Information Set (OASIS), and to explore the potential role of informal caregivers in prevention of infections in the HHC setting. | USA | Secondary data analysis of a 5% random sample of the 2013 national OASIS data. The initial sample included 150,867 HHC patients from 8,255 Medicare certified HHC agencies nationwide. | 128,163 patients | Infection events defined as a hospitalization and/or ED visit caused by any of the following three types of infections: respiratory, wound site, or urinary tract infection (UTI).  Odds ratios were calculated. |
| Shang et al., 2020 (2) (Risk model) | Develop and test predictive risk modelling to identify patients’ risk of infection-related hospitalization or emergency department use, and to identify important risk factors associated with infection-related outcomes. | USA | Medical records review, including home visit records, outpatient department records, admission notes, discharge summaries, and laboratory data.  Records of all patients served by the agency’s adult HHC program who were admitted during the study period were reviewed. | 1,908 patients (1.69% of total sample). | Respiratory, wound, urinary tract, and intravenous catheter-related. Odds ratios were calculated. |
| Shang et al., 2022 | HHC influenza vaccination rates and policies, and the association between HHC agency vaccination policies and HHC patient hospitalizations due to respiratory infection, stratified by the agency ownership. | USA | Data were collected with a national survey of HHC agencies on infection prevention and control (IPC), linked with multiple national HHC agency- and patient-level datasets.  The 460 included HHC agencies (nationally weighted sample) were primarily for-profit (69.1%) and located in urban areas (86.5%). | 156,408 patients | Influenza vaccination rates among HHC workers.  Agency policies related to staff influenza vaccination and their impact on HHC patient outcomes (hospital transfers due to respiratory infection).  Agency ownership effects on these relationships.  Calculations of predictions of avoided infections (comparing patients in agencies with more vaccinated staff to those with fewer vaccinated statt). |
