# Supplementary material for: Risk factors for infection in older adults with home care: a mixed methods systematic review with meta-analysis
Source: BMC Public Health. 2025 May 3;25:1643. doi: 10.1186/s12889-025-22538-1 (PMC12048934; doi:10.1186/s12889-025-22538-1)
Supplement: Supplementary file 9 — Supplementary Material 9 [file 12889_2025_22538_MOESM9_ESM.docx]

**Appendix 9. Study findings with illustrations**

| **Study: Dowding et al 2020** | |
| --- | --- |
| Finding 1 | Nurses identified several factors that they felt put patients at a higher risk of infection. Key indicators included having a history of infections or a prescription for an antibiotic. (U) |
| Illustration | “I always check to see if the patient has a communicable disease if they're on any contact precaution. I check to see if there's any active infection, if the patient's on antibiotics, if there's anything that we have to be aware of.” (Supplement table 3 Q5) |
| Finding 2 | Risk of infection was the result of a combination of factors that affected a patient’s immune system such as older age, diabetes, dehydration or having inadequate nutrition. (U) |
| Illustration | “Well, elderly patients get dehydrated, and they get infection quite easily. Some of them are diabetics, which makes them more prone for urinary tract infection.”“If someone’s immunocompromised, they’re going to be higher risk of infection if they don’t have great nutrition.”(Supplement table 3 Q6&Q7) |
| Finding 3 | Having particular points of entry for infection, such as intravenous (IV) lines, Foley catheters and wounds were identified as factors that automatically put a patient at a higher risk of infection. (U) |
| Illustration | “I think depending on if they have any open devices, like Foley catheters or any open devices that will put them at risk. That will automatically put them at risk. If they have an IV, a Foley, or any- thing that has an open orifice within the body, then that will first put them at risk.” (p.5) |
| Finding 4 | The risks for urinary tract infection were complex with factors such as cognition, incontinence, availability of caregivers and environmental conditions all having a role to play. (U) |
| Illustration | “So a lot of times elderly people that are incontinent, they tend to get urinary tract infections pretty easily. So probably an older person who's incontinent, who's in bed, who maybe doesn't have a lot of help.”. “If you’re any degree of dementia or Alzheimer’s, and there’s incontinence involved, and there’s not an adequate caregiver or not enough of a caregiver, they develop urinary infections.” (Supplement table 3 Q8 & Q9) |
| Finding 5 | Wounds were a key infection risk and their assessment and management are a large part of the nurses’ work. (U) |
| Illustration | “Redness, pain, swelling. A warm area around the wound, and if it started draining foul-smelling drainage. And if pus coming from the wound, we definitely have a problem. That’s definitely have bacterial thing going on there.” (Supplement table 3 Q10) |
| Finding 6 | Patients who also had a diagnosis of diabetes were at particular risk and nurses discussed in detail their process of wound assessment to ensure they detected any infections quickly. (U) |
| Illustration | “People with diabetes who have wounds, and their sugars are not well controlled, which doesn't always necessarily mean that it's their fault, but number one, those high blood sugars could indicate infection because blood sugars go up when you have infection. So that's one thing you think of. The other thing is they're just eating badly and not taking care of their glucose control which can really affect wound healing badly.” (Supplement table 3 Q11) |
| Finding 7 | A diagnosis of chronic obstructive pulmonary disease (COPD), and a history of smoking, were specific factors related to a risk of respiratory infections such as pneumonia. (U) |
| Illustration | “Because somebody with COPD is obviously at higher risk of developing a lung infection, right? But not necessarily wound infection. So it depends what type of infection you're talking about. Certain people are more at risk for developing respiratory infection because of smoking and I think smoking always puts you at higher risk for infection, in general.” (Supplement table 3 Q12) |
| Finding 8 | Nurses regarded patients’ knowledge and understanding of their illness and factors associated with infection risk and infection prevention behaviours as being closely linked to their risk of infection. This included issues such as culture and beliefs, personal hygiene practices and their understanding of how infections were spread and could be prevented. Overall nurses felt their patients lacked basic knowledge about infection prevention, including basic health and hand hygiene, or held beliefs and attitudes towards infection control that nurses perceived were not at a “high standard. (U) |
| Illustration | “Lack of knowledge. Lack of education about infection control for one. Poor health hygiene. Sometimes even culture. Some people have certain beliefs about certain things, so they don’t think infection control is–they don’t put it as a high standard” (p.5) |
| Finding 9 | Patients’ behaviours, in relation to general health promotion such as getting vaccinations, impacted on their infection risk. (U) |
| Illustration | “Lots of them don't get vaccinated at all. A lot of them don't get yearly flu shots. They don't get the pneumonia vaccine which is recommended every five years. So a lot of them are at risk for pneumonia.”(Supplement table 3 Q13) |
| Finding 10 | Patients’ behaviours, in relation to personal hygiene and cleanliness impacted on their infection risk. |
| Illustration | “Some patients, like I said, they do practice good hygiene, meaning they'll take a shower every day, wash up every day, etc., and there are others that their normal day of life means not taking a shower for two weeks, or they don't even have-- they don't think that they're dirty when in fact they are dirty. Or you go in, and the dressings are super soiled, either with feces or urine if it's a wound that's on the back of the thigh, and they think that they're clean, and they're actually not.” (Supplement table 3 Q14) |
| Finding 11 | Adhering to treatment recommendations, such as following medication regimens (e.g. courses of antibiotics), managing their wounds, diabetes management and hand washing were important considerations for assessing infection risk. (U) |
| Illustration | “Not hand-washing. Not properly using universal precautions. Not taking their medications as prescribed.”(p.5) |
| Finding 12 | If patients lived in an environment that was unclean, then this increased the risk of a patient getting an infection. (U) |
| Illustration | “The cleanliness of their environment, if their homes are dirty, that’s a big factor in the contribution to infection. ”(p.5) |
| Finding 13 | Nurses reported patients living in apartments without running water, or a working sink. (U) |
| Illustration | I1.13. “Number one, apart from asking, physical assessment, looking at their environment. Is this patient living in a filthy environment? Is there a working bathroom sink, a working kitchen sink? To me, those are primary factors. If there’s no working sink, how do they wash hands? Where do they go to use the bathroom if they need to? If the tap is full of junk. Some people pack goodies in their tubs, and you ask them, “Do you shower? Do you use tubs?” And they tell you, “I just wipe down.” (Supplement table 3 Q15) |
| Finding 14 | Nurses reported patient environments with clutter, trash and infestations of cockroaches and rodents. |
| Illustration | “It’s just lack of good health standards and lack of sanitary facilities. Homes that are roach infested. Homes that are cluttered, just dirty. We have a lot of hoarders, unfortunately.” (Supplement table 3 Q16) |
| Finding 15 | When patients lived with many family members in close proximity and their infection control practices were not thought to be of a high standard, this was also seen as a risk factor. (U) |
| Illustration | “I think number one is the environment. Some of them are not clean. They are so close together. Everybody is everywhere. You know what I’m saying? It’s too many people, and their sanitary practice in their house is not the greatest. And they share stuff. They don’t wash hands.” (Supplement table 3 Q17) |
| Finding 16 | Environments where pets were present also put patients at higher risk of infection. Nurses viewed the mere presence of a pet as introducing an additional source of risk. However, they also highlighted how, for some patients, their general hygiene practices and how they cared for their pets increased their risk. |
| Illustration | “I would say also if they have pets and they’re not really keeping, maybe, everything clean. And they’re just exposing themselves to all different types of microbes and bacteria that could be around if they don’t really clean. That could put them at risk for an infection.” (Supplement table 3 Q18) |
| Finding 17 | Patients who had caregivers or family that were not following advice or being involved in care were seen to be at an increased risk of developing infection. (U) |
| Illustration | “Well, things like sometimes they’ll have a family member doing the wound care or they might be doing it. They’re not wearing gloves. A lot of times, family members don’t wear gloves.”(p.6) |
| Finding 18 | Overall, there has to be an underlying reason why an individual is at risk of infection. (U) |
| Illustration | “there is a risk for every person and every patient. But there also has to be certain comorbidities to go along with risk of infection. It’s not that you walk into every home and you kind of suspect, “Oh, this person is at risk for–” there has to be some sort of underlying condition.”(p.6) |
| Finding 19 | In the context of homecare practice in the USA, nurses must adjust their level of care to what is covered by the patient’s insurance, which may require some patients to monitor their own clinical status more than they would in other clinical settings. (C) |
| Illustration | “Yes, a lot of the patients, their insurance won't cover certain supplies, so we really have to try to do the most that we can with the little we have, or sometimes we have to bring supplies to the patients, especially saline or gauze pads and tape, etc.”(Supplement table 4 Q20) |
| Finding 20 | Nurses faced particular challenges when talking to a patient about cleaning up their home to avoid infection, and often patients did not have a suitable family caregiver to support the nurse’s attempts at reducing infection risk. (U) |
| Illustration | “Some of them are receptive to it. Or when you come in, they’re already saying, ‘Oh, pardon us. But we’re trying to clean up,’ but every time you come, it’s just the same thing. But I try just to do it like, ‘Oh, for your own safety and health, it’s better to keep this clean. Make sure all your supplies are in a clean container.’ But some of them will respond, ‘Oh, I’m doing okay. I’ve been like this a long time, all my life.’ Yeah. Yes, and some instance, rarely-- I have had instance we have to call the Adult Protective Services with the condition of the home that’s really unsafe when it comes to cleanliness and safety.” (Supplement table 4 Q28) |
| Finding 21 | Both patients and caregivers may show a resistance to change, may have low health literacy, and may be cognitively impaired. (C) |
| Illustration | “Then you’re looking at their attitude towards what you’re talking about when you ask them to wash their hands. Because I’ve had patients who’ve said to me, ‘You’re washing your hands again?’ So yeah, the attitude towards you. Sometimes they think that one time is enough, and that’s it. And then how receptive they are to you just teaching them. Sometimes they’re listening but not listening. Sometimes they’ll say, ‘Uhhuh,’ but you know that it’s just going in one ear, then going out the other.” (Supplement table 4 Q29) |
| Finding 22 | Mental health –especially stress –also presented a challenge to patient and caregiver education. (C) |
| Illustration | “If they’re depressed, the simple depression scale is important as well because I also feel that all of our [possible limitations?] are set forward in our minds. So if someone’s depressed, there’s nothing I can do to help them. There’s nothing I can teach them the importance of preventing infection with this open wound or the importance of toileting this old lady every two hours. Drinking fluid, toileting every two hours to kind of have a bladder regimen to prevent the UTI, because she’s so depressed, it’s not important. “I’ll just sit here in this diaper.”(Supplement table 4 Q30) |
| Finding 23 | The nurses viewed their computer/tablet as a potential vector for infection, particularly as patients were meant to use their finger or a stylus provided by the nurse to sign the tablet as a record of their care provided during the home visit. (U) |
| Illustration | “Yeah, [I want] Clorox wipes to wipe down our computer because a lot of these patients, they don’t wash their hands, and then they're touching our computer. And a lot of them are in their bed. They can’t even get to the kitchen, much less the bathroom, and [management is] demanding us to sign the note. And sometimes that pen, I think if they put that pen on a Petri dish…”(Supplement table 4 Q35) |
| **Study: Wendt et al 2022** | |
| Finding 24 | The nursing care environment varied from impeccably clean and tidy –without visible dirt, dust or damaged interiors –to dilapidated and contaminated house- holds. The latter were littered with putrid waste, sticky floors, damaged interiors and inadequate lighting and lacked fresh air or adequate space for movement. (U) |
| Illustration | […] the house in general is: old, messy, dirty and full. It smells of stale urine and wet dog. […] The bedroom is an old hobby room of approximately 20 m 2 . In the room there is a single bed (not adjustable in height), five chairs, a table, a filing cabinet, a freezer, a linen closet, a display case, a bedside table and a gas stove. All five chairs are in use because they are hung with clothes, towels and bedding or because there is a tray with a saucer and a small cow bell on it. The walls are covered with paintings and picture frames, a wall lamp without hood and a red plastic jug. Uncovered areas are stained brown-yellow, [and] in several places the wallpaper curls, and in the corners of the room, cobwebs can be seen in the light. […] All horizontal surfaces are covered with a towel or carpet, on which all kinds of things are placed: glass vases, works of art, religious statues, candles, porcelain ashtrays (empty), a card game, cans with unknown contents, bottles of soap and shampoo, card- board boxes with magazines, bookends, a sewing machine, a teapot, ski poles and continence material for women. Stains can be discovered on several of the towels and carpets. The windows in the bedroom are closed, and there are no ventilation grills in the room. [Observation] (p.4) |
| Finding 25 | Besides the client and nurse, other people, […] could be present during the delivery of care, varying from none to many; for example, healthcare staff, relatives, acquaintances, children, domestic help […] (U) |
| Illustration | "During the delivery of care, more and more visitors arrive. First one adult woman. Then one adult female and adult male with toddlers (approx. 14 months). Then another adult woman with a baby. Toddlers run around the room, sneeze three times successively on chair, wheelchair and side table without covering nose or mouth. The baby crawls through the room. The doorbell rings regularly, coffee is made (beans are ground and the machine is buzzing), there is noise from cups and saucers being prepared, visitors are talking simultaneously. The toddlers scream with joy. The baby cries. Client's partner's phone rings with a loud song. The visitors talk louder and louder to be able to make themselves understood. Wheelchair tires creak over the slippery floor. The IV-pump is beeping." [Observation] (Supplementary table 1 Q3) |
| Finding 26 | Besides the client and nurse, […] animals […] could be present during the delivery of care, varying from none to many; for example, […] pets, farm animals and pests. (U) |
| Illustration | "But the rats, the chickens, everything was running around there. Everything was running on the floor [...]" [Focus group] (Supplementary table 1 Q3) |
| Finding 27 | The workspace was cleaned irregularly before use. When the surface was cleaned with an alcohol- based disinfectant, it was regularly visibly wet when materials were placed on it. Sometimes communication devices or workwear touched the clean workspace. (U) |
| Illustration | "The table is not cleaned or disinfected before use." [Observation] "The table is still visibly wet when the caregiver starts preparing the materials[...]" [Observation] "During the delivery of care, the nurse stands with the vest and scarf against […] the 'clean working area' on which items are prepared". [Observation] (Supplementary table 1 Q6, Q7 & Q8) |
| Finding 28 | In some cases, there was not enough of a free flat surface to create a clean workspace. (U) |
| Illustration | During the delivery of care, there is no “clean working area”to be identified. Except for a small corner on which items are prepared, the table is packed with stacks of magazines, a cardboard box with a stockpile of materials, two large format fake leather wallets and a keyring. [Observation] (p.5) |
| Finding 29 | Cluttered and contaminated households presented nurses with a dilemma. Nurses indicated that they could not refuse to provide care because clients have a ‘right to receive healthcare’, but at the same time there is a need for guidance on how to address contaminated households to minimise the spread of infectious microorganisms. (U) |
| Illustration | You have a duty of care, […] but to what extent[…]? [Focus group] It’s a very large grey area right now, and I think as caregivers we’re going pretty far. We don’t scare easily. […] But where are the limits? [Focus group] (p.5) |
| Finding 30 | Considerable differences were found in clothing worn while providing care, from casual, day-to-day clothing to uniforms. […] Additionally, it was often up to nursing staff to clean their own workwear, but casual clothing is not cleaned at the recommended temperature, as nurses find this temperature would damage their clothing. (U) |
| Illustration | The nurse wears a long, loose-fitting vest down to the knees with a scarf draped loosely over the chest and stomach to below the waist. [Observation] "The nurse wears a white uniform jacket with a blue-red collar and an embroidered logo of [healthcare provider] on the left side of the chest. Underneath, she wears a thick dark checkered cotton blouse with rolled up and folded sleeves up to just below the elbows." [Observation] Interviewer: “And do you wash those [uniforms] yourselves […]?" Respondent: “Yes.”[Focus group] "I can wash all kinds of things, but not at sixty [degrees]." [Focus group] (Supplementary table 1 Q11, Q12 & Q13) |
| Finding 31 | Nurses pay little or no attention to the bags they carry to and from different households. (U) |
| Illustration | And whatever you just brought up, huh? How dirty is your bag? I have never thought about that. I just put my food in it. [Focus group] (p.5) |
| Finding 32 | F2.8. Materials, tools and equipment needed for home-based nursing care were stored in various ways, sorted in plastic or cardboard boxes with or without a closable lid or loose in plastic or paper bags. These boxes or bags were kept on tables, chairs, the floor or under a bed. In some cases, a cupboard, dresser or desk was used to store materials using different drawers for different materials. In a few cases, there was no obvious place to store materials. (U) |
| Illustration | I2.8."Supplies are in a transparent plastic container with a lid." [Observation] "On a chair at the dining table is an open cardboard box containing the supply of materials." [Observation] "All wound care materials are in a paper bag branded by a service pharmacy." [Observation] "The plastic crate is placed on the floor upon entry, then on the table, then back on the floor." [Observation] "All (utilitarian) materials are kept in a closed dresser in the bedroom." [Observation] "Left of the bed six large cardboard boxes and right of the bed eight large cardboard boxes." [Observation] (Supplementary table 1 Q15) |
| Finding 33 | Both the use of safety needles and regular needles were observed. (U) |
| Illustration | "During the I.M. injection, the nurse uses a ‘safety needle’." [Observation] 'Regular needles' are used." [Observation] (Supplementary table 1 Q17) |
| Finding 34 | Gloves used to put on compression stockings were regularly not cleaned before or after use, and when they were cleaned, in most cases hand disinfectants were inappropriately used. In some instances, these gloves stay behind in the house of the client to be used by different nurses [T2Q21], but on other occasions nurses take the gloves with them and use the same gloves to help multiple clients [T2Q22]. (U) |
| Illustration | Next to the basket are green rubber studded [brand name] gloves. The nurse herself also carries the same green rubber gloves: the gloves are folded together, and the name of the nurse is written on the inside with a ballpoint pen. No hand hygiene is applied before or after use of the gloves, nor are the gloves themselves cleaned. [Observation](p.5) "The nurse used yellow household gloves from the client's kitchen cabinet to put on the compression stockings." [Observation] "The gloves were lying on a table next to the bed in the client's bedroom. After use, the gloves are put back on the table." [Observation] "I come from an organization where it was normal for people [clients] to buy their own gloves." [Focus group] "The policy is that we wear personal gloves [...], for one employee only." [Focus group] (Supplementary table 1 Q20, Q21 & Q22) |
| Finding 35 | In some cases, sharps containers were full or filled above the ‘do not exceed’ line or handled inappropriately. (U) |
| Illustration | Sticking out of the needle container are plastic blister bags that had medication in them. Before the needles can be thrown away, they must first be removed. The client says: "I’ll just empty it". The nurse asks: "How will you do that?" The client responds: "I’ll just chuck it in the bin”. [Observation] (p.5) |
| Finding 36 | Some used materials and waste were also found scattered across various surfaces [T1Q25] for the client or someone else to throw away [T1Q26]. Additionally, food waste was observed in a few instances [T1Q27]. On occasion, contaminated waste from a nursing procedure came into contact with other persons such as toddlers and spouses. (U) |
| Illustration | "Packaging and used materials and caps and broken 'tabs' are on the 'clean working area', on the kitchen table, on the bedside table and on the corner of a sideboard at the end of the care moment." [Observation] "Waste stays on the kitchen table. The client's housemate separates the waste into 'plastic waste' and 'residual waste'." [Observation] "There's a black plastic bowl with a transparent lid containing a remnant of a salad. There's a slice of ham in it that doesn't look fresh anymore." [Observation] "[…] a child's toy lies on the table between the nurses’ waste. The nurse asks if this toy should also be thrown away. The toy is then taken from the rubbish and returned to the baby". [Observation] "After removing the PICC line, the client's partner picks up the PICC line and throws it away touching the side that was inside the body." [Observation] (Supplementary table 1 Q25, Q26, Q27, Q28) the toddler sees an opportunity to take the used ’non- return valve’ from the table and put it in her mouth. The mother responds to this and says that the toddler has to spit out what she took and she did. [Observation] (p.5) |
| Finding 37 | Two forms of hand hygiene were observed, one using an alcohol-based disinfectant and another washing at a washing stand or kitchen sink (with or without soap). Nurses, professional care- givers and clients then dried their hands in various ways, such as using paper tissues, cotton towels or the sides of their uniforms. [...] The observations revealed that hand hygiene was varying, inconsistent and irregular. (U) |
| Illustration | I2.13. Hands are dried with paper tissues from a box on the sink." [Observation] "The hands are then dried with the dark towel on a hook to the left of the sink."[Observation] "The nurse dries the hands on the side jacket pockets of the uniform." [Observation] (Supplementary table 2 Q1). Neither before nor after the using the rubber gloves [was] hand hygiene […] performed. [Observation] Before flushing the PICC-line, no hand hygiene was observed. [Observation] (p.6) "No hand hygiene observed in between different activities." [Observation] “Neither before nor after the administration of the eyedrops hand hygiene was performed.” [Observation] “Upon entering the client’s room, the nurse washes her hands with water and liquid soap from a bottle with a dosing pump. The duration of the handwashing is short (less than 10 seconds) and no foam was visible on the hands” [Observation] (Supplementary table 2 Q5) |
| Finding 38 | Nurses indicated that they had reservations regarding whether the World Health Organisation’s recommended ‘Five Moments for Hand Hygiene’ ‘fit’ the home-based nursing care environment. (U) |
| Illustration | Is it really necessary for us to do this hand hygiene –even within the client’s house –or is it unnecessary in the home environment? Because that is all based on research done in the hospital. Look, there, I get it. [Focus group] (p.5) |
| Finding 39 | On occasion nurses lacked the right materials for in-home care, such as disinfectants, gloves and aprons. (U) |
| Illustration | No gloves [were] observed in the house . [Observation] We’re out of aprons, we just […] We just walk like this [without aprons]. [Focus group] (p.6) "Upon entry, there is no hand alcohol in the house. The nurse washes her hands at the kitchen sink; [...] There is no liquid or other soap on the kitchen sink". [Observation] "The nurse doesn't wear overshoes or plastic aprons while showering [the client]." [Observation] (Supplementary table 2 Q6) |
| Finding 40 | Sometimes single-use disposable materials –gloves, surgical masks, aprons and overshoes –were used incorrectly or irregularly, were re-used or were ‘cleaned’ with hand disinfectants. (U) |
| Illustration | During […] care, “hand hygiene”was applied to the non-sterile gloves with [brand name] from a 100 ml container. The gloves are not yet dry when the nurse continues to work . [Observation] (p.6) The nurse wears the pair of [gloves] almost the entire moment of care." [Observation] "The client fails to press the nasal strip of the nasal mask twice. The nurse did not point this out to the client." [Observation] "We just spend six months wearing the same apron with someone." [Focus group] "The [over]shoes, I still see them lying around." [Focus group] The nurse didn't use gloves during the delivery of care." [Observation] "Initially, the nurse does not wear an apron while providing care. The client herself points out to the nurse that when the research is about the prevention of infections, the nurse should be wearing an apron. The nurse then picks up a white disposable plastic apron from the cardboard box with stock on the table. The nurse then sits back on the chair at the kitchen table."[Observation] "Well, then, everyone has to do it. And that's something I run into quite often. That's also with chemo for example, then you come, you hoist yourself into the suit according to protocol and then people say, why do you do that? The others never do that. Well, then you're standing there. That's embarrassing." [Focus group]"According to the guideline, we must always shower with an apron. I don't always do it." [Focus group] (Supplementary table 2 Q7) |
| Finding 41 | A variety of electronic communication devices were used before, during and after the delivery of home-based nursing care. The most frequently used devices were (smart)phones and computer tablets, and these were often used simultaneously. Additionally, devices were constantly being carried around for consulting electronic health records or to examine nurses’ schedules. (U) |
| Illustration | The tablet is carried between two clients under the nurse’s arm. Upon entry, the tablet is placed on the countertop. At the end of the delivery of care, the tablet is again put under the arm on the way out [of the home]. [Observation] (p.6) "That computer, I take it out of that bag, it sits on the table at the client, and it goes back in my bag. And it ends up on the next client's table." [Focus group] "And anyway, using the phone, I think there's still something there. [...]The phone goes from one to the other and out of your pocket, in your pocket." [Focus group] (Supplementary table 3 Q1) |
| Finding 42 | Generally, in offices or in the nurses’ cars, cleaning or disinfecting wipes were available, but the nurses doubted the right ways (method and materials) and times to clean [electronic devices]. Thus, these wipes were used in only a few instances. (C) |
| Illustration | There isn’t actually any kind of guideline which tells you, "This exists, and you can order that. And you use this in that situation, and you use that […] ”That just doesn’t exist. [Focus group] (p.6) The wipes to clean (smart)phones and tablets are from [brand name]. These are 'screen cleaning wipes'. It is unclear whether they also have an antibacterial or disinfecting effect. [Observation] (Supplementary table 3 Q3) |
| Finding 43 | In addition to being physically present during the delivery of care, communication devices also tend to be distracting because they can interrupt nursing procedures. The latter is especially true because nurses feel pressure to answer the phone in case the call involves peer consultations or possible difficulties. (U) |
| Illustration | Especially if that phone is red-hot. […] Sometimes you’re doing twenty things at once. In the meantime, your schedule goes on, so yes, then it often goes wrong […], like forgetting to put on gloves preparing an antibiotic. [Focus group] (p.6) "You do feel like when you're on standby duty you have to answer the phone, and even if you're in the middle of an activity, it's just a reflex." [Focus group] "I notice there's a bit of expectation that we always answer the phone, because we have the nursing homes here. But I've had it once, then I'm standing...Then it wasn't even an aseptic procedure, but then I'm standing with a terminal client, then I suppress the call, because that sound is enormously disturbing and then they just call four, five times in a row until one of us picks up. And then sometimes I just pick up the phone angry and say ‘hello, I don't suppress the call for nothing’. But that... It's just that we're always expected to pick up the phone." [Focus group] (Supplementary table 3 Q5) |
| Finding 44 | Nurses, professional caregivers and clients found that different protocols were given by different institutions, hospitals and colleagues, resulting in fragmentation, variation, discrepancies or conflicting information. […] Furthermore, these nurses, caregivers and clients recognised differences between home-based nursing care teams in dealing with certain situations, guidelines and protocols,[...] At times, these nurses and professional caregivers doubted the accuracy of the information they received. (U) |
| Illustration | "We sometimes notice different protocols for [hospital name] and home care" [Focus group] "Cause we're both working in [place] and we were talking about it on the way here from, yeah, we're doing it this way, and what are you doing? Yeah, it's a bit different from each other, then."[Focus group] "[...] then another client came and she said yes, we always put on those purple gloves and then she said, I don't understand that you do that because those others don't do that. They just have the blue gloves." [Focus group] (Supplementary table 4 Q2 & Q3) Yes, and also in terms of hygiene, hospitals, we notice that they […] sometimes use very different protocols. […] That some protocols are just very different from the [name of organisation] protocol. […] There’s really quite a difference between that and that, yes, if a client is sometimes focused on that, it can sometimes cause quite a bit of friction […]. [Focus group] And […] those protocols don’t [seem to] fit well in home-based nursing either. Those are all questions […]. Can we expect an unambiguous answer? […], we can’t, can we? [Focus group] (p.6) |
| Finding 45 | Working alone made it very difficult for nurses to observe their colleagues or to discuss infection prevention practices. In such cases, the implementation and evaluation of new information or policy changes were problematic. (U) |
| Illustration | “Yeah, it [guideline or policy changes] gets thrown over the fence, and there’s no way to assess whether it’s implemented properly. [Focus group] (p.6) "Look, you're all working individually in the district, of course, and if you keep messing around and nobody's going to correct you." [Focus group] "But then it has to be implemented and that's the step ... Not everyone has the interest in doing that." [Focus group] (supplementary table 4 Q7 & Q8) |
| Finding 46 | Furthermore, a high workload played a negative role in knowledge transfer. Beyond this, the fact that employees are not paid for time spent on knowledge transfer was seen as an impediment. (U) |
| Illustration | Everything the employees get in writing, they have to read on their own time. [Focus group] (p.6) "Work pressure." Yeah. [...] I think that... That it plays a big part." [Focus group] (Supplementary table 4 Q9) |
| Finding 47 | Nurses sometimes experienced untimely or incomplete transfers of clients’ health records when clients were trans- ferred from other care environments to their homes. For instance, this can occur when a client carries a multidrug-resistant organism. (C) |
| Illustration | Moderator: And you already indicated that you would actually like to know when a patient from a hospital or another institution goes home, whether or not he or she is suffering from something [multidrug-resistant organism]. Respondent: Yes. That’s a notorious one. [Focus group] (p.7) |
| **Study: Pogorzelska-Maziarz et al. 2020** | |
| Finding 48 | Specifically, participants reported a certain level of unpredictability during home visits.[…] Staff described attempting to establish clean fields and implement universal precautions in a home, which may lack what they perceived as adequate cleanliness and sanitization. (U) |
| Illustration | “Well, it’s not the hospital where it’s a controlled environment. You’re going into patient’s homes that sometimes aren’t the cleanest. You just gotta do the best you can and try to be as clean and prevent infections as you can in the home. You’re working with what you have.” (p.1784) |
| Finding 49 | The presence of pets and/or pests was also discussed as adding to the variability during the home care visit. (U) |
| Illustration | “It’s hard in home health. Sometimes, I’m just at a loss. How do you make this happen when these people are living in what they’re living in? Because a person can look like they got it all together on the outside, and then you get into their home and hoarding situations, infestations of animals.You just have to start and build trust.” “For me is the home environment. You can be absolutely aseptic the whole time you’re in there. [But] if there’s dog poop 10 feet away from you when you’re doing wound care, that’s a problem. Some of the home environments are not appropriate for certain kinds of patients. There’s no way I can get them healed in that environment.” (p.1786) |
| Finding 50 | There may be evidence of hoarding or the absence of adequate indoor plumbing, which can further complicate healthcare provision in the home. (C) |
| Illustration | It’s hard in home health. Sometimes, I’m just at a loss. How do you make this happen when these people are living in what they’re living in?. Because a person can look like they got it all together on the outside, and then you get into their home and hoarding situations, infestations of animals. You just have to start and build trust.” (p.1786) |
| Finding 51 | Some staff described difficulties with ensuring patient, family, and caregiver compliance with treatments and IPC procedures because home health staff are not present in the home all of the time. (C) |
| Illustration | “As a home health [provider], we are only there X amount of hours every week with the patients. The rest of it is up to them. The rest of it is up to family.” (p.1786) |
| Finding 52 | Participants described staffing challenges faced by home health care agencies because of the aging workforce and poor retention and recruitment. Participants explained how this impacted patient care including adherence to infection prevention and control (IPC) and how it affected current staff who had to take on additional responsibilities. (U) |
| Illustration | “More than 40% of our aides are over the age of 60. . We’re struggling to recruit and replace the workforce.” “[Since] we can’t secure somebody, get them through our training process before they are moving on to work for somebody else, [therefore] our RNs are actually functioning in that CNA role. So, if somebody needs help with bathing, dressing, or grooming, our RNs are picking up that duty.”(p1786) “The nurses I can say, they are just so stressed up. They have too many patients to see, and they want to make sure that they covered everything. Now they are shortcutting, the tendency of shortcutting is you are giving a high percentage of committing mistakes.” (p.1784) |
| Finding 53 | However, despite the focus on IPC, several supervisory staff described specific challenges with clinician adherence to proper hand hygiene protocols and over-reliance of field clinicians on hand sanitizers vs handwashing. (U) |
| Illustration | No quotes. |
|  | Several participants with a role in IPC talked about their lack of formal training, as well as the limited number of resources focused on IPC specifically in home health care. (U) |
| Illustration | "I’d like to have a better grasp of [IPC] myself. I don’t feel like I know enough.” “I actually had no previous experience in home care or formally in infection prevention when I came to this role, which is a challenge . When I look at what resources are out there. [It is] very much geared toward the inpatient world.”(p1786) |
| Finding 54 | Noncompliance was viewed as a major challenge; many participants gave detailed accounts of the lengths they went to teach patients and their families and caregivers about wound care, environmental sanitization, and medication adherence. (C) |
| Illustration | “I think it’s a little harder. for family caregivers to use gloves. We tell them the importance and some may understand the importance of it, but I think, because it’s their loved one, they don’t necessarily have that same level of importance.” |
| Finding 55 | Furthermore, having leadership support and encouragement allowed staff members to take on IPC as a key initiative. (U) |
| Illustration | “My director she’s given a lot of it over to me and allows me to teach and to take control so that I know exactly what my nurses and my therapists have been taught. That’s been very helpful to me, to allow me to take that on as my project because I’m passionate about it.” (p. 1788) |
| Finding 56 | Having an agency dedicate time and money toward staff education (whether preemptive or reactive) was viewed as critical for clinicians to be able to provide quality care to their patients. (U) |
| Illustration | “We’ve come a long way. [Now,] new staff are oriented to the [agency] expectations. We have a higher compliance rate, and we’ve had joint commission surveys where we have not had any infections tags or anything. I know in the beginning, there was a lot of education and reinforcement.” “In the six months I’ve been here, I’ve seen a decrease in infections because of some of the systems that were put into place, the educational assistant, and the things that are on our charting now, our documentation.” (p.1787) |
| Finding 57 | Several participants described specific ways in which agencies have improved teamwork and care coordination. (U) |
| Illustration | “When there is an update or a change in anything, that’s something that we address during that [interdisciplinary monthly] meeting” “[...] the biggest thing that’s helped us is we have secure texting, and now we can all text in a group..It’s easier to communicate” (p.1788) |
| Finding 58 | Finally, some agencies described utilizing real-time data, such as data from their electronic medical records, as a key to success because they were able to direct their often-limited resources to target specific areas for improvement, often related to IPC. (U) |
| Illustration | "We actually are really working just individually right with that team and the team manager to really look at their own outcomes and trying to help them to improve.” “We found last year that our [staff flu vaccination] percentage had dipped from the previous year. We really took that as a quality initiative to really bump up how are we going to increase our rates.We were able to have almost a 12% increase last year based on the quality improvement initiatives we took. We’re carrying that over this year as well.” “Infection control. Well, I would say that it’s a work in progress. Our urinary tract infections have dropped a little bit, but they’re still above the national average. I did have national [data] across the board from all the state. Everywhere, patients have low scores with medications. So, we developed a new tool, and our percent went up 20%. That’s a big deal.” (p.1788) |
| **Study: Bell *et al.* 2022** | |
| Finding 59 | A number of participants viewed their prior training as a contributing factor to their preparedness during the pandemic. They described how frequent contact with patients with infectious conditions prior to the COVID-19 pandemic contributed to a sense of mastery in infection control practices. The education they received prior to the onset of the pandemic provided them with foundational knowledge in infection control practices, particularly around use of personal protective equipment (PPE) and protocols intended to minimize the spread of infection such as hand hygiene. (U) |
| Illustration | “I felt like home health was the biggest health sector that was the most prepared because we are trained. We are followed by Medicare once a year [...] They literally follow us into a patient’s home from start to finish. They meet us at the car. They follow us, and they see how we do things. A lot of it is that the clean technique, and the handwashing, and everything....That part, I felt like I was more prepared than Dr.Fauci. I felt like, ‘Home health rocks. They rocked it.’ I think that’s why there wasn’t a lot of disease spread in the people’s homes because that part, we’ve had,we’ve been training on, and we’ve been tested on constantly.” “In home health, [we have] always used infection control. We were all prepared, and we were always prepared in teaching patients about infection control in their own homes and how to dispose of their dressings and things like that, right? In the community and in the patients’ home we were good at that, and we were prepared for a pandemic.” (p.4) |
| Finding 60 | Some agencies even reported having a stockpile in place due to previous experience with communicable disease outbreaks. (U) |
| Illustration | “PPEs, if you remember back maybe three, four, or five years ago, during the Ebola outbreak, we were prepared. We were able to stack up on gowns, face shields, masks, N95 masks, and that sort. Fortunately, we didn’t have to use those at that time, and when this COVID came in, we were more or less prepared. We were able to actually anticipate it a little bit, placed orders for PPEs back in late January, February, up till March when those things were still available. (p. 4-5) |
| Finding 61 | Yet, not all agencies, or providers felt as prepared. While some agencies did have a pandemic plan, providers were either not familiar with its details or felt their infection control plan fell short given the unknown needs of this novel pandemic. (C) |
| Illustration | “Well, we didn’t have the proper PPE. The emergency preparedness plan didn’t even have a pandemic in it because when you create your emergency preparedness plan, you create it for things that could happen such as a snowstorm, a flood, a fire, a power outage. No one had, really, a really solid pandemic plan for their emergency preparedness plan. We never had a backup of N95s or regular even surgical masks to provide our nurses because it just was never something that we required or needed.” (p.5) |
| Finding 62 | It was also noted that accessing pandemic resources, such as PPE, was a challenge. Home-based care providers described not being well-connected or prioritized in terms of public health resources. Community-based organizations were found to be more available as a support to acquire PPE than government resources. (U) |
| Illustration | “With being a home care provider, we weren’t high up on the supply chain list to get equipment, but without the equipment, we couldn’t walk into a patient’s house, so it was a real catch-22 to where I even had to reach out to a [religious] mission, who actually got us our first PPE equipment.” (p.5) |
| Finding 63 | Home-based care providers and their patients’ experienced frustration around the lack of clear and consistent information from public health officials on health, safety, and wellbeing during the novel pandemic. The delayed timing and execution of pandemic messaging hindered both providers’ and patients’ preparedness. [...] Providers felt they were not able to pass along adequate information to patients, as the information was uneven and, in some cases, conflicting. Likewise, the lack of response from public health authorities left home-based care provider agencies confused on what care measures were appropriate given the uncertainty around the virus. |
| Illustration | “Yeah, I think the most frustrating thing was the guidance from CDC was not clear. Remember how they said, “Mask is not necessary?”...Initially they said, “Well, mask is not necessary––only if you are having fever, cough, then you should wear a mask.” That was kind of bad decision they gave us I think about four weeks later, they started saying, “Everyone should wear a mask.” Again, not really sure though. “We recommend everyone.” Now they’re saying, “It’s mandatory everyone should wear a mask.”[...]. (p.5) |
| Finding 64 | To cope with this lack of information, home-based care provider agencies turned to the Centers for Disease Control and Prevention (CDC) or their own infection control plans; however, the novelty of the virus left many to question what to do to protect themselves and their patients in the onset of the pandemic. (U) |
| Illustration | "[...]I think that this coronavirus-19––even CDC did not have a good sense of how it is, and what’s the use of mask and all that." “The government should do a better job in preparing us to deal with all this. That’s what it is. t’s all coming from them. Whatever they tell us to do—and CMS (Centers for Medicare and Medicaid Services) also. The same thing with CMS because we work with Medicare. It’s all about the guidelines that we follow. [...] Because whatever they think it’s necessary to do—it comes from them. That’s what it is. They have to put out better guidelines, I guess. A better plan, a better preparation. ”“I would say just that we didn’t have all the information on the virus. Usually, as nurses, we know what the diagnosis is and what the side effects are and the symptoms are and how to treat it, right? With COVID, it was we didn’t have definitive information, right? It was difficult to give information because it was evolving, and we were learning as we was going.” (p.5) |
| **Study: Osakwe *et al.* 2021** | |
| Finding 65 | Home health aides (HHAs) provided personal care to patients who had functional limitations. HHAs consistently stated that they had no other health care professional to rely on to help with difficult tasks in patients’ homes, which may require assistance in other settings, such as turning or positioning physically dependent patients. While providing such care, HHAs had fears related to COVID-19 exposure risk. (U) |
| Illustration | “It is difficult because you have to feed the client and take care of them. . .and certain interactions are hard, I try to stay away when I talk to them, but it does not work because some have vision impairment. Social distancing is absolutely impossible because they are completely dependent on you.” “. . .You never know, you have your mask on, but the patient is in your face, remember the patient is bed bound and when you try to help them out of bed, they are in your face. It was the scariest thing. . .only by the grace of GOD. . .it is not a good experience.”(p.1364) |
| Finding 66 | HHAs indicated that they wanted to know if their patient or patient’s family had tested positive for COVID-19 but emphasized that this information was not available. They also expressed concern regarding the lack of information about the COVID-19 status of other HHA colleagues who also care for their patient and how this might put them at risk for exposure to COVID-19. (U) |
| Illustration | “It’s difficult because no one test the family and friends of the patient, no one is testing the patient. You don’t know what the patient has going in, but you have to be in there day in, day out. You don’t know if the patient even has contracted COVID-19. Nobody is testing them.” “I am with a patient, and she has all this different aide coming in and out. No one tells you if an HHA had COVID, I do not know if my patient had it.. . .” (p.1365) |
| Finding 67 | In addition to uncertainty regarding the COVID-19 status of others, HHAs worked with a fear of not knowing their own COVID-19 status because they had not been tested. […] HHAs expressed frustration with the limited information they received from home health care nurses or their agency about getting tested. (U) |
| Illustration | “It was like hell going to work every day. It is difficult, because every day, you are thinking, and you want to know if you have contracting something. It was a torment.” “Nobody is there checking on you or giving you information about test.” (p.1365) |
| Finding 68 | While HHAs expressed concerns about lack of access to testing, many HHAs appreciated the daily screening COVID-19 which their agencies conducted via telephone. (C) |
| Illustration | “No difficulties because the company is well organized, we have cellular communication, apps. Daily, we get tips and techniques on how to stay safe and keep the patient safe. There is a lot we can do. It’s sent to us via internet, and we can take care of ourselves better.” “Every morning there is a recording, with the agency, wash your hands, wear a mask. . . blabla bla that’s all the information we get. Wash your hands, remember to wear a mask when you’re in the presence of the patient. Nothing about the patient. All of us get it. As soon as you clock in that’s what you get.” (p. 1365) |
| Finding 69 | Many HHAs experienced challenges with having an adequate supply of personal protective equipment (PPE) during the pandemic. Some noted structural barriers related to the home health care office being closed when their shifts end, making it hard to get access to supplies. (U) |
| Illustration | “A mask alone is not enough protection to me when I have to be in that home every day. A mask is not supposed to be worn more than 8 hours a day, and you are not given enough. During the weekend, if you don’t go, you don’t get it. You have to go to the office to pick it up. If you work 9-5, and the office closes at 5 pm, when you close from the case, the office is closed. How are you going to get to the office before you go to work? They are not mailing supplies after COVID, you have to go in and get it.” (p. 1365) |
| Finding 70 | Further exacerbating this challenge was the limited availability of adequate hand hygiene products in patients’ homes. (C) |
| Illustration | “[The patient] has a lot of hand bar soap. So, I spoke to the wife, and I told her to please get us hand washing soap like a pump.” (p. 1365) |
| Finding 71 | Among Spanish-speaking participants, gaining access to COVID-19 related information necessary to facilitate patient care was a challenge. Spanish-speaking HHAs preferred working with Spanish-speaking patients. Another HHA expressed how language posed a barrier to effective communication with non-Spanish-speaking home health care nurses. [...] Because of limited English proficiency, Spanish-speaking HHAs had to locate resources to facilitate communication with patients, families and the health care team, and to understand COVID-19 related information. Many Spanish-speaking HHAs thus relied on their family members to translate care plans or clinical information. (U) |
| Illustration | “Don't send me where I can't communicate. The first thing I have to do is communicate with that person.” “almost always, the nurses who arrive speak English, do not speak Spanish. And I don’t speak much English, very little." “When they send it [the information] to me in English, I send it to my daughter. My daughter explains it to me. I was once sent an English case from the same agency.” "To understand a plan of care, it would be good if we were given Hispanic people because who could do it in Spanish. As a Spanish-speaking person you're going into a house, the care plan is in English, and you won't be able to read it.” (p.1365-1366) |
| Finding 72 | HHAs placed high priority on keeping patients safe at home and free from falls. This effort frequently compromised their ability to maintain hand hygiene practices, thus increasing potential COVID-19 risk. (U) |
| Illustration | “You can’t even get up and go to the bathroom. Most of the time, they don’t stay put and they are not steady on their feet, so most of the time you have to be with them. You have to be there with the walker because they will not stay put. . .you can’t even leave to go to the bathroom to wash your hands because she's going to get up and she's going to fall.” (p.1366) |
| Finding 73 | Many HHAs described the unique challenge to keep their masks on in patients’ homes and the conflict with patient satisfaction. Mask wearing made it difficult for patients with hearing impairments to fully understand HHAs when speaking. As a result, HHAs reported being caught between maintaining infection prevention practices at the risk of achieving high patient satisfaction—a factor critical to being retained as a HHA with patients. (C) |
| Illustration | “I keep my mask on 99% of the time. . .She asks me about my mask and says she doesn’t understand what I am saying, I should take it off. I explain and explain why I wear my mask.. . . but she doesn’t like the mask and I tell her it is necessary. If I have to speak clearly, I remove it off my mouth and then put it back. She doesn’t like the mask. It is a challenge.” (p. 1366) |
| Finding 74 | HHAs also feared that their vulnerability to COVID-19 was greater because patients sometimes did not adhere to wearing masks. (C) |
| Illustration | “Patients remove masks, they touch everything. They sit outside. They don’t wash hands at home. . .” (p.1366) |
| **Study: Sterling *et al.* 2020** | |
| Finding 75 | Participants went to great lengths to take COVID-19 precautions while in patients’ homes. They described engaging in elaborate cleaning routines whenever possible during their shifts. (U) |
| Illustration | “I clean like there’s no tomorrow. I wipe down everyurface—the table—the chair. I walk with the little bleach wipes.” (p.1456) |
| Finding 76 | To protect patients, participants went to the grocery store and pharmacy on their behalf, which increased their own risk for contracting COVID-19. Although sometimes they volunteered, other times they were asked. (U) |
| Illustration | “He needs to stay inside the house, so he tells me, ‘I need you to go there, go here.’ I really don’t want to, but I can’t say no. I’m the aide; I’m supposed to do this.”(p.1456) |
| Finding 77 | Participants also worried about their own risk of contracting COVID-19, and nearly all felt that their dependence on public transportation increased this risk. Many participants reported using public transportation to get to their patients’ homes, to run errands for them, and to travel to their agency for supplies. (U) |
| Illustration | “I take 3 buses to get to work: the 9, the 19, and the 5. . . . That's a lot of traveling and different people around.”(p.1456) |
| Finding 78 | Finally, many participants cared for a patient alongside other workers who entered and left the home each day. This added to their fear of transmitting COVID-19 to their patients and to one another. (U) |
| Illustration | “There are 5 of us that work with her. . . . What happens if we all get sick?”(p.1456) |
| Finding 79 | Some participants tried to coordinate hygiene and handoff practices with the other aides caring for common patients. (U) |
| Illustration | No quotes. |
| Finding 80 | Although some agencies adapted quickly to the pandemic by providing workers with COVID-19–related information on a weekly or daily basis, others reportedly barely communicated about the pandemic. (U) |
| Illustration | “Nobody ever told us, ‘you gotta take precautions’ and blah blah; nobody tell us anything.”(p.1456) |
| Finding 81 | Many home health care workers also reported that they lacked adequate PPE from their agencies, including masks and gloves, which they felt was essential for care. (U) |
| Illustration | “I’m worried about getting infected because I don’t have the right equipment. The agency has not really been providing for their workers, at all.” (p.1457) |
| Finding 82 | Participants reported that they had not received COVID-19–specific training from their agencies but had hoped that it would be offered in the future. (U) |
| Illustration | No quotes. |
| Finding 83 | Some agencies asked participants to perform daily “self-assessments.” Self-assessments, which were usually automated by phone, were intended to screen home health care workers for COVID-19 symptoms. Depending on how they answered, workers would be encouraged to go to work or to call their doctor. (U) |
| Illustration | “They text a 4-question screener every day. They want to know if something changes in your body. Do you have a fever? Do you have a cough?” (p.1457) |
| Finding 84 | Taken together, they tried balancing the risks of work with their own health and financial well-being. (U) |
| Illustration | “You have to contribute certain hours to get benefits. . . I have to go out there because I have bills to pay.” “It’s just not a job where you can work from home.”(p.1457) |
| Finding 85 | Many spoke about balancing the risks of caring for patients during the COVID-19 pandemic with the duty or “calling” they felt to help patients. (U) |
| Illustration | “I see a fire. Am I going to walk right into that fire? . . . If I have the backup, the proper gear, yes, I’m going to be there on the front lines to help that person.” (p.1457) |
| **Study: Emmesjö et al. 2022** | |
| Finding 86 | The RN and MICM-physicians [Mobile Integrated Care  Model] worried about infecting patients, co-workers, and themselves, as they initially worked without protective gear. (U) |
| Illustration | No quotes. |
| Finding 87 | The participants explained that at the beginning of the pandemic they were not allowed to wear protective gear in the form of masks or face shields when in close contact with patients because of the scarcity of protective gear, which worried them. (C) |
| Illustration | “We always work with face shields now. We’ve followed the authorities’ regulations. We didn’t use face shields in the beginning, but if we had, maybe we could have avoided infection.”(p.6) |
| Finding 88 | The authorities had stated that this protective gear was unnecessary when working with older patients, and there was a nationwide lack of protective gear. The participants explained how they had been told that protective gear needed to go to hospitals instead, because of the scarcity of it. (C) |
| Illustration | “We always work with face shields now. We’ve followed the authorities’ regulations. We didn’t use face shields in the beginning, but if we had, maybe we could have avoided infection.”(p.6) |
| Finding 89 | Some participants bought their own protective gear when it was not supplied by the municipality or primary health care center. When the protective gear was allowed to be worn, the participants were relieved since it was a relief easing the worry of infection. (C) |
| Illustration | “We always work with face shields now. We’ve followed the authorities’ regulations. We didn’t use face shields in the beginning, but if we had, maybe we could have avoided infection.”(p.6) |
| Finding 90 | After protective gear was permitted, the way RNs and MICM-physicians worked changed. Protective gear was used constantly when the RNs and MICM-physicians were within two meters of a person during work, and the participants became less worried about infecting someone or being infected themselves. (C) |
| Illustration | “We always work with face shields now. We’ve followed the authorities’ regulations. We didn’t use face shields in the beginning, but if we had, maybe we could have avoided infection.” (p.6) |
| Finding 91 | To prevent transmission, the participants explained that they followed the authorities’ restrictions, even if the recommendations changed often. The changing directives were seen as challenging, but the RNs and MICM-physicians worried less about transmission if they knew that they were following up-to-date recommendations. The MICM-physicians or RNs held information or visited personnel meetings to answer questions from the ANs [assistant nurses] about COVID-19, as the participants noticed that the ANs needed additional l knowledge to ease their worries about infecting others. (C) |
| Illustration | “We want the personnel to feel safe when they work with the patients, to not be afraid. We got this.”(p.6) |
| Finding 92 | The participants explained that they had been liberal with testing to ensure that the virus did not spread and to ease worry. The RNs and MICM-physicians [...]spoke about how they had tried their hardest to constrain infectivity by following the changing restrictions. (U) |
|  | No quotes. |
| **Study: Tavemark et al. 2022** | |
| Finding 93 | F8.1 At meetings, employees received practical information and were shown how to use protective equipment, which was clearer than just reading the instructions. They were also given training on COVID-19, they were tested on basic hygiene and shown films on handling protective equipment to ensure their competence. It was time-consuming for the staff but they perceived it as good to be able to update their skills.  (U) |
| Illustration | No quotes. |
| Finding 94 | To get information about the spread of infection in the municipality, the staff had a review of the infection situation at the unit every morning and afternoon. At the daily meetings, the content of the day’s home visits was prioritized with the support of the manager, something that was perceived as valuable, especially at the beginning of the pandemic. At these meetings, it was also verified that everyone had been informed in the event of an infection. Nurses and unit heads had a great responsibility, but it was not clear who they should inform. (U) |
| Illustration | “It is always a problem when you get a positive [case], how the infection tracking should be done and who you are supposed to notify.” (p.4) |
| Finding 95 | Despite the fact that the staff received information, they did not always comply with the routines during the pandemic. The difficulties were often attributed to a lack of communication and rapidly changing routines. (U) |
| Illustration | No quotes. |
| Finding 96 | Licensed staff said that communication about the spread of infection was lacking and that the assistant nurses did not always use face masks in a correct way and worked in private clothes. (C) |
| Illustration | “The managers didn’t give support, they think we should carry on as usual as long as everyone was healthy.” (p.4) |
| Finding 97 | During the pandemic, guidelines and routines gradually changed and the participants commented that it took too long before the restrictions were tightened. Routines for disinfecting shared surfaces such as sinks, computers, telephones, and keys were clarified. The new guidelines for the home care environment meant that staff were expected to wear work clothes throughout the work shift and use face masks in the office when there were several colleagues on the premises. (U) |
| Illustration | No quotes. |
| Finding 98 | According to the guidelines, there should be sterile surfaces in connection with cleaning of protective equipment. Sometimes it could be difficult to find good countertops, as they could be made of wood, be dirty, or have grooves; in these cases, plastic trays were bought to facilitate disinfecting the visor. Often, a place was prepared in the hall, and sometimes the kitchen was used. The kitchen might be located at the far end of the home, which meant that the employee had to walk through the entire house without protective equipment before or after the intervention with the older adult. (U) |
| Illustration | No quotes. |
| Finding 99 | During the pandemic, the employee would always wear protective equipment in the older adult’s home, but in the beginning there was no equipment. |
| Illustration | No quotes. |
| Finding 100 | The routines for the protective equipment were interpreted differently and changed rapidly. There were differences in which protective equipment was used and how it was handled, whether it was put on in or outside the home, and where the material was then disposed of or cleaned. Using the protective equipment caused physical problems in the work environment. Participants said that they needed to manage it outside the home and it could be cold to clean the equipment in the cars in winter. (C) |
| Illustration | “In the winter we go in short sleeves (. . .). We don’t wash at their home, so we have had water in the car and soap and sanitizer and then minus 17 degrees on top of that. It has been really difficult. (p.5) |
| Finding 101 | The protective equipment also affected the encounter with the older adult. All professionals emphasized that the care recipients commented that it was difficult not being able to see who was behind the mask. They could become anxious and had difficulty recognizing the staff. One participant described similar difficulties for care staff with hearing loss, as both the older adult and the employee’s colleagues had face masks during home visits. This employee therefore chose a visor instead of a face mask, so that lipreading was possible. (C) |
| Illustration | “They do not see our mouths, they only see our eyes and they can’t keep track of us in the same way (. . .). Now everyone merges into one, so I understand that those with dementia who live at home may find it uncomfortable when we put the visor on. (PT3) (p.5) |
| Finding 102 | It was clear that the participants saw the risk of becoming infected as part of their work. (U) |
| Illustration | "In November I got infected, but I’m not really worried, it’s more natural because of my work, I can’t do much more.” (AN9) (p.9) |
| Finding 103 | To deal with staff concerns, managers were quick to initiate infection tracking, share information from the employer, and offer support conversations. In the smaller municipality, there were quick tests at the workplace that they could choose to use in connection with each work shift, which were perceived as providing security.  (U) |
| Illustration | No quotes. |
| Finding 104 | The longer the pandemic lasted, the more they followed the hygiene guidelines and kept their distance automatically, which meant that they relaxed and their anxiety decreased. It also decreased as more people in the community were vaccinated. (U) |
| Illustration | No quotes. |
| Finding 105 | The protective equipment contributed to a feeling of security for the staff in their work at the older adults’ homes. But the care recipients’ worry increased when the local newspaper wrote that staff did not use protective equipment correctly. (C) |
| Illustration | “I feel really sorry for the patients where a lot of home care staff are running around who don’t give a damn about this protective equipment. (. . .) They are then really vulnerable because they still need to receive help.” (RN4) (p. 7) |
| Finding 106 | Participants said that the infection sometimes came via relatives. But there were also participants who said that assistant nurses infected the older adult, which angered the relatives because they had struggled with isolation. (U) |
| Illustration | No quotes. |
| Finding 107 | It emerged that assistant nurses with symptoms were encouraged to work because it was difficult to get temporary substitutes. (U) |
| Illustration | No quotes. |
| **Study: Moi e et al. 2022** | |
| Finding 108 | Nurses experienced a significant lack of information and explicit guidelines at the start of the pandemic. When the public guidelines emphasized the extensive benefits of using a face mask, the nurses were instructed not to use one. (U) |
| Illustration | “… so, there was very much uncertainty in the group since some heard that some districts do this, and some districts do this and here we don’t do either. Why and what and who should decide how to deal with this? And what can we do here?” [Nurse 11] (p.3) |
| Finding 109 | Significant uncertainty was prevalent both nationally and locally and the uncertainty was reinforced in their specific work situation. (U) |
| Illustration | “Heard on the radio on the way to work. The Assistant Minister for Public Health said that face masks should be used in home-care nursing, but we did not have any routines in place related to the use of face masks” (p.3) |
| Finding 110 | One challenge that was particularly prevalent at the start of the pandemic was the lack of equipment. One nurse told how they ordered huge amounts of infection control equipment and yet received only small numbers. (U) |
| Illustration | “We ordered 500 facemasks. We received 25. It was impossible to get a hold of … it was a real blow to us … glasses and visors were impossible to get a hold of. We had to go to Europris (a cheap store chain in Norway) and buy protective glasses used for fireworks. That was the level we were at!” (p.4) |
| Finding 111 | As well as challenges associated with obtaining enough equipment, our participants experienced the importance of how their immediate superior and their district manager acted after the outbreak of the pandemic. Many had positive experiences with their manager regarding how they dealt with the challenges. (U) |
| Illustration | "They have been very available to us, and there for  us. They have kept us well informed, they made sure we were given the necessary amount of input and training. We had training days on the internet where the focus was on infection control” [Nurse 4]. (p.4) |
| Finding 112 | When patients asked for more closeness, the nurses experienced this to be difficult. (U) |
| Illustration | "You don’t need to use a face mask with me, because I will be dying soon anyway" (p.5) |
| Finding 113 | The participants had gained a much better understanding of infection control than previously, in addition to the increased attention from the wider society. Infection control routines were drawn up in all the districts and participants felt the routines to be clear. They had gained knowledge and experience that gave them increased confidence to confront new pandemics. |
| Illustration | “We have all achieved a better understanding of hygiene, that we need to be far more careful in following the rules. This we have really learned!” [Nurse 3] (p.6) |
| **Study: Baumbusch et al. 2022** | |
| Finding 114 | A new role for caregivers was supporting their relative to follow public health measures. Wearing masks and handwashing became part of everyday life during the pandemic but supporting a person living with dementia to follow these guidelines could be challenging. (U) |
| Illustration | "the biggest issue is trying to get him to remember to wear his mask and not to hug people or shake their hands? He’s just got his hand out there” (p. 187) |
| Finding 115 | Changing lifelong social practices was a difficult aspect of public health measures, as was building mask wearing into their daily routines. (C) |
| Illustration | “He’s getting much better at remembering to at least put his mask on. I try to make sure that he’s got one. He keeps a disposable blue one in his back pocket just in case he forgets and I forget to make sure he’s got one. I try to keep some in the car.”  (p. 188) |
| Finding 116 | However, the constant teaching and reminders regarding self-protection protocols further complicated daily routines and exacerbated caregiver stress. (U) |
| Illustration | "Getting the hands washed and getting the mask off and then getting the hands washed again, all those things are difficult . . . it can be a bit of a trigger." (p.188) |
| Finding 117 | Another facet of this issue was managing staff’s adherence to public health measures when they came into the family’s home. (U) |
| Illustration | “I always walk them to the sink to wash their hands when they come in. And I have paper towels available beside the sink. And I have Lysol wipes in the bathroom in case anybody has used anything in there. So, I think I’ve set up the house as best I can. (p.188) |
| Finding 118 | Because the staff were employed by the local health authority, they were provided with personal protective equipment (PPE) that they were expected to wear while inside client’s home. Managing staff’s use of PPE is another additional role for caregivers during the pandemic. (U) |
| Illustration | Oftentimes one of the first things they’ll ask you is, “Do you mind if I take the goggles off?” I don’t particularly mind. But I would like them to keep the mask on. And it shocked me when I’d come home where they’ve had it down. I recognize like if you’re doing crosswords and stuff, it can be hard to understand, but I’ve got other games. You can read, you can play Bingo, you know, whatever. But really, I don’t think they have any business taking their masks off . . . I just ask them to put it back on, on their face. (p. 188) |
| Finding 119 | Caregivers described the additional economic burden of the pandemic measures. This situation highlights one way in which pandemic response measures disproportionately impacted people with low incomes, who are often older people with fixed incomes. (C) |
| Illustration | "I’m out there sourcing on N95 masks which are an arm and a leg when the pandemic starts, and getting the family and getting gloves, sanitizer and all this kind of stuff. I’ve been wiped on wipes. I’ve got a few for my parents who are low income." (p. 188) |
| Finding  120 | There was also scarcity of supplies that they were already using, and now stores were limiting purchases or sold out entirely. She also shared how stores increased prices in response to pandemic demand. (U) |
| Illustration | "But [stores] all juggle in price. A lot of supplies started to go way up and some of those prices still haven’t come down." (p. 188) |
| **Study: Franzosa et al. 2022** | |
| Finding 121 | Aides’ infection prevention tasks also intensified as they took on more rigorous cleaning and sanitizing and maintained cleaning supplies and PPE for themselves and the veteran, as well as reporting on veterans’ and their own symptoms to the agency. (C) |
| Illustration | "I take precautions. I wear my mask. I keep sanitizer on me. I wash my hands constantly but I still give them the attention they need, you know, it’s very hands-on." (p. 1834) |
| Finding 122 | However, aides noted that the intimate nature of their work made it impossible to maintain physical distance, and this was a source of constant “worrying”. (C) |
| Illustration | "I take precautions. I wear my mask. I keep sanitizer on me. I wash my hands constantly but I still give them the attention they need, you know, it’s very hands-on.” (p. 1834) |
| Finding 123 | Although most administrators reported having sufficient supplies of PPE, some aides described shortages or difficulties obtaining it. (U) |
| Illustration | Her agency "is giving me like, five masks, and that’s it. So, I don’t even think about it, just bring my own bag, I bring my own gloves, my mask, everything.” (p. 1834) |
| Finding 124 | Aides recognized patients’ anxieties over potential infection and took proactive steps on their own to make them feel safer. One aide agreed to wear extra PPE and two masks, while another described undergoing private COVID-19 testing every 2 weeks even though it was not required so she could reassure her client that she was not carrying the disease. (U) |
| Illustration | I11.4 “I show [the veteran] the paper work and say, look, I just want you to feel a little at ease that, you know, I’m negative [so] they don’t have that stress,” she explained. (p. 1834) |
