# Supplementary material for: Risk factors for infection in older adults with home care: a mixed methods systematic review with meta-analysis
Source: BMC Public Health. 2025 May 3;25:1643. doi: 10.1186/s12889-025-22538-1 (PMC12048934; doi:10.1186/s12889-025-22538-1)
Supplement: Supplementary file 10 — Supplementary Material 10 [file 12889_2025_22538_MOESM10_ESM.docx]

Appendix 11. Aspects restricted to either the qualitative or the quantitative findings

***Aspects of the qualitative findings that were not examined in the quantitative studies***

This section highlights categories of each synthesized qualitative finding that were not covered in the quantitative studies.

All categories of the first synthesized finding were supported by quantitative studies. Whilst categories 5 and 6 of the second synthesized finding was supported by evidence from quantitative studies, some other categories identified in the qualitative studies had not been examined in the quantitative studies. For instance, category 4 showed that the home environment itself represented infection risks in terms of as clutter, dirt, pest infestations, inadequate ventilation, and clean water access. Category 7 demonstrated that animals in the home posed infection risks, both by their presence and through their impeding staff’s delivery of safe care. None of these aspects were examined in any of the quantitative studies.

Similarly, congruence was observed between synthesized finding 3 and the quantitative study findings regarding categories 8 and 9, which revealed that staff’s adherence to safe procedures and equipment management influenced infection risks, in line with quantitative evidence that organizational factors such as caregivers needing training increased infection risks. From this synthesized finding it also emerged that other aspects of staff’s experiences were of importance for infection risk but these were not addressed in the quantitative studies.

Synthesized finding 4 revealed that home care agencies hold key responsibilities for infection prevention, e.g. through information and equipment provision to staff. Whilst category 11 was in congruence with quantitative data showing that policies and guidelines for infection prevention impact infection risks, several other dimensions of agency level effects on infection risks were not addressed in the quantitative studies. As such, access to covid-19-tests and PPE, which emerged as central for home care providers in terms of infection control, were not directly examined in the quantitative studies. Besides, the quantitative findings did neither directly address agency level infection control co-ordination and preparedness, nor provision for staff training and knowledge transfer, although these themes emerged as crucial for infection control in the qualitative findings.

Lastly, synthesized finding 5 revealed that home care staff believed that infection risk was inherent in the home care work itself, and therefore, to some respect, unavoidable. This dimension of attitudes and beliefs about infection risk in the home care setting was not addressed in any of the quantitative studies.

***Aspects of the quantitative findings that were not explored in the qualitative studies***

Some aspects of the quantitative findings had not been explored in the qualitative studies and thus not presented in the synthesized findings of qualitative data. For example, findings on individual risk factors such as gender and ethnicity presented in the quantitative studies were not explored in the qualitative studies. Although the evidence regarding sex was inconclusive (suggesting both female and male sex increases infection risks), the quantitative findings suggested that white ethnicity constituted a risk factor for infection. Further, a range of medical conditions were shown to be associated with increased risk of infection in the quantitative studies whereas the qualitative studies did not go into such details using different type of data, yet discussed the presence of comorbidities and general health status as important aspects of increased infection risks. Some environmental risk factors that were identified in the quantitative findings were not directly explored in the qualitative findings, for example living in a congregate setting, though congruity did emerge in terms of living with others and having caregivers available.
